# Counter‐narratives for the prevention of violent radicalisation: A systematic review of targeted interventions

**DOI:** 10.1002/cl2.1106

**Published:** 2020-08-12

**Authors:** Sarah L. Carthy, Colm B. Doody, Katie Cox, Denis O'Hora, Kiran M. Sarma

**Affiliations:** ^1^ School of Psychology, National University of Ireland Galway Galway Ireland

## Abstract

**Background:**

In the field of terrorism research, the violent radicalisation of individuals towards perpetrating acts of terror has been the subject of academic enquiry for some time. One core focus by social scientists has been the role of narratives in this process. Narratives have the ability to present a socially constructed version of reality which serves the interest of the narrator(s). In the context of terrorism, by depicting violence as a viable antidote to individual vulnerabilities, the narratives purported for propagandistic purposes have the potential to thwart perceptions of instrumentality (a key characteristic of violent radicalisation). In order to prevent this from happening, researchers and counter‐terrorism practitioners have increasingly sought to explore the potential for *counter*‐narratives; targeted interventions that challenge the rationalisation(s) of violence purported in dominant narratives which, in turn, reconstructs the story. However, there is overwhelming consensus in both government and academic spheres that the concept of the counter‐narrative is underdeveloped and, to date, there has been no synthesis of its effectiveness at targeting violent radicalisation‐related outcomes.

**Objectives:**

The objective of this review was to provide a synthesis of the effectiveness of counter‐narratives in reducing the risk of violent radicalisation.

**Search Methods:**

After a scoping exercise, the literature was identified through four search stages, including key‐word searches of 12 databases, hand searches of reference lists of conceptual papers or books on the topic of counter‐narratives, as well as direct contact with experts and professional agencies in the field.

**Selection Criteria:**

Studies adopting an experimental or quasiexperimental design where at least one of the independent variables involved comparing a counter‐narrative to a control (or comparison exposure) were included in the review.

**Data Collection and Analysis:**

Accounting for duplicates, a total of 2,063 records were identified across two searches. Nineteen studies across 15 publications met the inclusion criteria. These studies were largely of moderate quality and 12 used randomised control trial designs with varying types of controls. The publication years ranged from 2000 to 2018, with the majority of studies published after 2015. The studies represented a range of geographical locations, but the region most heavily represented was North America. In most cases, the dominant narrative(s) “to‐be‐countered” comprised of hostile social constructions of an adversary or “out‐group”. The majority of studies challenged these dominant narratives through the use of stereotype‐challenging, prosocial, or moral “exemplars”. Other techniques included the use of alternative accounts, inoculation and persuasion.

**Results:**

In terms of risk factors for violent radicalisation, there was some disparity on intervention effectiveness. Overall, when pooling all outcomes, the intervention showed a small effect. However, the observed effects varied across different risk factors. Certain approaches (such as counter‐stereotypical exemplars) were effective at targeting realistic threat perceptions, in‐group favouritism and out‐group hostility. However, there was no clear reduction in symbolic threat perceptions or implicit bias. Finally, there was a sparse yet discouraging evidence on the effectiveness of counter‐narrative interventions at targeting primary outcomes related to violent radicalisation, such as intent to act violently.

**Authors' Conclusions:**

The review contributes to existing literature on violent radicalisation‐prevention, highlighting the care and complexity needed to design and evaluate narrative‐based interventions which directly counter existing, dominant narratives. The authors note the challenges of conducting high‐quality research in the area, but nonetheless encourage researchers to strive for experimental rigour within these confines

## 1 PLAIN LANGUAGE SUMMARY

### 1.1 Counter‐narrative interventions may affect some risk factors related to violent radicalisation, but there is no effect on intent to act violently

Counter‐narratives may affect certain risk factors for violent radicalisation, including realistic perceptions of threat, in‐group favouritism and out‐group hostility. However, the effects are inconsistent across outcomes, failing to target symbolic threat perceptions, implicit bias or intent to act violently.

While the findings from this review support the feasibility of the concept more broadly, they also highlight the care and complexity needed to design and implement effective counter‐narratives in the context of violent radicalisation.

### 1.2 The review in brief

Narratives which reduce complex, real‐world phenomena to simplistic, violence‐promoting propaganda can activate the necessary mechanisms for violent radicalisation to occur. To stop this from happening, researchers and counterterrorism practitioners have turned to *counter*‐narratives; targeted interventions that challenge the instrumentality of violence as put forth in dominant narratives.

This review summarises the available evidence on this approach, looking at whether counter‐narratives are effective at preventing violent radicalisation across a spectrum of contexts, including right‐wing, ethnic and religious extremism

### 1.3 What studies are included?

This review includes studies that evaluate the effects of counter‐narrative interventions in individuals exposed to a dominant narrative which, if not countered, may promote a violent extremist belief system. The outcomes targeted by the intervention include the intent to act violently, as well as “risk factors” for violent radicalisation.

Nineteen studies met the inclusion criteria. These studies span the period 2000–2018 and mainly include study populations of University and high school students. Although the studies represent a range of geographical locations, the majority were conducted in North America.

Twelve of the studies are moderate‐high quality randomised controlled trials and the remainder are quasiexperimental studies.

**What is the aim of this review?**

This Campbell systematic review examines the effects of counter‐narrative interventions on primary and secondary outcomes relating to violent radicalisation. The review summarises evidence from 19 independent studies, including 12 randomised controlled trials. The majority of the included studies are from North America.

### 1.4 Do targeted counter‐narrative interventions work on violent radicalisation?

Counter‐narrative interventions which target a specific, dominant narrative can have an effect on certain risk factors for violent radicalisation. However, these effects vary according to intervention‐type, as well as outcome targeted.

Using counter‐stereotypical exemplars, alternative narratives and inoculation techniques (eliciting resistance through the production of counter‐arguments) were all found to reduce overall risk factors for violent radicalisation. Persuasion did not have a significant effect.

The most pronounced effects were for secondary outcomes (i.e., risk factors), which included realistic threat perceptions towards an adversarial group, in‐group favouritism and out‐group hostility.

Evidence on the effectiveness of the intervention at targeting primary outcomes (such as intent to act violently) is inconclusive.

### 1.5 What do the findings of this review mean?

The concept of using a communication strategy to directly counter a dominant narrative, while intuitive, likely requires a great deal of theoretical complexity in order to work effectively in the area of counter‐terrorism.

Nonetheless, the targeted counter‐narrative approach shows promise. With the emergence of further, rigorous research, the extent of its ability to effectively prevent violent radicalisation will become clearer.

### 1.6 How up‐to‐date is this review?

The review authors searched for studies up to August 2018.

## 2 BACKGROUND

### 2.1 The problem, condition, or issue

Since the earliest days of researching extreme violence, academics and counter‐terrorism practitioners have increasingly sought to better understand the process(es) by which an individual comes to perpetrate an act of terror. In recent years, these efforts have grown into a multi‐disciplinary pursuit, embracing methodologies, as well as theoretical insights, from psychology (Horgan, 2005), psychiatry (Melle, 2013), political science (Bjorgo, 2005), anthropology (Atran, 2006), sociology (Turk, 2004) and communication science (Archetti, 2013; Braddock, 2014). However, despite these contributions, conceptually, the phenomenon has been poorly defined, leading to extensive academic debate (see Schmid, 2004; Schuurman, 2018) with terms such as “extremism” and “violent extremism” emerging, somewhat, as a tonic for this ambiguity.

While support for “extreme” politics carries with it some negative connotations (interpreted as indicative of dwindling support for democratic values, see Knigge, 1998), the term itself is not synonymous with violence. Instead, it is best understood as a belief system existing at the poles of society's central tendency. From here, familiar belief systems such as “far‐right”, “far left” and “single‐issue^1^” politics arise. While a clear incline towards in‐group favouritism and out‐group hostility seems to be a tenet of an extremist belief system (Baron, Crawley, & Paulina, 2003; Hogg, 2014; Kruglanski, Pierro, Mannetti, & De Grada, 2006), it is the unwavering, perceived instrumentality of violence against an out‐group that graduates it to *violent extremism* in most academic spheres (Berger, 2018; Webber et al., 2018). Violent extremism can manifest in several ways, including targeted assault, armed robbery, destruction of property and kidnapping (Jasko, LaFree and Kruglanski, 2017). One particular manifestation of violent extremism is *terrorism* (UNHCR, 2016, p. 9). In an act of terror, the culpability of the victim is entirely removed through the intentional, or threatened, use of violence against civilian targets in order to achieve political aims (Ganor, 2002, p. 294). While there are several variations of this definition, among the most salient characteristics is the exploitation of audience reactions; the eyes of world watch as the message, that one is not safe, is delivered in a “theatre‐of‐terror” (Weiman, 2008, p. 70), the consequences of which claim approximately 21,000 lives each year.^2^

In an attempt to reduce the likelihood of individuals engaging in terrorism, research efforts have moved towards understanding what happens *before* an individual reaches such a climactic point. This shift has sparked investigation into various precipitating factors, such as the onset of insurgency (O'Neill, 2005), conflict (Newman, 2006) and even the increased variability of global temperatures (Fjelde & von Uexkull, 2012; Miles‐Novelo & Anderson, 2019; Price & Elu, 2016) that may serve as “triggers” for the political, sociological and, ultimately, psychological changes that cultivate a violent extremist mentality. It has been argued, however, that this mentality cannot be explained by precipitating factors alone (Kruglanski, Bélanger, & Gunaratna, 2019) and is dependent, instead, on the activation of certain, psychological mechanisms that trigger a cognitive shift; the process of violent radicalisation.

#### 2.1.1 Violent radicalisation

In order to understand violent radicalisation, it is first necessary to understand its “reverse”. Similar to the central tendency of middle‐politics, individuals also inhabit a cognitive middle‐ground of psychological moderation. Kruglanski et al. (2019) describe this cognitive middle‐ground as a condition of homeostasis; “a balanced satisfaction of the individual's basic biological and psychogenic needs” (p. 117). If this balance is tipped by, for example, a threat to one's self‐esteem (Crocker & Luhtanen, 1990; McLeod, 2007), autonomy (Deci & Ryan, 2000), competence (White, 1959), self‐worth (Crocker & Wolfe, 2001), meaning (George and Park, 2016; Martela & Steiger, 2016), or other needs for personal significance (Jasko et al., 2017), unpleasant feelings such as cognitive dissonance (see Elliot & Devine, 1994) can arise. Moghaddam's “Staircase Model” (2005) and Gill's “Pathways Model” propose that certain, external factors such as “catalyst events” (Gill, 2007, p. 173) or perceived injustice can induce these feelings, also. From a psychological perspective, this then requires a certain amount of cognitive restructuring in order to “move” the individual away from these feelings of uncertainty towards a narrower, unambiguous state of clarity (Horgan, 2008).

As differentiated by McCauley and Moskalenko (2017) in their “Two Pyramids Model”, these feelings can occur independent of violent action, and it important to note that this experience is neither indicative nor predictive of violent intentions. Rather, it creates a “perfect storm” for individual vulnerabilities to be exploited, oftentimes through the promise of a remedy. The individual can find themselves presented with a *goal*, a *means*, and a thwarted perception of how the two can be configured (Pieters, Baumgartner, & Allen, 1995). The process of “adopting” a new belief system to create a means‐goal configuration (Kruglanski, Chernikova, Babush, Dugas, & Schumpe, 2015) that addresses this point of conflict is referred to as *radicalisation* (Silber & Bhatt, 2007, p. 16).

During radicalisation, the means of achieving certain goals may be inflexible (Zhang, Fishbach, & Kruglanski, 2007) and even violent or “counterfinal” (perceiving a means as instrumental based on its destructiveness, see Schumpe, Bélanger, Dugas, Erb, & Kruglanski, 2018); as such, seemingly bizarre means such as kidnapping, bomb‐making or seizing an aircraft can be rationalised in its attainment. What began as movement from a condition of homeostasis, employing placid means, is now represented as a similar shift, but using violent means. Drawing together these concepts, *violent radicalisation* can be understood as a departure from cognitive homeostasis, during which a specific need or goal rises in saliency (to the point of rejecting all others) and *violent* means against a perceived out‐group are perceived as instrumental to its attainment (Kruglanski et al., 2019, p. 113).

#### 2.1.2 Violent extremist narratives

According to Significance Quest Theory (Kruglanski et al., 2014), during violent radicalisation an individual's perception of this means‐end configuration can be manipulated through the use of persuasive propaganda; deliberate, systemic attempts to manipulate cognitions, and shape behaviour, in line with the desired intent of the propagandist (Jowett & O'Donnell, 2012, p. 6; Payne, 2009; Winter, 2015). However, the perpetration of an act of terror is a “hard sell”, and difficult to endorse without ill‐supported, “simplistic and direct connection between causes and effects” (Black, 2001, p. 129). One way this can be achieved is through the use of *narratives*.

Narratives here refer to recollections of events which happen in sequence (Barthes & Duisit, 1975; Genette, 1982) with characters that can cause changes (Richardson, 2002). These events and characters are contained within an identifiable beginning, middle and end (Hinyard & Kreuter, 2007, p. 778), in which the sequence (i.e., inclusion or exclusion) of certain events or “independent clauses” is interpreted as *the* order of events (Labov, 2006, p. 1). The objective is to present a social construction of the world which serves the interest of the narrator. However, not all narratives are told to other people, or can be temporally tracked.

The “narrator principle” (Sarbin, 1986) posits that people use narratives to “impose” (Crossley, 2000, p. 532) structure on their experiences. While evidence of a particular, dominant narrative “within” an individual is an elusive prospect, such perspectives claim that narrative structure is pre‐existing, and evidence of particular narratives can be observed through one's “narrative identity” (McAdams, 2001). This comprises of “characterizations” (protagonists and antagonists), “key scenes” (e.g., high point, low point, turning point etc.) and the “selection and interpretation” of events (pp. 108–110). Ultimately, these indicators serve as a window into an individual's internalised and evolving social construction on any given experience. In other words, dominant narratives may not appear as complete, narrative constructions but, rather, as semblances of a narrative identity. In many contexts, these dominant narratives can be beneficial; for example, in the case of birth stories (Callister, 2004), recovery from addiction (Hanninen & Koski‐Jannes, 1999) or, in the context of illness and disease, as a means of coping (Tighe, Molassiotis, Morris, & Richardson, 2011), maintaining hope (Bruner, 1987) and even attributing difficult experiences to a predestined path (Qureshi, 2010, p. 282) or “quest” (Good et al., 1994, p. 838). However, in the context of violent radicalisation, socially constructed narratives may serve more sinister functions.

Narratives purporting violent extremist ideologies posit that the group's goals can *only* be achieved through violence against the out‐group, whomever they may be. Through different methodologies, these socially constructed narratives have been explored across a range of violent extremist and terrorist groups such as al Qaeda (Halverson, Corman and Goodall, 2011; Schmid, 2014), Al‐Shabaab (Joosse, Bucerius, & Thompson, 2015), the so‐called Islamic State (ISIS; El Damanhoury, Winkler, Kaczkowski, & Dicker, 2018; da Silva & Crilley, 2017; Ingram, 2016; Pearson & Winterbotham, 2017), the Animal Liberation Front (Braddock, 2015), Neo Nazi (Poppi & Gattinara., 2018) and far‐right groups (Kundnani, 2012; Pautz, 2014), as well as groups originating from separatist movements in the Philippines (Vergani, 2014), Ukraine (Katchanovski, 2016) and Northern Ireland (McAuley & Ferguson, 2016; Morrison, 2016).

As an example, the following is a popular religious narrative found in numerous religious texts (including the Qur'an and The Torah):

Moses, having pleaded with The Pharaoh of Egypt to release the Hebrews and accept the One True God, threatened Pharaoh with divine retribution. The Pharaoh was arrogant and ignored Moses' warning. As promised God punished Pharaoh with several disasters such as drought, famine, disease, locusts, lice and frogs brought upon his own people.

Here, the murder of innocent people (retribution for not accepting a particular worldview) is perceived as an instrumental means of achieving the overall goal of building a world that recognises the One True God. Therefore, the means‐end configuration has been manipulated to justify violent action. Similar “Doomsday” or “End of Times” narratives have been recycled and purported by the Far Right (Pautz, 2014), the Far Left (Taylor, 1998, p. 7–10) and even among apparently disparate Islamist extremist groups such ISIS (McCants & McCants, 2015) and the Taliban (Ingram, 2015).

In cross‐disciplinary research, there is a growing body of evidence demonstrating that narratives such as these are among the most effective forms of persuasion, and attitude‐change (Shen, Sheer, & Li, 2015; Braddock & Dillard, 2016), likely due to their ability to impede counter‐arguing (see the “Transportation‐Imaginary Model”, Green & Brock, 2002), and, therefore, resistance to persuasion (see the “Overcoming Resistance Model”, Moyer‐Gusé, 2008). In this way, violent extremist narratives can achieve attitude‐change through a process of persuasion, serving as violent radicalisation “triggers” (Wilner & Dubouloz, 2011, P. 433). However, the question remains, how can this knowledge be channelled into solutions?

### 2.2 Description of the intervention

In their review of strategies to stop violent radicalisation from happening, Briggs and Feve (2013) proposed a strategy of challenging such narratives, by deconstructing, discrediting and “demystifying” (p. 6) the themes they purport. This deconstruction falls under the umbrella term “counter‐narrative”. By discrediting their respective dominant narratives, counter‐narrative strategies are intended to present individuals with alternative social constructions to those presented by the dominant narrator. In counter‐terrorism, it follows a logic of prevention; by treating the risk of violent radicalisation through narratives *up*stream, incidence and prevalence of violent extremism and terrorism *down*stream will be reduced.

This idea is not novel. The approach has been explored in relation to challenging inaccurate historical narratives (“counter‐factual” narratives, see Mordhorst, 2008), as well as persistent, socially constructed hegemonic narratives relating autism (autism as neurodiversity, rather than disease, see Broderick & Ne'eman, 2008), infertility (maintaining a narrative of femininity in narratives of failed IVF; Bell, 2004), and disability (challenging dominant societal scripts that reduces disability, fostering exclusion, see Harter, Scott, Novak, Leeman, & Morris, 2006, p. 12). Here, the counter‐narrative is described as “counter‐storying”, designed to offer a narrative identity which resists those emerging from dominant discourse(s) (Ingamells, 2016, p. 58). The approach has also been applied to violence prevention more broadly. In 2004, in response to increased homicide rates, the World Health Organization recommended the implementation of media campaigns as a way of changing “attitudes, behaviours, and social norms” (p. 16) with regard to violence, leading to the development of numerous violence prevention interventions rooted in the concept of the counter‐narrative.

#### 2.2.1 Defining counter‐narratives

Unlike other forms of counter‐messaging such as alternative narratives or government strategic communications (Briggs et al., 2013), it is generally agreed that counter‐narratives should address the underlying logic of a dominant narrative. However, there is little conceptual consensus beyond this point. According to Ramsey (2012), a counter‐narrative is defined by the “countering” aspect (i.e., argumentation). This view is shared by The Quilliam Foundation (Hussain & Saltman, 2014, p. 5) and Briggs et al. (2013) who suggest that counter‐narratives should “pick apart” the messages espoused by those purporting a violent extremist belief system (p. 6). However, informed by theories of persuasion and communication science, Braddock & Horgan (2016) operationalise counter‐narratives as “narratives comprised of content that challenges the themes intrinsic to other narratives” (p. 386). In this way, the counter‐narrative is defined, by structure and content, as a less direct form of counter‐arguing. However, Braddock et al. also define the counter‐narrative as a tool to “persuade those at risk for radicalization” (2016, p. 387). This view is shared by McDowell‐Smith, Speckhard, and Yayla (2017) who claim that counter‐narratives should intend to persuade audiences by increasing the narrativity (i.e., story‐like quality, see Somers, 1994, p. 616) and emotionality of *their* narrative, trumping that of the propagandist or narrator.

As with any novel concept, these perspective are somewhat ambiguous. Primarily, it is unclear if the counter‐narrative *is* a narrative, or if it is simply a set of techniques intended to challenge a dominant narrative, for the purpose of reconstructing it. Ultimately, it is unclear whether, theoretically, it is the *narrative*, the *countering*, or *both* that are intended to serve as the active ingredient(s) in a counter‐narrative intervention.

Salient to the definitions provided above is the de‐legitimisation of violent means (purported as instrumental in the violent extremist narrative) in order to reduce the likelihood of an individual becoming radicalised; the counter‐narrative can, therefore, defined by its communication goals (Goodall, 2010). Bringing together these components, the counter‐narrative is operationalised, in this review, as an intervention that challenges the rationalisation(s) of violence purported in a dominant narrative which will, in turn, reconstruct the story. Importantly, the definition offered here does not posit that a counter‐narrative must, itself, *be* a narrative. Beyond story‐telling, in the context of violent radicalisation, a counter‐narrative should help individuals to more deeply consider the validity of certain arguments, the rationality of hatred and the legitimacy of violent action.

### 2.3 How the intervention might work

In terms of the mechanics of this idea, there have been a number of proposals for how the intervention may work. These have come from both researcher and practitioner spheres. In 2013, The Quilliam Foundation published a practical guide (Hussain et al., 2014) to countering violent extremism online and advised governments to create counter‐extremist content that challenges the various political or theological *arguments* put forth in dominant narratives. In terms of evidence, this approach (i.e., the technique of contradicting, or directly countering, an argument or narrative with the intention of refuting it, see Wheeler, Briñol, & Hermann, 2007, p. 151) has seen mixed empirical success. In early research on stereotyping, Brock (1967) found that pointing out inconsistencies (or discrepancies) in a persuasive appeal reduced belief change in certain cases through more sophisticated information processing. However, Taber and Lodge (2006) found that participants who were offered pro and con arguments for topical issues such as affirmative action and gun control uncritically accepted arguments which supported their own, baseline attitudes and counter‐argued ones to the contrary. In other words, for participants with existing, baseline attitudes, the approach worked differently, and was not effective. It has been suggested that this occurs when a strategy focuses exclusively on the content of the argument, ignoring, according to Schwarz, Sanna, Skurnik and Yoon (2007), “the metacognitive experiences that are part and parcel of the reasoning process” (p. 128).

For this reason, some have highlighted the impracticality of crafting counter‐narratives that are both initiated, and shaped, by an adversary. A counter‐narrative comprised of counter‐arguments inevitably ends up becoming an “information contest” (Reed, Ingram, & Whittaker, 2017, p. 44). Irrespective of the correctness of the information (which introduces moral ambiguity), simply correcting misinformation is not likely to “dislodge the feeling” (Kahneman and Frederick, 2005, p. 278) that what one believes (or what one has learned) is correct. The same has been suggested of contradictory evidence to dispel erroneous beliefs. Attempts to do so often increase later acceptance of the prior beliefs, as observed in Allport and Lepkin's pioneering research on rumours (1945).

Subsequently, others in the field have suggested that logical arguments will pale in comparison to *emotionally evocative* counter‐narratives. In fact, such strategies have been described as “more important than evidence” (Radicalisation Awareness Network, 2015, p. 6). In line with these guidelines, attempts have been made to create and edit ISIS defector videos (i.e., first‐person critique of the organisation and its tenets) to their most “damaging, denouncing and derisive content” (McDowell‐Smith et al., 2017, p. 55). The intention here is to add to the speaker's emotionality and, presumably, the target's capacity for identification (see Cohen, 2001), reducing the appeal of dominant, pro‐ISIS narratives. In terms of theory and evidence, much understanding of persuasive processes (and narrative persuasion, in particular) have been informed by resistance‐based theories such as “Reactance Theory” (Brehm, 1966), “Cognitive Dissonance Theory” (Aronson, Turner, & Carlsmith, 1963; Festinger, 1957), and dual process models of cognition such as the “Elaboration Likelihood Model” (ELM; Petty & Cacioppo, 1986; Petty & Wegener, 1999), and Green and Brock's (2002) “Transportation‐Imaginary Model”. These models view narrative persuasion as a process occurring through a peripheral, rather than central route. The latter suppresses resistance and counter‐arguing, allowing for a narrative to successfully persuade. However, the application of these theories to countering existing, dominant narratives through the mechanics described above has not been extensively examined.

In 2008, the United Nations published a report of different approaches to countering violent extremist content online. Among the strategies cited were those that implemented “alternative views” (p. 16), or an *alternative account of events*. Conceptually different to alternative narratives (which, by definition, do not directly undercut the logic of violent extremism), this approach involves presenting the same story from a different perspective. Informed by the ELM, this approach has seen some success in the context of counter‐stereotypical information, and early research on stereotyping. According to the ELM, individuals process information through two channels; the first, also known as “system one”, is quick, intuitive and requires very little “cognitive effort” (Dhami & Thomson, 2012, p. 219); the second, also referred to as “analysis” (Kahneman, 2003; Lamond & Thompson, 2000), or “reflective judgement” (Kitchener & King, 1990) is a slower, more careful and consistent form of information processing (Hamm, 1988). Vasiljevic and Crisp (2013) found that exposing participants to contradictory information about another social group encouraged more systematic, “system two” processing of information, resulting in lower hostility. Similarly, Power et al. (1996) found that introducing counter‐stereotypical information about African‐Americans to Caucasian Americans reduced their attribution of blame towards a target.

Finally, the creation of one's *own* counter‐arguments could be applied to the design of counter‐narratives. This is well‐documented in “Inoculation Theory”, which posits that exposing individuals to weakened arguments can inoculate (i.e., protect) them against stronger arguments of the same nature (McGuire, 1961a, 1961b). The theory follows the same rationale as viral inoculation, whereby a weaker form of a virus is introduced to the body to encourage the creation of antibodies, reducing susceptibility to an active viral infection. However, instead of developing antibodies, the individual develops counter‐arguments to reduce their susceptibility to persuasion. In a meta‐analysis of 54 cases, inoculation was found to be an effective form of creating resistance to persuasive messages when compared to matched controls (Banas & Rains, 2010). Successful attempts have also been made to experimentally manipulate inoculation techniques in the context of violent radicalisation‐prevention (Braddock, 2019).

However, despite suggestions and theoretical insights from proximal research areas, specific theoretical frameworks to inform the design of counter‐narratives have yet to be developed.

### 2.4 Why it is important to do the review

In 2017, the United Nations Security Council adopted Resolution 2354. Tasked with preventing violent radicalisation through narratives, this Resolution seeks to achieve multiple strategic communication objectives, amongst which includes the development of effective counter‐narratives. Following this, the European Commission appointed the International Centre for Counter‐Terrorism (ICCT) to identify and report on the state of knowledge in regards to this elusive strategy. Despite an overwhelming volume of guides, reports and individual studies on the topic, however, Reed and colleagues (2017) described the counter‐narrative as conceptually “underdeveloped” and lacking a “thorough grounding in empirical research” (p. 8). As such, calls have been made for more stringent methodological designs in terms of counter‐narrative evaluation, such as baseline measures and control groups. Notes have also been made on the need for a stronger theoretical focus in order to develop a more thorough understanding of the behavioural and attitudinal bases of messaging efforts. Ultimately, there appears to be a “glaring gap” (Reed, 2018, p. 1) in counter‐narrative research, despite its stature in policy.

While there does not yet exist a large‐scale synthesis of counter‐narrative interventions in the context of violent radicalisation, syntheses of *similar* approaches have been conducted in other research fields. For example, Stice and Shaw (2004) provided meta‐analytic evidence on the use of a proximal approach called dissonance‐based interventions (DBI) which encourage individuals to adopt a way of thinking that contradicts their current way of thinking (e.g., challenging social constructions of “beauty” or “thinness”). Furthermore, Chan, Jones, Hall‐Jamieson and Albarracin (2017) provided meta‐analytic evidence on the factors underlying effective counter‐arguing or “debunking” of misinformation (e.g., conspiracy theories or “fake news”). Although efforts have been made to synthesise current governmental and nongovernmental strategies to counter violent extremist narratives (Briggs et al., 2013) and provide a “horizon scan” (Ferguson, 2016) of the research landscape, to date, there has been no synthesis of the effectiveness of counter‐narrative interventions for the prevention of violent radicalisation (Schmid, 2014). This review seeks to address this.

The review will contribute to existing theory and evidence on counter‐narrative interventions, allowing researchers and practitioners to better understand message construction as well as the psychological fulcra for change‐targeted. Most importantly, the review will offer evidence on the effectiveness of the approach in reducing outcomes related to violent radicalisation. In doing so, the review may help those tasked with preventing violent radicalisation to effectively counter harmful, violence‐promoting messages masquerading as innocuous stories.

## 3 OBJECTIVES

The objective of this review was to provide a synthesis of the effectiveness of counter‐narratives in reducing the risk of violent radicalisation. The review question that guided this research was:

What is the impact of targeted counter‐narrative interventions on violent radicalisation (primary outcomes) and/or risk factors for violent radicalisation (secondary outcomes)?

## 4 METHODS

### 4.1 Title registration and review protocol

This review followed an explicit protocol with methodological guidance provided by the Campbell Collaboration. The title was registered in The Campbell Collaboration Library of Systematic Reviews in September 2017. The protocol was published in September 2018 (Carthy, Doody, O'Hora and Sarma, 2018).

### 4.2 Criteria for considering studies for this review

See Appendix C for coding categories according to the inclusion and exclusion criteria.

#### 4.2.1 Types of studies

In order to confidently determine the effectiveness of the intervention, studies adopting an experimental or quasiexperimental design where at least one of the independent variables involved comparing a counter‐narrative to a control(or comparison exposure) were included in the review.

Eligible study designs included:

1. Randomised control trials (RCT) whereby participants are randomly assigned to experimental or control conditions (e.g., two‐group between‐subjects design).
2. Factorial designs, with more than one independent variable (e.g., pre‐post as a within‐subjects variable, and exposure (e.g., present/absent) as a between‐subjects variable).
3. Quasiexperimental studies such as (nonrandomised) treatment versus control designs with/or pre‐/posttest designs (i.e., base‐line measure(s) of outcomes before and after the intervention)

Consistent with Campbell Collaboration policies and procedures, studies using experimental and quasiexperimental designs were synthesised separately.

#### 4.2.2 Types of participants

In order to operationalise the intervention as a counter‐narrative, the participants must have been exposed to an existing (or “dominant”) narrative before or after exposure to the narrative intended to counter it. This was the only criterion applied to participants or settings in the review. This criterion, in terms of the nature of the intervention, is discussed in more detail in below.

#### 4.2.3 Types of interventions

Eligible interventions included those that implemented a strategy to challenge (or “counter”) a dominant narrative which, through a process of violent radicalisation, could be said to promote violent extremism or terrorism if not otherwise offset. These dominant narratives did not need to necessarily *incite* violence; however, they did need to promote a belief system in which the success or survival of the participant's in‐group was portrayed as inseparable from hostile action against an out‐group (i.e., a violent extremist belief system, see Section 2.1).

##### 4.2.3.1 Counter‐narrative

As the review was interested in observing the effects of a *counter*‐narrative (rather than a narrative), studies that exposed participants to a narrative that did not challenge a pre‐existing or experimentally introduced (pre‐ or postintervention, as discussed further in the next section) dominant narrative were excluded. For example, many studies were identified in which participants were exposed to a persuasive news article or video clip before their postexposure attitudes were measured. However, in order to be considered a counter‐narrative, the participants' pre‐exposure attitudes towards the persuasive topic must have been targeted or countered. It was not sufficient that a study exposed a sample to a persuasive (even benevolent) appeal. The narrative must be actively challenging themes within a dominant narrative. Davenport (2013) exposed introductory psychology students to a manipulated news clip about a terrorist attack before measuring their policy preferences and anxiety. However, the exposure material was not designed to challenge a dominant narrative, nor was a dominant narrative ever gauged or experimentally introduced within the sample. For this reason, the study can only be characterised as measuring the effects of exposure to a narrative, rather than a counter‐narrative.

However, this approach is, in itself, quite abstract: does a narrative which is not challenging an existing narrative exist? Narratives are characteristically persuasive (Braddock et al., 2016) and persuasion is characteristically counter‐attitudinal (Cacioppo, Kao, Petty, & Rodriguez, 1986). However, it is not within the scope of this review to scrutinise study samples to determine the extent to which the narrative used in the study can be classified as a counter‐narrative. Instead, guided by the study authors, if the study provided evidence (e.g., pilot‐testing or pretest scores) or sufficient justification (e.g., previous research or content analysis) that the intervention was attempting to counteract an existing narrative identity (i.e., an internalised and evolving social construction) on any given experience, the study was included. Such social constructions may have included the perceived attributes or behaviour of a particular group during events of the past, such as a conflict (e.g., anti‐British in the context of the Northern Irish Troubles) or in society more generally (e.g., perceptions of particular social groups as lazy, dangerous, or inferior).

For example, a number of studies included in this review were conducted in samples with strong, historical narratives that supported or opposed different “sides” (an “ethos of conflict”, see Bar‐Tal, Raviv, Raviv and Dgani‐Hirsh, 2009, p. 94). Two studies were conducted by Alhabash and Wise (2012, 2015) in an American University sample in which participants were exposed to a counter‐narrative which, in the context of the Israeli‐Palestinian conflict, challenged an anti‐Palestinian/pro‐Israeli dominant narrative. In both studies, the dominant narrative was identified in the sample through measuring participants' pretest, implicit attitudes towards either side using the “affective misattribution procedure” (AMP; Payne, Cheng, Govorun, & Stewart, 2005).

Similarly, Cernat (2001) exposed a Romanian sample to pro‐Hungarian narratives which challenged the dominant, anti‐Hungarian narrative of oppression and territorial integrity in the region. As well as conducting a content analysis on local newspapers to identify the dominant narrative, postexposure attitudes in the control group revealed adverse stereotyping of Hungarians compared to Romanians. In other cases, the dominant narrative was experimentally introduced, allowing for more stringent control of the manipulation. For example, after exposing participants to a counter‐narrative, Banas and Richards (2017) exposed American University students to a dominant narrative in the form of a 40‐min film clip of “Loose change: Final cut” (Avery, 2007); an antigovernment, conspiracy‐theory film detailing the supposed role of the United States government in 9/11. Evidence of the various, dominant narratives identified in each study in the review is provided in Table A1: Data Extraction (Appendix A, see “Counter‐narrative” (CN) and “Dominant narrative” (DN) sections).

##### 4.2.3.2 Temporal ordering of the intervention

It is important to highlight that no exclusion criteria were applied to the order of the intervention. Counter‐narrative interventions introduced before exposure to a dominant narrative (preventative interventions), as well as those introduced after exposure to a dominant narrative (therapeutic interventions) were both included in the review. In the former, the intervention would reduce the dominant narrative's effectiveness, acting as a protective factor. In the latter, the dominant narrative would precede the intervention, acting as a treatment. Both could be said to reduce propensity towards violent radicalisation.

#### 4.2.4 Types of outcome measures

There is a clear bias in counter‐narrative evaluations towards measuring clicks, views, “hits”, frequency and content of Tweets, comments or hashtags, and follower‐count, to mention a few (see Radicalisation Awareness Network, 2015, p. 12). While these offer insight into, for example, intervention feasibility, they do not provide an empirical basis that can determine effectiveness. Included studies, therefore, needed to investigate the connection between exposure to a counter‐narrative and propensity towards violent extremism or terrorism (through a process of violent radicalisation) by measuring at least one empirical, primary or secondary outcome. Reliability was assessed according to Cronbach's alpha (Cronbach, 1951).

##### 4.2.4.1 Primary outcomes

Primary outcomes included those in which participants indicated intent to act violently, also referred to as “harmful end objectives” (Powis, Randhawa‐Horne, & Bishopp, 2019, p. 15) TIME or “expressed intent to act violently” (Pressman & Flockton, 2014). Although certain risk assessment tools were consulted for identifying primary outcomes, they did not directly inform the identification of primary outcomes for this review.^3^

##### 4.2.4.2 Secondary outcomes

The process of identifying secondary outcomes involved the categorisation and subcategorisation of relevant outcomes under empirically supported risk factors for the adoption of an extremist or radical belief system (i.e., an “overall risk factor” for violent radicalisation). In most cases, the measured outcome(s) did not share the same wording as the overall risk factor(s) in the cited literature. The process of conceptually mapping the measured outcome(s) onto identified risk factors (or subcategory risk factors) in the literature is detailed in Table A7: Study Outcome(s) and Associated Risk Factors (Appendix A).

What follows is the empirical basis, as well as a brief description, of the two main risk factors used in this review.^4^

###### 4.2.4.2.1 Perceived group threat

The perceived need to defend against threats is an empirically supported risk factor for violent radicalisation. In their meta‐analysis of 95 samples, and five types of threat, Riek et al. (2006) illustrated how different types of perceived threat displayed significant relationship(s) with attitudes towards an out‐group or adversary. For example, the perception of threatened group interests or “symbolic threat” (“Islamic and non‐Islamic people in the Netherlands have different family values”) has been found to predict participants' perceived illegitimacy of authorities, as well as their in‐group superiority, both of which are empirically supported components of a radical belief system (Doosje, Loseman, & van den Bos, 2013; Doosje, van den Bos, Loseman, Feddes, & Mann, 2012; Saucier, Akers, Shen‐Miller, Kneževié, & Stankov, 2009; van Bergen, Feddes, Doosje, & Pels, 2015).

“Realistic threat”, or the perception of physical threat to one's safety or existence (e.g., “non‐Islamic Dutch people have too many positions of power and responsibility in this country”), has been found to predict contact intentions in the form of “perceived distance” towards an adversarial group (Doosje, Loseman, & van den Bos, 2013). In the context of violent extremism in conflict settings, lower intentions for intergroup contact have been shown to increase intentions for violent political participation (“I support [my group's] decisions to use violence throughout the conflict”) (McKeown & Taylor, 2017, p. 237). Furthermore, the “need to defend against threats” is included as an engagement risk factor in the Extremism Risk Guidelines (ERG22+) (Powis et al., 2019, p. 15). In this review, 14 studies were identified as measuring outcomes categorized under “perceived group threat”. These included measured of both symbolic and realistic threat.

###### 4.2.4.2.2 In‐group favouritism/out‐group hostility

The second, broad risk factor was in‐group favouritism and/or out‐group hostility. Working in tandem with in‐group superiority, the perception that certain out‐groups are inferior to the in‐group is an important component of a radical belief system (Doosje et al., 2013; Loza, 2007). In fact, this dynamic (in‐group vs. out‐group) is a defining characteristic of violent extremism in general (Berger, 2017, 2018). The risk factor can be further subcategorised into two cognitive concepts related to violent action; explicit and implicit bias. For example, Kahn and Davies (2011, p. 574) found that manipulating implicit bias (i.e., rendering an out‐group more “stereotypical”) lowered participants' threshold for violence against an out‐group. In fact, in policing, fatal shootings of unarmed civilians have been described as manifestations of these subconscious, implicit biases (Spencer, Charbonneau, & Glaser, 2016, p. 50). Explicit and implicit bias have been found to manifest in the form of hostility towards an out‐group (Reeve, 2019) and “parochial altruism” (the justification of violent action at the risk of harming oneself; see Abou‐Abdallah, Kashima, & Harb. 2016) has been described as a culmination of both in‐group favouritism and out‐group hostility (Abbink, Brandts, Herrmann, & Orzen, 2012). Finally, variations of in‐group favouritism and/or out‐group hostility feature as attitudinal risk factors in the Extremism Risk Guidelines (ERG22 + ) (Powis et al., 2019, p. 15), as well as the Violent Extremism Risk Assessment 2 (VERA version 2) (Pressman et al., 2014).

Studies that did not report proximal outcomes, regardless of their use of an operationally defined counter‐narrative, were excluded from the review. Some studies reported extraneous outcomes (e.g., blood pressure, self‐esteem etc.) as well as relevant ones; in these cases, only data for proximal outcomes were included in the synthesis.

#### 4.2.5 Duration of follow‐up

Studies reporting follow‐ups of any duration were eligible for inclusion.

#### 4.2.6 Types of settings

There were no geographic or setting‐based limitations in the exclusion criteria. Research conducted in any country or setting was eligible if all other inclusion criteria (e.g., published in English) were met.

### 4.3 Search methods for identification of studies

Potentially relevant literature was identified through a five‐stage search strategy, which comprised:

Stage 1: Scoping exercise.
Stage 2: Targeted keyword searches on a list of relevant databases.
Stage 3: Hand searches of several research and professional agencies' outputs and publications.
Stage 4: Reviewing of reference lists of conceptual papers and/or books on the topic of counter‐narratives for countering violent extremism.
Stage 5: Contacting experts in the area.

The first three stages were conducted simultaneously at the start of the review process (August–September 2016). The final two stages were conducted once duplicates were removed from Stages 1 and 2 (October 2016). Stages 2–3, and 5 were replicated in May 2019 due to the surge in the publication of relevant literature between 2016 and 2019 (see Figure B1 (Appendix B) for review timeline).

#### 4.3.1 Scoping exercise

In conjunction with a specialist librarian at the National University of Ireland Galway, a comprehensive list of search terms was developed. The strategy for searching for relevant literature was based on three initial “concepts”:

- Concept 1: The intervention (“counter‐narrative”)
- Concept 2: The research area (“counter‐terrorism”)
- Concept 3: The problem (“violent extremist narrative”)

The use of Concept 2 allowed for the development of search terms for Concepts 1 and 3. Using the research area as an initial search, frequently occurring terms within papers relating to “counter‐narrative” were recorded and then used alongside the search terms in Concept 2. For example, Concept 2 AND “counter‐narrative” led to the term “alter‐messaging”. The authors then used Concept 2 AND “counter‐narrative” AND “alter‐messaging” to manually search for alternative terms within the search results. This process was extensively repeated until the authors felt saturation was reached. Given that the counter‐narrative is a relatively novel term in the radicalisation literature, this process allowed for the identification of far more literature than if several synonyms had not been explored. See Table A2 (Appendix A) for a full list of the search terms used.

#### 4.3.2 Targeted keyword search

Detailed, electronic searches were then conducted on a number of literature databases (see Table A3, Appendix A). As noted by Silke (2001), the beginning of the 21st century marked a turning point in the use of quantitative methodologies in terrorism research. For this reason, studies published before the year 2000 were excluded from the remaining four stages of the search strategy.

#### 4.3.3 Professional agencies

Following the targeted keyword search, the authors hand‐searched output from research and professional agencies in the area of counter‐terrorism (see Table A4, Appendix A).

#### 4.3.4 Hand‐searching of reference lists

Once duplicates were removed from the literature identified in Stages 2 and 3 of the search strategy, a hand search was conducted on the reference lists of these papers as well as conceptual papers/books on the topic of counter‐narratives (see Table A6, Appendix A).

#### 4.3.5 Contacting experts

Finally, a number of experts (see Table A5, Appendix A) in the field of violent radicalisation and narrative persuasion were contacted for relevant literature (published or unpublished) which matched the selection criteria. One colleague recommended a serious of published bibliographies (Tinnes, 2013a, 2013b, 2014a, 2014b, 2014c, 2014d, 2015a, 2015b, 2016a, 2016b, 2016c, 2016d, 2017) and these were included in Stage 3 of the search strategy. For expert consultation in the second search, the authors contacted the UK Home Office and Public Safety Canada.

### 4.4 Data collection and analysis

#### 4.4.1 Selection of studies

Following these search stages, potential titles and abstracts were imported into Endnote (a bibliographic reference software tool). Once duplicates were removed, the final two stages of the search strategy were conducted and any newly identified references (i.e., from agencies, experts or books) were also imported into Endnote. A second duplicate check was conducted once the literature from all stages were combined.

See Appendix C for coding categories according to inclusion and exclusion criteria. All identified literature underwent a three‐stage screening process:

1) The titles of all literature were screened according to the exclusion criteria and excluded accordingly.
2) The abstracts of the included literature were screened, again, according to the exclusion criteria and excluded accordingly.
3) Finally, the full texts of the remaining included studies were screened according to the *inclusion* criteria, producing the final list of studies to be included in the review.

Potentially eligible studies were then retrieved in full text, and the final selection of included studies was made. Once the final studies were identified, two reviewers independently repeated the three stages of the screening process. The largest source of disparity between reviewers was the identification of the dominant narrative (i.e., was the counter‐narrative intervention challenging an existing, dominant narrative and, if so, was there sufficient evidence of a dominant narrative?) and the study design. For example, there were many studies which did not meet a specific design criterion (see Al‐Rawi, 2013; Frennett & Dow, 2015), and arguments could be made for both their inclusion and exclusions. Any disparities between reviewers were resolved by discussion and consensus, before the final studies were decided.

#### 4.4.2 Data extraction and management

Two reviewers (S.C. and K.C.) double‐coded all included studies, using a piloted codebook (see Appendix C [coding schemes] and Table A1, Appendix A). Again, all coding disagreements were resolved via discussion and consensus. The primary categories for coding were as follows: participant demographics and characteristics (e.g., sample size, age, gender ratio, nationality, and intervention setting); the dominant narrative, as well as the method of determining the dominant narrative (e.g., pilot testing, comparison group, previous research etc.); the counter‐narrative and techniques used (e.g., counter‐stereotypical exemplars, narrative transformation, persuasion); study design, outcome(s) construct(s) and, finally, descriptive statistics and overall effectiveness.

#### 4.4.3 Assessment of risk of bias in included studies

Risk of bias was conducted according to the Cochrane “Effective Practice and Organisation of Care” (EPOC) review group data collection checklist. See Table D1 (Appendix D). Randomised studies were independently coded by two reviewers (S.C. and K.C.) on the following domains:

1. Potential for selection bias/confounding due to nonrandom assignment or sequence generation, inadequate allocation concealment, or important baseline differences in outcomes and/or characteristics
2. Potential for detection bias due to participant knowledge of intervention and control conditions.
3. Potential for attrition bias due to missing outcome data.
4. Potential for performance bias due to systematic differences in the care provided to participants (i.e., contamination).
5. Potential for reporting bias due to selective outcome and analysis reporting.

These domains were further broken down into specific questions. For each of these questions, the study was coded as “low” risk of bias if the issue was addressed, “high” risk of bias if the issue was not addressed and “unclear” risk if the authors did not make reference to information pertaining to the issue. Cohen's Kappa (κ) was calculated for testing inter‐rater reliability (Cohen, 1960) between both coders' risk of bias assessments, with *p* ^(a)^ as the relative observed agreement among raters, and *p* ^(e)^ as the probability of agreement based on chance (*κ* = (*p* ^(a)^ − *p* ^(e)^)/(1 − *p* ^(e)^)).

For the nonrandomised studies, the primary risk pertained to natural change over time (i.e., maturation) and, as such, all nonrandomised studies were categorised as “high” risk of bias.

#### 4.4.4 Measures of treatment effect

As mentioned, during the data extraction, relevant statistics (such as means, standard deviations and sample sizes across conditions) were extracted from results section(s) of included studies (or, in many cases, directly from the study authors) to calculate effect sizes. These effect sizes were reported as standardised mean differences (SMD). Given the nature of the outcomes (e.g., out‐group bias, perceived threat, social stigma), the SMD were adjusted such that positive values (>0) indicated a *negative* outcome (i.e., greater propensity towards violent radicalisation).

#### 4.4.5 Unit of analysis issues

Studies containing more than one independent study (with separate samples) were coded as separate studies (e.g., Bruneau, Lane, & Saleem, 2017). Studies based on the same sample were treated as a single study.

In single level analysis, multiple effect sizes from the same study are known to bias the overall results. Therefore, for studies with more than one outcome categorised under a single risk factor, an average effect size across these multiple outcomes was calculated and used to represent each study (see Brewin, Kleiner, Vasterling, & Field, 2007, p. 450). In the case of studies which contained more than one measure of the same subcategory risk factor, a pooled average was created. In the case of subgroup analysis, studies with measures of more than one subcategory risk factor (e.g., studies which measured both symbolic *and* realistic threat, or in‐group favouritism *and* out‐group hostility) were not pooled (this was necessary in one study (Riles, Funk, & Davis, 2018); see Section 5.3.2.1 synthesising *randomised studies*).

#### 4.4.6 Assessment of heterogeneity

Alongside the chi‐square statistic, a qualitative visual analysis of the studies' effects was conducted using a Baujat plot. By observing the amount of variation in treatment effect, this allowed for easy identification of the largest contributors to between‐study heterogeneity.

#### 4.4.7 Publication bias

To assess publication bias, a contour enhanced funnel plot (Palmer, Sutton, Peters and Moreno, 2008) and Baujat plot (as used above) were produced (Baujat, Mahé, Pignon, & Hill, 2002). Egger's regression test was also conducted (Egger, Smith, Schneider, & Minder, 1997) as well as the Begg and Mazumdar (1994) rank correlation test.

#### 4.4.8 Data synthesis

Intervention effects for randomised and nonrandomised studies were synthesised in separate meta‐analyses using RevMan (Review Manager, Version 5.3). Due to the difference in populations from which the data were sampled (as well as some between‐study heterogeneity) a random effects approach was used (as is generally the norm with social science data, see Field, 2005, p. 445). Moderator analysis was conducted using meta‐regression to assess intervention type(s) (i.e., theoretical basis) as potential effect size moderators. This was done using Comprehensive Meta‐Analysis Version (Borenstein, Hedges, Higgins, & Rothstein, 2013). Studies from which effect sizes could not be calculated were discussed narratively alongside the related meta‐analyses.

#### 4.4.9 Dealing with missing data

When studies reported insufficient data to calculate effect sizes, the primary authors were contacted to request the necessary information. In two cases (Ramasubramanian & Oliver, 2007; Garagozov, 2013) the review authors were unable to acquire all necessary data directly from the study authors.

#### 4.4.10 Sensitivity analysis

For analysis that included pooled outcomes, the authors re‐ran the analysis with singular outcomes chosen according to a different selection criteria (i.e., the most reliable). To detect any potential biasing of the meta‐analysis due to multiple studies within the same publication, specific studies were removed, and the analysis re‐run with any differences in overall effect or between‐study heterogeneity noted. Sensitivity analysis was also run on analyses which contained studies posing a particularly high risk of bias

## 5 RESULTS

### 5.1 Description of studies

**Figure 1:**
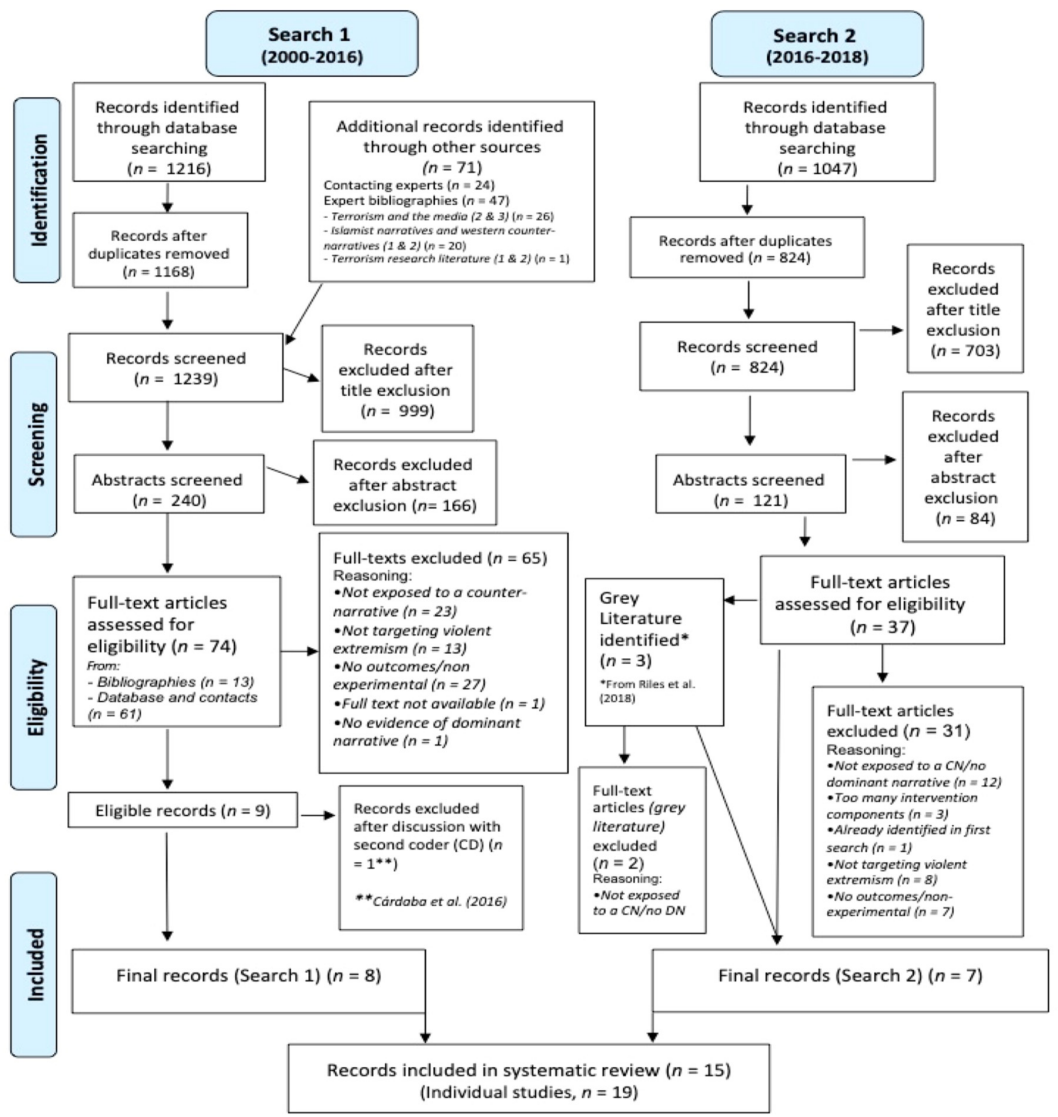
PRISMA (the flow charts have been adapted from Moher et al. (2009). Additional sections have been added to demonstrate the contribution from nondigital sources, as well as the role of the second coder in the screening process) flow chart of searches

As mentioned, this review included two separate searches. The initial search was conducted in August 2016 (date range: 2000–2016) and an updated search was conducted almost three years later (in May 2019) to account for the rise in experimental literature in the area of counter‐narratives (date range: 2016–2018). The two searches are first described independently before the final, included studies are pooled and described collectively for the remainder of the review. See Figure 1 (overleaf) for the flow of studies through the search and screening process, across both searches.

#### 5.1.1 Results of the search

##### 5.1.1.1 Search 1 (2000–2016)

In the initial search (after removing duplicates), 1168 papers were identified through databases, and a further 71 through contacting experts or consulting expert bibliographies (e.g., Tinnes, 2013b, 2014c). After screening all 1,239 titles, 999 papers were excluded; the remaining 240 papers' abstracts were screened according to the exclusion criteria before 73 full texts were assessed based on the inclusion criteria (one full text could not be retrieved).

###### 5.1.1.1.1 Excluded studies

Of these papers, 65 were excluded. The majority were excluded as the study intervention did not meet the operationalised definition of a counter‐narrative (*n* = 23). One study did not show evidence of a dominant narrative; the remainder were excluded as they did not target violent radicalisation (*n* = 13) or employ an experimental design (*n* = 27).

##### 5.1.1.2 Search 2 (2016–2018)

In the second search, only the targeted keyword search was replicated. In the databases which facilitated date parameters, the search was restricted to 2016–2018. However, for smaller databases (e.g., Hedayah, NCJRS) no date restrictions were applied. This resulted in some older, previously undetected, papers.^5^ After deleting duplicates, 824 studies were screened. Both the titles and abstracts were screened according to the exclusion criteria, after which 37 studies were deemed eligible for full text screening. A further three were identified through the full texts' grey literature. Therefore, a total of 40 full texts were screened for eligibility.

###### 5.1.1.2.1 Excluded studies

Of these 40 papers, 33 did not meet the inclusion criteria. Again, the majority of the excluded papers did not describe study intervention(s) which met the operationalised definition of a counter‐narrative (*n* = 14). One study was already identified in the first search. Three studies contained several intervention components and, therefore, the effects of the counter‐narrative could not be distilled. The remainder did not target violent radicalisation (*n* = 8) or did not involved comparing a counter‐narrative to a control or comparison exposure (*n* = 6).

#### 5.1.2 Inter‐rater reliability

Three trained researchers (S. C., C. D. (search 1) and K. C. (search 2)) independently replicated the search screening (title, abstract and full text) for each search. For both searches, Cohen's Kappa (*κ*) was calculated for testing inter‐rater reliability (Cohen, 1960) using the grading scheme as detailed by McHugh (2012) (see Section 4.4.3). For the first search (S. C. and C. D.), two coders obtained *κ* = 0.66, reflecting a moderate level of agreement. The majority of disparities arose due to the difficultly in correctly identifying a dominant narrative and ambiguous experimental designs. For example, S.C. identified Cárdaba et al. (2016) and determined the dominant narrative to be proaggression as evidenced by scores on the Aggressiveness Questionnaire (high scorers could be said to have been exposed to a dominant, proaggression narrative). C. D. disagreed, highlighting that scores on this questionnaire reflect a personality trait, rather than exposure to a specific narrative.

Similar disparities arose due to design. Many studies made claims to “determine the effectiveness” of counter‐narrative interventions (Al‐Rawi, 2013; Frennett & Dow, 2015) but, instead, described interventions in detail without sufficient statistical evidence of effect. Other studies, despite meeting all other inclusion criteria, measured outcomes that represented the “likeability” of a counter‐narrative, rather than any substantial attitude‐change. For example, “likes” and “shares” of counter‐narrative content (see Silverman, Stewart, Birdwell, & Amanullah, 2016).

After calculating agreement, the coders went through each contested paper and differences were solved through discussion and analysis. This process resulted in the removal of one study from the final list (Cárdaba et al., 2016), leaving eight papers from search 1 for inclusion in the review. For the second search (K. C. and S.C.), the coders obtained *κ* = 0.77, reflecting a moderate‐high level of agreement between coders. Again, most disparities arose due to difficulties in determining a dominant narrative. All disagreements were discussed between coders; no studies were added or removed, from the initial search, leaving seven papers from search 2 for inclusion in the review.

#### 5.1.3 Included studies

Nineteen independent studies, reported in 15 papers (eight from search 1, and seven from search 2) met the inclusion criteria.^6^ Three papers (Bruneau et al., 2017; Čehajić‐Clancy & Bilewicz, 2017; Frischlich, Rieger, Morten, & Bente, 2018) reported more than one, independent study within their paper. These studies were coded separately (e.g., Bruneau et al., 2017). The following sections provide a general overview of the 19 included studies.

##### 5.1.3.1 Study characteristics

###### 5.1.3.1.1 Study dissemination

All studies were published in peer‐reviewed journals of varying impact factors. The publication years ranged from 2000 to 2018, with the majority of studies published after 2015.

###### 5.1.3.1.2 Types of studies

####### 5.1.3.1.2.1 Randomised

Twelve studies used randomised control trial designs, with varying types of controls. Bilewicz and Jaworska 2013 used a wait‐list control; Riles et al. (2018) and Banas and Richards (2017) used nature and sushi videos respectively. Gonsalkorale, Allen, Sherman, and Klauer (2010) used a standard (rather than manipulated) version of the implicit association test (IAT, this is explained in more detail in the section on “Delivery”). The remaining RCTs contained more than two conditions and, as such, the most credible control condition was chosen.

As mentioned, for Bruneau et al. (2017) only data pertaining to participants in the “Budrus” (CN) and “Chasing Ice” (no CN/control) narrative conditions are reported. In Cernat (2001), only data pertaining to the “Hungarian Positive” (CN) and “control” (No CN) conditions are reported. In Saleem et al. (2015), only data pertaining to the counter‐stereotypic (CN) and neutral/no CN (a news‐clip about a football rescheduling due to Ramada) conditions are reported. In Ramasubramanian and Oliver (2007) the “counter‐stereotypical African‐American” condition served as the counter‐narrative (CN), while the “stereotypical African‐American” condition was considered to be a credible control.^7^ In Garagozov et al. (2013) data pertaining to all conditions (all of which could be defined as counter‐narratives) are reported, although comparisons are only made between each condition and the designated control.

Finally, in Cohen et al. (2015), data pertaining to the antidemonstration participants across both conditions are included: prodemonstration character rendered virtuous (CN) and antidemonstration character rendered virtuous (control/No CN).

####### 5.1.3.1.2.2 Nonrandomised

The review includes five nonrandomised studies. Three studies used single group pre‐/posttest designs (Čehajić‐Clancy & Bilewicz, 2017; Kendrick & Fullerton, 2004) and, therefore, served as their own control.

Two additional studies (with almost identical design(s) and procedure(s); Alhabash and Wise, 2012, 2015) used a 2 × 2 factorial design (with a within‐subjects factor), but were interpreted as single group pre‐/posttest designs. This was done because neither treatment condition was considered an appropriate control. In other words, while one condition (“Palestinian president”) was considered a counter‐narrative intervention (countering a pre‐existing, dominant narrative in the sample), those in the other condition (“Israeli prime‐minister”) were simply exposed to a different *type* of narrative (one that likely strengthened their existing one). This condition could, therefore, not be considered a suitable control or comparison for those in the counter‐narrative condition, and participants in this condition were not included in the review. The two studies were, therefore, interpreted as nonrandomised, single group pre‐/posttest designs.

Finally, Frischlich et al. (2018) used two interrupted time series designs. No quasiexperimental designs with (nonrandomised) treatment or control/comparator condition(s) were identified.

###### 5.1.3.1.3 Types of settings

The studies represented a range of geographical locations. The region most heavily represented was North America (*n* = 10). Other countries which featured included Azerbaijan (*n* = 1), Bosnia and Herzegovina (*n* = 2), Germany (*n* = 2), Israel (*n* = 2), Romania (*n* = 1), and the UK (*n* = 1). The majority of studies were conducted in University (*n* = 12) or high school (*n* = 2) settings with corresponding samples (four reported recruiting their sample(s) using MTurk). The remainder did not specify their setting, simply reporting that participants were recruited from the local area (Čehajić‐Clancy & Bilewicz, 2017; Frischlich et al., 2018; Garagozov, 2013).

###### 5.1.3.1.4 Dominant narratives

The dominant narratives, in most cases, comprised of hostile social constructions of an adversary or “out‐group”. In eight studies, the dominant narratives were contextualised in terms of international conflicts. For example, in the context of the Israeli‐Palestinian conflict, six studies attempted to counter an anti‐Palestinian narrative which either placed excessive blame on the Palestinian side for the events of the conflict (Alhabash & Wise, 2012, 2015; Bruneau et al., 2017) or supported civil rights restrictions on Palestinians (Cohen et al., 2015).^8^ In some studies, the dominant narratives were contextualised in conflicts of the past. In an Israelihigh school sample, Bilewicz & Jaworska (2013) countered an anti‐Polish narrative. This narrative was based on perceived deeds perpetrated against the high‐schoolers Jewish ancestors' during the Holocaust. Similarly, in the context of the Armeno‐Azerbaijani Nagorno‐Karabakh conflict, Gargagozov (2013) attempted to counter an anti‐Armenian narrative in their Azerbaijani sample.

In the remaining studies, the dominant narratives simply presented as prejudicial leanings towards other ethnic groups (Čehajić‐Clancy & Bilewicz, 2017). For example, an anti‐Hungarian narrative in a Romanian sample (Cernat, 2001), anti‐African American in a Caucasian sample (Ramasubramanian & Oliver, 2007; Gonsalkorale, Allen, Sherman, & Klauer, 2010), and anti‐Muslim in an American student sample (Riles et al., 2018; Saleem et al., 2015). Four studies countered antigovernment narratives (Banas & Richards, 2017; Kendrick & Fullerton, 2004), two of which were specifically labelled as “right‐wing” and “Islamist” extremism (Frischlich et al., 2018).

In the majority of studies, the dominant narratives were determined through pretest or baseline scores. For example, before exposure, Alhabash and Wise (2012, 2015), measured participants baseline ratings of Israelis and Palestinians and observed more negative ratings towards Palestinians in terms of their responsibility for violence, desire for peace and capacity for democracy (compared to Israelis). This provided evidence of an existing, anti‐Palestinian narrative among the sample. Two studies included pilots, and these provided evidence of dominant narratives within the samples (Ramasubramanian & Oliver, 2007; Riles et al., 2018).

Four studies used comparison groups which allowed the review authors to identify if, compared to a neutral group (supposedly exposed to a specific narrative), the control group reported similar scores on different outcome measures. For example, in Bruneau et al. (2017), participants who watched a video depicting Palestinians as violent shared similar, anti‐Palestinian views to those who watched a global warming video. In other words, the control group were as “anti‐Palestinian” as those who were provided with “evidence” of Palestinians being violent. The narrative that the Palestinian side of the conflict is violent was, therefore, likely present in the sample already.

Finally, prior to exposure to a counter‐narrative, three studies introduced the dominant narrative(s) experimentally (Banas & Richards, 2017; Frischlich et al., 2018).

###### 5.1.3.1.5 Types of counter‐narrative intervention(s)

####### 5.1.3.1.5.1 Delivery

Ten studies delivered their counter‐narrative in video format. These included commercials (Kendrick & Fullerton, 2004), movie or television clips (Riles et al., 2018; Saleem et al., 2015), films or film trailers (Bruneau et al., 2017; Čehajić‐Clancy & Bilewicz, 2017) and documentary‐style testimonials (Frischlich et al., 2018). Six studies delivered their counter‐narratives in written format, in the form of a newspaper article (Ramasubramanian & Oliver, 2007), historical account (Cernat 2001; Bilewicz & Jaworska, 2013; Garagozov, 2013), fictional story (Cohen et al., 2015) or a simple message containing counter‐arguments (Banas & Richards, 2017). Two studies used a video game (Alhabash & Wise, 2012, 2015) and, finally, Gonsalkorale et al. (2010) used a manipulated version of the implicit association test (IAT). The IAT is a psychological measure that detects the strength of associations between mental representations. It is generally used to measure implicit prejudice or stereotypes, see Greenwald et al. (2003).

####### 5.1.3.1.5.2 Theory and techniques

See Table 1.

**Table 1:** Theory and techniques

Counter‐narrative technique(s)	Number of studies	Number of effect sizes^a^	% of studies
*Counter‐stereotypical exemplars*	9	15	48
**Stereotype Content Model** (Fiske, Cuddy, Glick, & Xu, 2002)	1	1	
Ramasubramanian and Oliver (2007)			
**Needs‐Based Model of Reconciliation** (Nadler & Shnabel, 2006)	1	2	
Bilewicz and Jaworska, (2013)			
**Priming Theory** (Berkowitz, 1984)	1	2	
Riles et al. (2018)			
**Social cognitive theory/schemas** (Bandura, 1977)	1	2	
Saleem et al. (2015)			
** Intergroup Contact Theory** (Pettigrew & Tropp, 2006)	2	4	
Čehajić‐Clancy & Bilewicz, (2017)^b^; Gonsalkorale et al. (2010)			
**Quadruple Process Model** (Sherman et al., 2008)	1	1	
Gonsalkorale et al. (2010)			
** Not specified**			
Cernat (2001); Kendrick and Fullerton (2004)	2	3	
*Persuasion*	5	7	26
**ELM** (Petty & Cacioppo, 1986)/**Transportation‐Imaginary Model** (Green & Brock, 2002)	*5*	*7*	
Alhabash and Wise (2012, 2015); Cohen et al. (2015); Frischlich et al. (2018)^c^			
*Inoculation theory* (McGuire & Papageorgis, 1962)	1	1	5
Banas and Richards (2017)			
*Alternative account*	4	6	21
**Progressive Narrative Transportation** (Garagozov, 2012)	*1*	*0*	
Garagozov et al. (2013)			
**Tripartite Model of Identity** (Hammack, 2008)	*3*	*6*	
Bruneau et al. (2017)			
Total	19	29	100

a Some studies measured more than one outcome variable, and therefore reported more than one effect size.

b These interventions also incorporated contact with the adversarial group.

c Although these interventions largely made use of persuasive techniques, they also used other techniques such as encouraging perspective taking and providing participants with more information/education.

Counter‐stereotypical exemplars. The majority of studies (48%) employed the use of counter‐stereotypical “exemplars” in the form of stereotype challenging, prosocial, or moral exemplars to challenge the dominant narratives in their respective samples. These were informed by various, theoretical frameworks.

Informed by the stereotype content model, Ramasubramanian and Oliver (2007) had participants read a newspaper article in which African Americans were depicted as “gentle” and associated with entrepreneurial success. These counter‐stereotypical exemplars were designed to increase motivation to inhibit prejudice (i.e., wanting to appear nonprejudiced in a public setting), as well as expose participants to additional, counter‐stereotypical information (i.e., “egalitarian beliefs”, p. 626).

Five studies used exemplars depicting the adversarial group being prosocial. These were informed by a number of theoretical frameworks (or none at all). Informed by the “Needs‐Based Model of Reconciliation”, Bilewicz and Jaworska, (2013) demonstrated Polish people helping Jews in World War II; Riles et al. (2018), based on priming theory, depicted Muslims aiding non‐Muslim characters in day‐to‐day community activities. Similarly, informed by “Social Cognitive Theory” and research on schemas, Saleem et al. (2015) depicted Muslims volunteering during Christmas. Finally, although Cernat (2001) and Kendrick and Fullerton (2004) did not explicitly state their theoretical framework, they applied similar techniques. Cernat (2001) simply depicted Hungarians as “positive”, while Kendrick and Fullerton (2004) depicted the happy lives of Muslims living in the United States post 9/11.

Finally, three studies used counter‐stereotypical exemplars rooted in the concept of morality. Čehajić‐Clancy & Bilewicz, (2017) were informed by “Intergroup Contact Theory” and used “moral exemplars” to increase participants' awareness of the “historical, moral variability of the out‐group” (p. 290). These interventions were somewhat eclectic, as they also made use of contact with the adversarial group. Gonsalkorale et al., were also informed by intergroup contact theory, as well as the “Quadruple Process Model”. Using implicit, positive exemplars, participants were shown positive images of out‐group members in an attempt to create novel associations (i.e., associating pleasantness with Black people) to reduce implicit bias.

*Persuasion*. For 26% of the studies, the counter‐narratives used persuasive techniques, all informed dual‐process models of persuasion; the “Elaboration Likelihood Model” and the “Transportation‐Imaginary Model” (see Section 2.3 for brief description). In other words, the counter‐narratives in these studies were designed to induce peripheral‐route persuasion. Alhabash and Wise, 2012, 2015) used role play through the use of a video game to initiate self‐persuasion through the mechanisms of identification and transportation. Cohen et al. (2015) presented participants with a counter‐attitudinal protagonist, while attempting to increase participants' identification with him/her (and initiate narrative persuasion).

Finally, Frischlich et al. (2018) used a variety of techniques, but mainly sought to increase the “narrativity” of the counter‐narrative and induce transportation. However, Frischlich et al. also incorporated elements of perspective‐taking, emotional appeals, and counter‐arguments, rendering it difficult to isolate the specific techniques used.

*Inoculation*. One study, informed by “Inoculation Theory” (Banas & Richards, 2017), attempted to trigger counter‐arguing and perceived threat by warning participants of a forthcoming persuasive appeal (“explicit forewarning”), before offering counter‐arguments (“refutational pre‐emption”).

*Alternative accounts*. Four studies employed alternative accounts in their counter‐narratives, encouraging the participant to engage with the dominant narrative before offering another course of action or point of view. This approach was informed by different theoretical frameworks. Using “Progressive Narrative Transformation”, Garagozov et al. (2013) developed “common narratives” for participants to make sense of the past and create a “shared vision” of the future without intergroup tension. Along a similar vein, Bruneau et al., noted how fallaciously perceiving a side as violent can compromise third‐party sympathy (Vandello, Michniewicz, & Goldschmied, 2011). As such, informed by “Narrative Identity Theory”, they offered participants an alternative account of the same events in the dominant narrative. By challenging the “entrenched” dominant narrative that the Palestinian resistance is a violent one, Bruneau et al. (2017, p. 747) attempted to increase participants' favourability towards the out‐group.

###### 5.1.3.1.6 Types of outcomes

As mentioned, the process of recognising which outcomes to include in the synthesis involved the mapping of outcomes onto evidence‐based components, or determinants, of a radical belief system (see Table A7, Appendix A). These outcomes (as well as their respective studies and effect sizes) are summarised in Table 2, below.

**Table 2:** Primary and secondary outcomes

	Number of studies	Number of effect sizes
*Primary outcomes*	**3**	**3**
For example, “support for military action in Muslim countries” (Saleem et al., 2015); “agreement with right wing extremist statements” (Frischlich et al., 2018)		
*Secondary outcomes*		
Perceived threat		
Symbolic threat: *the perception of threatened group interests*.	7	7
For example, national attitudes (Alhabash and Wise, 2012); “perceived similarity to the self” (Bilewicz & Jaworska, 2013); “belief in reconciliation” (Čehajić‐Clancy & Bilewicz, 2017)		
Realistic threat: *the perception of threat to one's safety or existence*	*9*	*8*
For example, attitudes towards demonstrations (Cohen et al., 2015); social distance (Riles et al., 2018); “perceptions of Muslims as aggressive” (Saleem et al., 2015)		
*In‐group favouritism/out‐group hostility*		
Explicit bias: *perception that certain out‐groups are inferior*	*9*	*8*
For example, “negative evaluations” (Cernat, 2001); “feeling thermometer” (Bilewicz & Jaworska, 2013), intergroup anxiety (Čehajić‐Clancy & Bilewicz, 2017)		
Implicit bias: *relying on stereotypical information in relation to an out‐group*.	*4*	*3*
For example, implicit association test (Gonsalkorale et al., 2010); affective misattribution procedure (Alhabash & Wise, 2012; 2015)		
Total number of effect sizes		29

####### 5.1.3.1.6.1 Primary outcomes

Primary outcomes included those in which participants indicated intent to act violently. Saleem et al. (2015) measured participants support for military action in Muslim countries. Participants rated their agreement with 9 statements (e.g., “I would support the use of U.S. military to reduce the influence of Islam on other countries”) on a 5‐point Likert scale (Henry, Sidanius, Levin, & Pratto, 2005). Frischlich et al. (2018) measured agreement with violent extremist propaganda. Participants indicated their agreement with 10 extremist statements (both Right‐Wing and Islamist) on a 7‐point scale (e.g., “the caliphate/the national resistance shows the Muslims/the Germans the solution to their problems”, pp. 5–6).

####### 5.1.3.1.6.2 Secondary outcomes

Secondary outcomes included those identified as “risk factors” for an extremist or radical belief system (i.e., an “overall risk factor” for violent radicalisation). As mentioned, from the included studies, perceived group threat and in‐group favouritism/out‐group hostility emerged as the main risk factors (see Section 4.2.4). Although the majority of measures were not validated, most provided indicators of reliability using Cronbach's Alpha; throughout this review, *α* > .70 is interpreted as “acceptable” (see Abraham & Barker, 2015; Taber, 2018).

######## 5.1.3.1.6.2.1 Perceived group threat

Fifteen studies in this review measured components of perceived threat, both symbolic and realistic. In the “symbolic threat” subcategory, Bilewicz & Jaworska, (2013) measured Israeli participants' “perceived similarity” to Polish people with items such as “How much do you share common interests with young Poles?” (*α* = .81). Alhabash and Wise (2012) measured American participants' agreement with Palestinian “national attitudes” with items such as “[Palestinians] want peace” and “[Palestine] is democratic” (*α* = .81). Riles et al. (2018) used a validated measure (*α* = .89) of “social stigma” (Smith, 2012, p. 530).

Outcomes sub‐categorised under “realistic threat” included participants' perceptions of the out‐group as dangerous (Saleem et al., 2015, (*α* = .90) or violent (Bruneau et al., 2017; *α* = .87–93); conspiratorial or antigovernment attitudes (Banas & Richards 2017, *α* = .96), attitudes towards minority protests (Cohen et al. 2015, *α* = .83); desire for distance (Riles et al. 2018, *α* = .92), and support of civil restrictions towards an out‐group (Saleem et al. 2015, *α* = .92). Čehajić‐Clancy and Bilewicz, (2017) measured “belief in reconciliation” (“I doubt that we will ever be able to live together in peace”), which had borderline acceptable reliability (*α* = .66–.73). Finally, Alhabash and Wise (2015) and Cernat (2001) measured explicit stereotypes about Palestinians (“dirty”, “lazy”, “untrustworthy”) and Hungarians (“extreme”, “aggressive”) respectively. Kendrick and Fullerton (2004) measured participants towards the U.S. government, as well as its treatment of Muslims. These studies did not provide reliability analysis.

For studies which more than one measure of the same subcategory risk factor (e.g., two outcomes categorised as “symbolic threat”), the effects were pooled (i.e., Kendrick and Fullerton 2004; Saleem et al., 2015). Riles et al. (2018) measured two outcomes categorised as “realistic threat” (“could you see yourself renting a room to a Muslims person?”), and symbolic threat (“most people would think less of a Muslim”) respectively. These measures were not pooled as they represented separate subgroups (see Section 4.4.5). In any analysis containing both outcomes, the results were interpreted with caution due to the study being represented by two, separate outcomes.

######## 5.1.3.1.6.2.2 In‐group favouritism/out‐group hostility

Thirteen studies were identified as measuring components of in‐group favouritism and/or out‐group hostility. These included both explicit and implicit measures. Gonsalkorale et al. (2010) was the only study to measure in‐group favouritism; they used the IAT (see Section 5.1.3 “Delivery” for brief description).

Out‐group hostility was measured using both implicit and self‐report measures. Using a standardised measure of “intergroup anxiety”, Čehajić‐Clancy and Bilewicz, (2017) had participants indicate their trust, confidence, discomfort and so forth, towards the out‐group (see Lolliot et al., 2015, p. 666). The reliability at both time points was poor (*α* = .54– .61). Four studies used a 100‐point “feeling thermometer” (Bilewicz & Jaworska, 2013; Bruneau et al., 2017). Cernat (2001) measured out‐group hostility through “evaluations” whereby participants rated their respective out‐group on traits such as respect, appreciation, hate, disgust, and annoyance (no reliability analysis were provided). Čehajić‐Clancy and Bilewicz, (2017) used a “forgiveness” measure (*α* = .78–.83) adapted from Čehajić‐Clancy, Brown, and Castano (2008; Study 1) in which participants rated their agreement with items such as “my [in‐group] should never forgive [out‐group] for their misdeeds”. Alongside self‐report measures, two studies (Alhabash and Wise, 2012, 2015) measured out‐group hostility using the affective misattribution procedure (AMP). The AMP measures automatic responses based on mistakes or misattributions about the sources the response; it is commonly used to measure prejudice and political behaviour (Payne & Lundberg, 2014).

### 5.2 Risk of bias in included studies

As mentioned, two independent coders (S.C. and K.C.) used the EPOC data collection checklist to assess risk of bias. This checklist allows the coders to rate study designs against nine study criteria, indicating a high, medium or low risk of bias on that particular item. Cohen's Kappa (*κ*) (Cohen, 1960) was calculated for testing inter‐rater reliability on a total of 171 risk of bias items (i.e., 9 items across both randomised and nonrandomised studies), obtaining *κ* = 0.72 (i.e., substantial agreement between coders). Table 3 (overleaf) summarises the risk of bias for the 12 randomised studies included in this review.

**Table 3:** Risk of bias in randomised studies

	Low risk		Unclear risk		High risk	
	*N*	%	*N*	%	*N*	%
Was the allocation sequence adequately generated?	11	92	1	8	0	–
Was the allocation adequately concealed?	6	50	6	50	0	–
Were baseline outcome measurements similar?	3	25	8	67	1	8
Were baseline characteristics similar?	11	92	0	–	1	8
Were incomplete outcome data adequately addressed?	11	92	1	8	0	–
Was knowledge of the allocated interventions adequately prevented during the study?	9	75	1	8	2	17
Was the study adequately protected against contamination?	12	100	0	–	0	–
Was the study free from selective outcome reporting?	11	92	1	8	0	–
Likelihood of other risk(s) of bias?	11	92	0	–	1	8

*Note: n*  = 12.

The majority exhibited low risk of bias for randomisation to conditions (92%), treatment of incomplete data (92%), contamination (100%), and selective outcome reporting (92%).

Some studies presented an unclear risk on certain domains. The majority of studies did not measure outcomes at baseline (75%) increasing the risk of sampling bias. It was not clear if deception was used in certain studies and, given the nature of the research, knowledge of condition (i.e., allocation) presented an unclear risk for 8% of the studies. The study that exhibited a high risk of bias in the “other” domain was Ramasubramanian and Oliver (2007) whose use of a counter‐arguments exercise prior to the measurement of outcomes may have primed participants, or created a detection or social desirability bias (Fisher, 1993).

In assessing the nonrandomised studies (*n* = 7), the studies naturally had a high risk of bias for maturation (change over time) and were, therefore, all regarded as exhibiting a high overall risk of bias. As well as maturation, participants' knowledge of the true nature of the study posed a high risk for 86% of the nonrandomised studies. For example, Alhabash and Wise (2012, 2015)^9^ explained the premise of their experiment in detail to participants, who would then have been aware that they were in a counter‐attitudinal condition, increasing the risk of response bias. In two studies which used repeated measures over three time points (Frischlich et al., 2018), it is likely that participants became aware of the true nature which increased the risk of carryover effects, social desirability bias as well as the potential for boomerang effects (Brehm, 1966). There was a risk of selective outcome reporting in Kendrick and Fullerton (2004), particularly in introducing group‐level variables into *t*‐tests. The risk of contamination was unclear for 57% of the studies; in Kendrick and Fullerton (2004), the authors reported that the counter‐narrative exposure was broadcast globally and, therefore, there was an increased risk that participants had seen it before. This was also the case for the videos shown by Frischlich et al. (2018), as well as the video game “Peacemaker” used by Alhabash and Wise (2012, 2015).

### 5.3 Synthesis of results

Raw data could not be obtained for one measure of explicit bias (Kendrick and Fullerton, 2004), one measure of realistic threat (Ramasubramanian and Oliver, 2007) or for the singular outcome measured in one randomised control trial (Garagozov, 2013). As such, the findings for these outcomes are presented narratively throughout the following sections, where relevant.

#### 5.3.1 Overall impact of counter‐narrative interventions

The present analysis incorporates 29 effect sizes across 18 studies (many studies measured more than once, conceptually different outcome). This includes 11 randomised control trials (Banas & Richards 2017; Bilewicz & Jaworska, 2013; Bruneau et al., 2017; Cernat, 2001; Cohen et al., 2015; Gonsalkorale et al., 2010; Ramasubramanian & Oliver, 2007; Riles et al., 2018; Saleem et al., 2015), two interrupted time‐series, and five single group pre‐/posttest designs (Alhabash & Wise, 2012, 2015; Čehajić‐Clancy & Bilewicz, 2017; Frischlich et al., 2018; Kendrick & Fullerton, 2004). The studies represent a total sample of 2627 (*M*_age_ 10  = 24.1, 57% female^11^) participants; 1,789 participants were allocated to either a counter‐narrative or control condition. The remainder (*n* = 838) participated in nonrandomised, within‐subjects designs, and served as their own controls. Randomised and nonrandomised studies are analysed separately; it may be useful to consult Table 1 (Theory and Techniques) for the remaining results sections.

##### 5.3.1.1 Randomised studies

**Figure 2:**
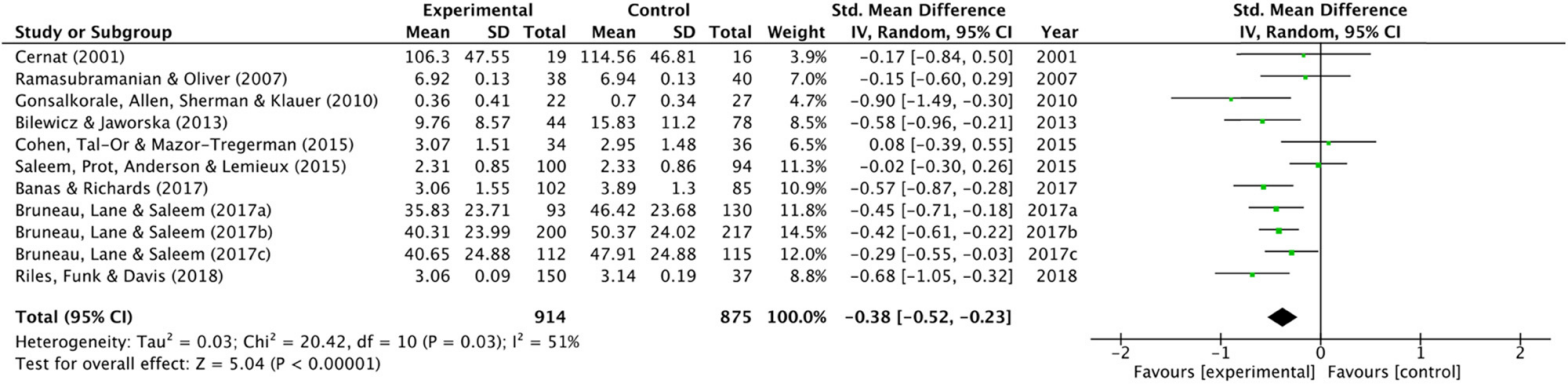
Forest plot of counter‐narrative intervention effects on all risk factors for violent radicalisation compared to a control group

See Figure 2. On average, when all proximal outcomes (i.e., risk factors for violent radicalisation) were pooled to represent each randomised control trial (*n* = 11) the difference between those who did, and those who did not, receive a counter‐narrative intervention was significant, representing a small effect size. Under a random effects model, the standardised mean reduction in risk factor(s) for violent radicalisation was SMD = −0.38; (95% CI, −0.52 to −0.23, *p* = .000). Unsurprisingly, given that the interventions were informed by different theoretical frameworks, measuring conceptually varied outcomes (for example, both implicit and explicit measures), there was modest between‐study heterogeneity (*χ* ^2^ = 20.42 [*p* = .03], *I* ^2^ = 51%, *τ* ^2^ = 0.03).

Subgroup analysis was conducted to determine if the variation among studies could be different depending on the theory and techniques used. Outcome differences were tested by the presence or absence of four theorised key components: counter‐stereotypical exemplars, persuasion, alternative accounts and inoculation. Table 4 presents meta‐analysis statistics separately by technique (i.e., levels of the moderator) (Hunter & Schmidt, 2004, p. 402). The use of persuasive techniques was not found to be effective (*d* = 0.08), while inoculation showed promising effects (*d* = −0.57). However, both effect sizes represented single study samples, limiting the generalisability of both findings based on theory or technique(s). The between‐group differences were not significant and, as such, no further analyses were conducted.

**Table 4:** Separate variance estimates for each group

	*k*	*d*	95% CI	*τ* ^2^	*p*
Technique(s)					
Counter‐stereotypical exemplars	6	−0.40*	−0.70, −0.11	0.08	−.19
Persuasion	1	0.08	−0.39, 0.55	–	–
Alternative account(s)	3	−0.39*	−0.53, −0.26	−0.00	−.67
Inoculation	1	−0.57*	−0.87, −0.28	–	–

* *p* < .05.

##### 5.3.1.2 Nonrandomised studies

**Figure 3:**
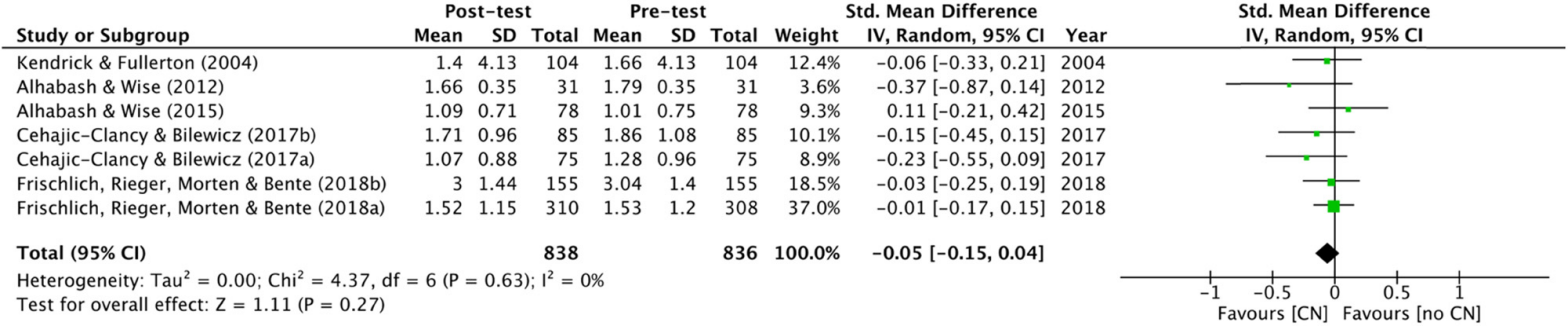
Forest plot of counter‐narrative intervention effects on all risk factors for violent radicalisation postintervention

See Figure 3. For studies that used single group pre‐/posttest designs (*n* = 7), a separate analysis was conducted. Pooling all measured outcomes to represent each study, the effect of the intervention over time was not significant. Under a random effects model, the standardised mean reduction was −0.05; (95% CI, −0.15 to 0.04; *p* = 0.27). In this model, there was little between‐study heterogeneity (*χ* ^2^ = 4.37 [*p* = 0.63], *I* ^2^ = 0%, *τ* ^2^ = 0.00), suggesting that the finding was consistent across studies. Four studies used persuasive techniques (Alhabash & Wise, 2012, 2015; Frischlich et al., 2018), Kendrick and Fullerton (2004) did not specify their theory or techniques but used counter‐stereotypical information while Čehajić‐Clancy and Bilewicz, (2017) delivered an eclectic intervention that included moral exemplars.

Given the between‐study heterogeneity among the RCTs, as well as the disparate findings between randomised and nonrandomised studies (the former being superior, but the latter, nonetheless, homogenous and of interest), separate analyses were subsequently conducted for each risk factor (perceived group threat and in‐group favouritism/out‐group hostility), including subgroups for each subcategory risk factor where possible. Randomised and nonrandomised designs were, again, analysed separately. It may be useful to consult Table 2 for the following subsections.

#### 5.3.2 Perceived group threat

The present analysis incorporates effect sizes measuring participants' perception of threat from their adversarial group. Across both randomised and nonrandomised studies, this represents a total sample of 2,046 participants; 1,662 participants were allocated to either a counter‐narrative or control condition (Banas and Richards 2017; Bilewicz & Jaworska, 2013; Cernat, 2001; Cohen et al., 2015; Riles et al., 2018; Saleem et al., 2015). The remainder (*n* = 384) participated in single group pre‐/posttest designs (Alhabash & Wise, 2012, 2015; Čehajić‐Clancy and Bilewicz, 2017). In many cases, the studies measured a combination of “symbolic” threat perceptions (e.g., perceived differences in motives, values or standards between the in‐group and the out‐group) and “realistic” threat perceptions (e.g., perceived threats to one's safety or existence).

##### 5.3.2.1 Randomised studies

**Figure 4:**
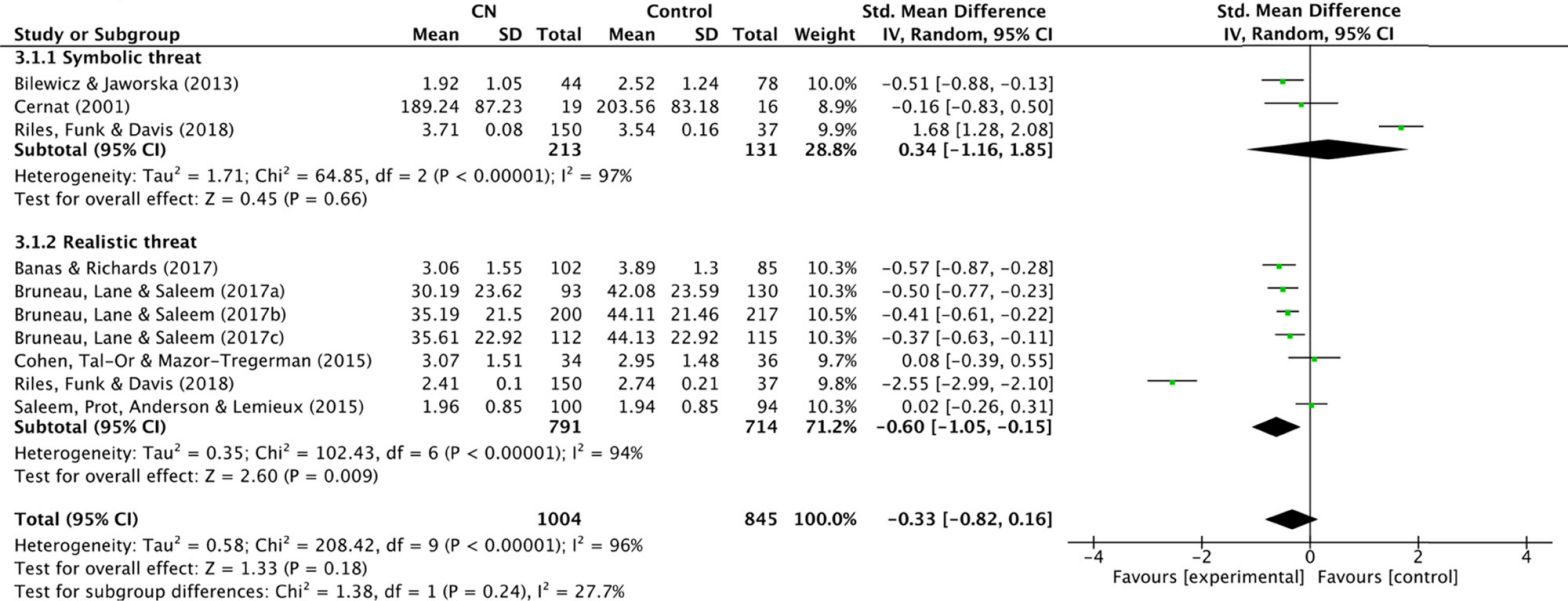
Forest plot of counter‐narrative intervention effects on all perceived group threat (both symbolic and realistic) compared to a control group

This analysis incorporates 10 effect sizes from nine randomised studies. As shown in the forest plot in Figure 4, the intervention effect for randomised studies which measured both symbolic and realistic threat (i.e., perceived group threat) was not significant. The mean reduction was, SMD = −0.33 (95% CI, −0.82 to −0.16; *p* = .18). Although there was significant between subject heterogeneity (*χ* ^2^ = 208.42 [*p* = .000], *I* ^2^ = 96%, *τ* ^2^ = 0.58), the test for subgroup differences (between symbolic threat outcomes and realistic threat outcomes) was not significant (*p* = .24).

Looking at the subgroups, the intervention effect for randomised studies targeting symbolic threat not significant, SMD = 0.34 (95% CI, −1.16 to 1.85; *p* = .66). However, there was significant between‐study heterogeneity (*χ* ^2^ = 64.85 [*p* = .000], *I* ^2^ = 97%, *τ* ^2^ = 1.71), likely explained by Riles et al. whose intervention saw a significant increase in symbolic threat (i.e., in the wrong direction, SMD = 1.68). Their effect for realistic threat was also significant, but in the intended direction (i.e., a reduction).

On average, realistic threat decreased by SMD = −0.60 (95% CI, −1.05 to −0.15; *p* = 0.01), with, again, significant between subject heterogeneity (*χ* ^2^ = 208.42 [*p* = .000], *I* ^2^ = 96%, *τ* ^2^ = 0.58).

##### 5.3.2.2 Nonrandomised studies

**Figure 5:**
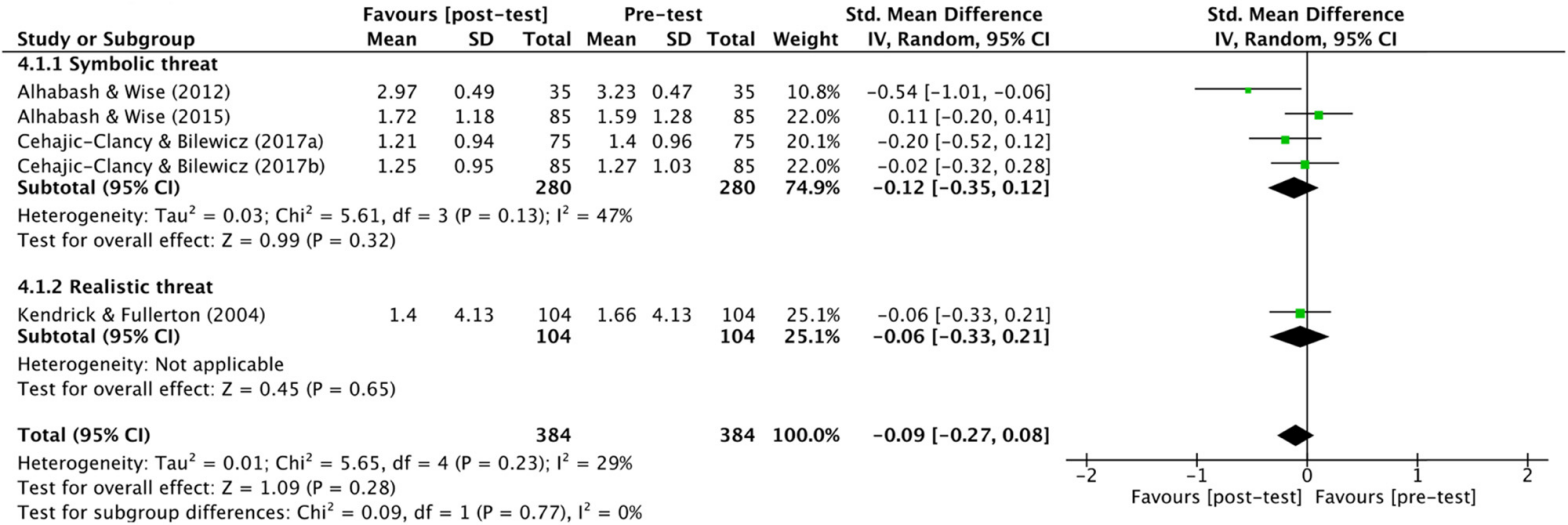
Forest plot of posttest changes following a counter‐narrative intervention on symbolic threat

For the nonrandomised studies, only Kendrick and Fullerton (2004) measured realistic threat, with the remainder measuring symbolic threat (as shown in the forest plot in Figure 5). When pooled, the intervention effect was not significant, SMD = −0.09 (95% CI −0.27 to 0.08; *p* = .28), with minimal between‐study heterogeneity (*χ* ^2^ = 5.65 [*p* = .23], *I* ^2^ = 29%, *τ* ^2^ = 0.01). Across both symbolic and realistic threat, this finding was heterogeneous, with no significant subgroup differences (*p* = .77). Thus, in within‐groups samples, the counter‐narrative interventions do not appear to reduce perceived group threat.

#### 5.3.3 In‐group favouritism and/or out‐group hostility

The current analysis incorporates 11 effect sizes representing in‐group favouritism and/or out‐group hostility. This includes seven randomised control trials (Bilewicz & Jaworska, 2013; Bruneau et al., 2017; Cernat, 2001; Gonsalkorale et al., 2010; Ramasubramanian & Oliver, 2007), and four single group pre‐/posttest designs (Alhabash & Wise, 2012, 2015; Čehajić‐Clancy & Bilewicz, 2017). The studies represent a total sample of 1,420 participants (demographic descriptive statistics were not consistently provided); 1,151 participants were allocated to either a counter‐narrative or control condition. The remainder (*n* = 269) participated in nonrandomised, before‐and‐after studies (i.e., no control group). Again, randomised and nonrandomised studies are analysed separately.

##### 5.3.3.1 Randomised studies

**Figure 6:**
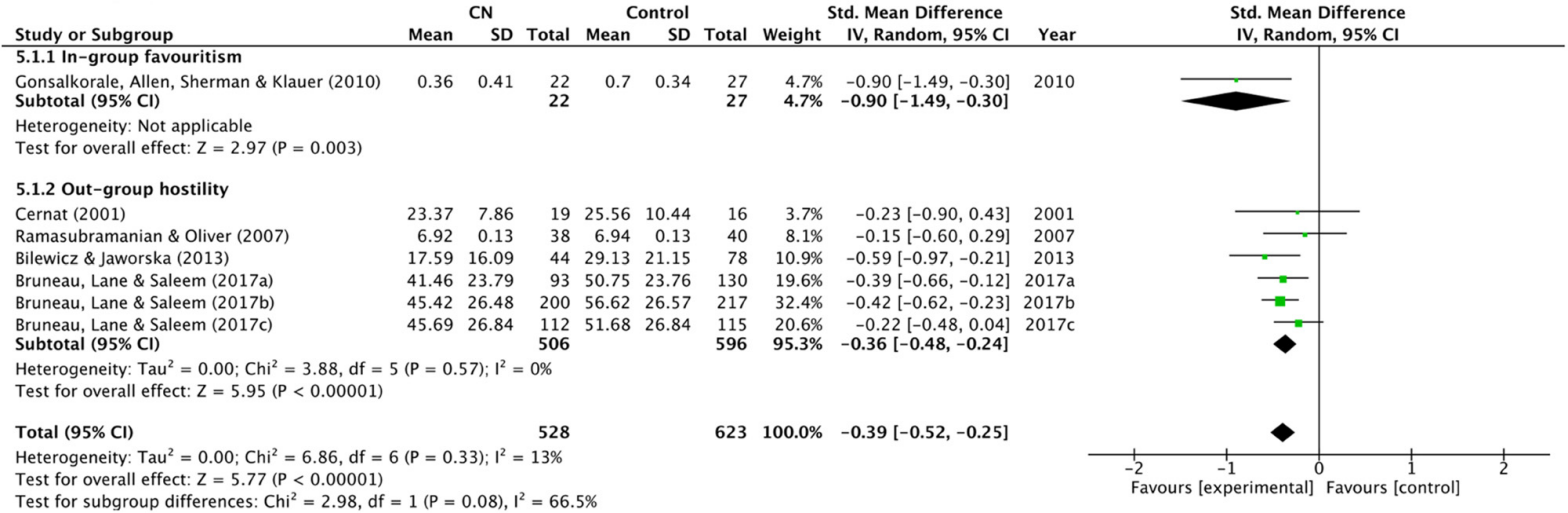
Forest plot of counter‐narrative intervention effects on in‐group favouritism and out‐group hostility compared to a control group

As shown in the forest plot in Figure 6, the intervention effect for randomised studies which measured in‐group favouritism and out‐group hostility was significant, SMD = −0.39 (95% CI, −0.52 to −0.25; *p* = .000), with minimal between‐subject heterogeneity (*χ* ^2^ = 6.86 [*p* = .33], *I* ^2^ = 13%, *τ* ^2^ = 0.00). In other words, those in the counter‐narrative condition showed a decrease in the overall risk factor compared to a control group, with a small‐medium effect; this was consistent across the subgroups. The effect of the intervention on out‐group hostility also showed a small effect, SMD = −0.36 (95% CI, −0.48 to −0.24; *p* = .000).

Data for in‐group‐favouritism was only available for one randomised study, and the effect was significant with a very large effect size, SMD = −0.90 (95% CI, −1.49 to −0.30; *p* = .003). In terms of implicit bias, Garagozov (2013) narratively reported that their “Common Suffering” counter‐narrative was the most effective at reducing implicit bias, while “Blame the Russians” was the least; the latter findings are to be interpreted with caution as no effect sizes were provided.

##### 5.3.3.2 Nonrandomised studies

**Figure 7:**
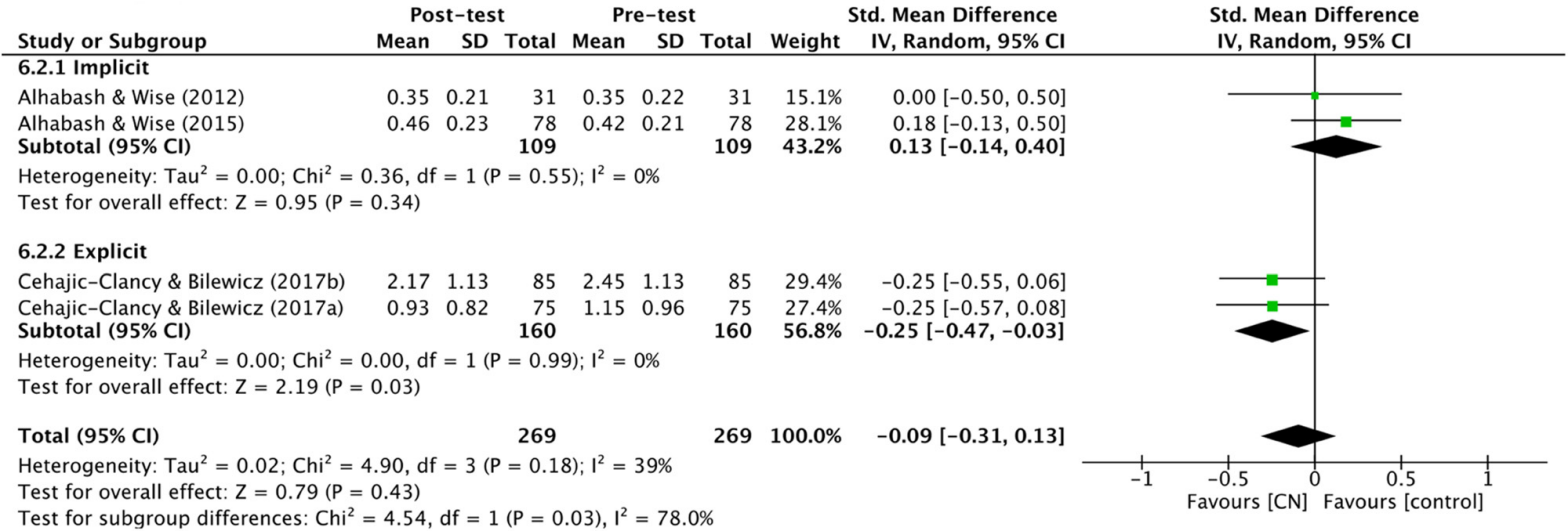
Forest plot of posttest changes following a counter‐narrative intervention on out‐group hostility (both implicit and explicit)

In the studies that used single group pre‐/posttest designs and measured out‐group hostility, the implicit (e.g., the AMP) and explicit measures (e.g., negative evaluations of the out‐group) of this outcome were observed separately. As shown in the forest plot in Figure 7, there were significant subgroup differences (*p* = .03).

With two separate samples, Čehajić et al. (2017) measured out‐group hostility on explicit measures, and the effect was significant, SMD = −0.25 (95% CI, −0.47 to −0.03; *p* = .003), with minimal between‐study heterogeneity (*χ* ^2^ = 0.00 [*p* = .99], *I* ^2^ = 0%, *τ* ^2^ = 0.00). Conversely, the intervention effect for Alhabash and Wise (2012, 2015) who measured out‐group hostility using implicit measures was not significant, SMD = 0.13 (95% CI −0.14 to 0.40; *p* = .34), with, again, minimal between‐study heterogeneity (*χ* ^2^ = 0.36 [*p* = .55], *I* ^2^ = 0%, *τ* ^2^ = 0.00). This suggests that, while the counter‐narrative seems to be effective (pre‐ to posttest) at reducing bias on an explicit level, this is not the case on an implicit level. Data could not be obtained for Kendrick and Fullerton (2004) measure of explicit bias (“attitudes towards the US people”), but it is reported that the effects of the counter‐narrative intervention did not reduce this bias to a level of significance.

### 5.4 Sensitivity analysis

Two meta‐analyses required sensitivity analysis. The first related to the overall impact of counter‐narrative interventions (Section 5.3.3.1; Figure 2). This required more arbitrary decision‐making and therefore, sensitivity analysis was conducted to determine the impact of certain decisions. With multiple study outcomes, the authors chose to average all the scores for outcomes categorised as risk factors for violent radicalisation in the original analysis, creating a single, standardised mean difference for each study. However, in line with The Campbell Collaboration policies and procedures, an alternative approach can be used whereby outcomes are chosen using specific decision criteria (e.g., reliability, validity or relevance).

Using this approach, a sensitivity analysis was conducted in which the most reliable outcome (reported as Cronbach's *α*) was chosen. In cases where no reliability analysis was provided, the most relevant outcome was chosen. These criteria were applied to the relevant studies individually. No notable differences were observed (see Table A8, Appendix A). When all these changes were applied together (Table 5, previous page), although there was a moderate increase in effect size (−0.39 to −0.58), the between‐effect difference was not statistically significant (*χ* ^2^ = 0.82, df = 1; *p* = .37).

**Table 5:** Sensitivity analysis incorporating multiple changes for analysis 1 (Figure 2)

Analysis	Study	Decision	SMD	95% CI	*p*	Heterogeneity
Original analysis	Cernat (2001); Bilewicz and Jaworska (2013); Saleem et al. (2015); Bruneau et al. (2017); Riles et al. (2018)	Multiple outcomes in a single study are pooled to create an average (see Table A8^a^)	−.39	−0.55 to −0.24	.000	*χ* ^2^ = 19.38 [*p* = .02], *I* ^2^ = 54%, *τ* ^2^ = 0.03
Sensitivity analysis	Cernat (2001); Bilewicz and Jaworska (2013); Saleem et al. (2015); Bruneau et al. (2017); Riles et al. (2018)	The most valid/relevant outcome is chosen from each study (see Table A8^a^)	−.58	−0.94 to −0.21	.002	*χ* ^2^ = 107.29 [*p* = .000], *I* ^2^ = 92%, *τ* ^2^ = 0.30
Result	Although there was a moderate increase in effect size, the between‐effect difference was not statistically significant (*χ* ^2^ = 0.82, df = 1; *p* = .37)					

a Table A8 is included in Appendix A.

The second sensitivity analysis was concerned with risk of bias with the nonrandomised studies; in assessing the risk of bias for these studies (*n* = 7), all were regarded as high risk. However, it was noted that two studies (Frischlich et al., 2018) posed particularly high risk for carryover effects, and performance biases. As such, the second analysis was re‐run, excluding these studies (see Table 6). The removal of these studies resulted in a stronger effect size, but this remained nonsignificant. The between‐effect difference was, also, not significant.

**Table 6:** Sensitivity analysis excluding studies with high risk of bias for analysis 2 (Figure 3)

Analysis	Study	Decision	SMD	95% CI	*p*	Heterogeneity
Original analysis	Kendrick and Fullerton (2004); Alhabash and Wise, (2012, 2015); Čehajić‐Clancy & Bilewicz, (2017); Frischlich et al. (2018)	To include all, nonrandomised studies for which data could be obtained, irrespective of risk of bias	−0.05	−0.15 to −0.04	.27	*χ* ^2^ = 4.37 [*p* = .63], *I* ^2^ = 0%, *τ* ^2^ = 0.00
Sensitivity analysis	Alhabash and Wise, (2012, 2015); Čehajić‐Clancy & Bilewicz, (2017)	To exclude studies with a high risk of bias on a number of items (carryover effects, performance bias) from the analysis	−0.12	−0.30 to 0.06	.19	*χ* ^2^ = 3.42 [*p* = .19], *I* ^2^ = 12%, *τ* ^2^ = 0.00
Result	Although there was a moderate increase in effect size, the effect remained nonsignificant, and the between‐effect difference was not statistically significant (*χ* ^2^ = 4.37, df = 6; *p* = .63)					

### 5.5 Publication bias analysis

**Figure 8:**
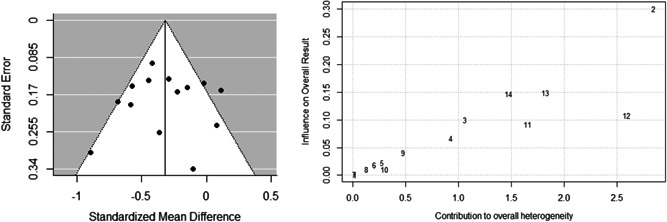
Funnel (left) and Baujat (right) plots

Publication bias was assessed with all the randomised and nonrandomised studies used throughout the analysis. The effects were heterogeneous, (*χ* ^2^ = 30.30 [*p* = .004], *I* ^2^ = 57%, *τ* ^2^ = 0.04) so a Baujat plot was produced to explore possible contributors to heterogeneity. As shown in Figure 8 (below), Alhabash and Wise (2015) was the leading contributor.^12^ To further examine the observed heterogeneity, Egger's regression test (*z* = −0.30, *p* = .77) and the rank correlation test (Kendall's *τ* = −0.03, *p* = .92) were conducted. Both were not significant, interpreted as a clear lack of evidence of publication bias. This was corroborated when the study effects were plotted against their standard errors in a funnel plot and the distribution of studies was observed to be symmetrical. As such, no publication bias is reported in the overall analysis.

The publication bias analysis was not repeated for the remaining meta‐analyses. The authors make the point that the categorisation of measured outcomes onto secondary outcomes (see Table A7, Appendix A) was unavoidably biased, and testing for different reporting biases within the individual analyses would reflect this, regardless of the true risk of bias.

To avoid any potential biasing of the meta‐analysis due to multiple studies^13^ within the same publication (i.e., Bruneau et al., 2017), the analysis on the overall impact of the intervention was run again, removing specific studies and observing any differences in overall effect or between‐study heterogeneity (see Table 7). Removing the studies with the largest effect size(s) (Bruneau et al., 2017) *or* smallest effect size (Bruneau et al., 2017) was not found to change the overall effect, nor the heterogeneity (which both remained significant). The same was observed when all three studies (2017a, 2017b, 2017c) were removed. The authors concluded that the inclusion of these studies likely did not increase the potential for bias.

**Table 7:** Sensitivity analysis incorporating multiple changes for analysis 1 (Figure 2)

	Included studies	Excluded studies	SMD	95% CI	*p*	Heterogeneity
Original analysis	Cernat (2001); Gonsalkorale et al. (2010); Bilewicz and Jaworska, (2013); Cohen et al. (2015); Saleem et al. (2015); Banas and Richards (2017); Bruneau et al. (2017; Riles et al. (2018)	None.	−0.39	−0.55 to −0.24	.000	*χ* ^2^ = 19.38 [*p* = .02], *I* ^2^ = 54%, *τ* ^2^ = 0.03
Sensitivity analysis	Cernat (2001); Gonsalkorale et al. (2010); Bilewicz and Jaworska, (2013); Cohen et al. (2015); Saleem et al. (2015); Banas and Richards (2017); Bruneau et al. (2017); Riles et al. (2018)	Bruneau et al. (2017)^a^	−0.39	−0.57 to −0.21	.000	*χ* ^2^ = 19.19 [*p* = .01], *I* ^2^ = 58%, *τ* ^2^ = 0.04
	Cernat (2001); Gonsalkorale et al. (2010); Bilewicz and Jaworska, (2013); Cohen et al. (2015); Saleem et al. (2015); Banas and Richards (2017); Bruneau et al. (2017); Riles et al. (2018)	Bruneau et al. (2017)^b^	−0.41	−0.59 to −0.23	.000	*χ* ^2^ = 18.74 [*p* = .02], *I* ^2^ = 57%, *τ* ^2^ = 0.04
	Cernat (2001); Gonsalkorale et al. (2010); Bilewicz and Jaworska, (2013); Cohen et al. (2015); Saleem et al. (2015); Banas and Richards (2017); Riles et al. (2018)	Bruneau et al. (2017)	−0.40	−0.67 to −0.14	.000	*χ* ^2^ = 18.58 [*p* = .01], *I* ^2^ = 68%, *τ* ^2^ = 0.08

a This study had the largest effect size of all Bruneau et al. (2017) studies.

b This study had the smallest effect size of all Bruneau et al. (2017) studies.

## 6 DISCUSSION

### 6.1 Summary of main results

The objective of this review was to provide a synthesis of the effectiveness of targeted counter‐narrative interventions in reducing the risk of violent radicalisation by asking the question: what is the impact of targeted counter‐narrative interventions on violent radicalisation (primary outcomes) and/or risk factors for violent radicalisation (secondary outcomes)?

#### 6.1.1 Primary outcomes

The authors considered primary outcomes related to violent radicalisation to include behavioural intention to engage in manifestations of violent extremism, including terrorism. Although there was a limited number of studies measuring such outcomes, the findings were nonetheless discouraging. Upon exposure to their counter‐narrative, Saleem et al. (2015) measured participants support for military action in Muslim countries, and found that their intervention was not effective on this outcome (nor on any of the outcomes measured in the study). Frischlich et al. (2018) conducted two, multifaceted studies which measured participants' agreement with statements purporting the instrumentality of violence across two violent extremist contexts, observing no effects. Agreement was measured at baseline, upon exposure to two violent extremist narratives and, finally, upon exposure to two counter‐narratives designed to induce transportation. However, due to the high risk of response bias (as well as insufficient evidence demonstrating the efficacy of either manipulation), the effects of the intervention on these primary outcomes, while supporting those of Saleem et al., are tentatively interpreted.

##### 6.1.1.1 Summary

Therefore, in response to the first part of the review question, the authors have found little evidence that counter‐narrative interventions are effective at targeting primary outcomes related to violent radicalisation. However, the scarcity of sufficient, high‐quality studies measuring these outcomes means that this evaluation cannot, yet, be regarded as conclusive and, indeed, may change with the emergence of further, rigorous research.

#### 6.1.2 Secondary outcomes

For secondary outcomes, there was some disparity on intervention effectiveness. Overall, when pooling all outcomes across all randomised studies (representing 11 effect sizes), the intervention showed a small effect (SMD = −0.38; 95% CI, −0.52 to −0.23; *p* = .000). The studies with the largest effect sizes were exemplar‐based, exposing participants to counter‐attitudinal positive exemplars of Black people (Gonsalkorale et al., 2010), Muslims (Riles et al., 2018), and Polish people (Bilewicz & Jaworska, 2013), using a variety of mediums, including computer‐based tasks, movie clips, and stories. However, the interventions had different effects on different risk factors.

##### 6.1.2.1 Perceived group threat

The concept of threat perception as a catalyst for the endorsement or perpetration of manifestations of violent extremism is supported by decades of research on intergroup attitudes and relations (Kruglanski et al., 2014; Stephan et al., 1985). The findings from this review suggest that counter‐narrative interventions can target this risk factor in different ways.

For example, Bilewicz and Jaworska, (2013), Cernat (2001), and Riles et al. (2018) measured outcomes which drew upon symbolic threat concepts by measuring perceived differences in morals, culture and values (e.g., “perceived similarity” to Polish people; “social stigma” towards Muslims). Overall, the counter‐narratives were not found to be effective on this risk factor (*d* = 0.34). However, there were, nonetheless, some discrepancies between studies. In their counter‐narrative, Bilewicz and Jaworska, (2013) had participants read stories of “Heroic Helpers”, as well as watch a presentation by a Polish person who helped Jews during the Holocaust. Riles et al. (2018) also used “helping” exemplars, showing clips of Muslims helping Americans in movies and TV shows. However, while Bilewicz and Jaworska, (2013) demonstrated a medium effect of the “Heroic Helpers” intervention on participants' perceived similarity to Polish people (*d* = 0.51), the same was not found for Riles et al.; despite obvious parallels in design and measurement, their depiction of Muslim protagonists demonstrating counter‐stereotypical, prosocial behaviours increased participants social stigma towards Muslims, demonstrating among the largest effects of any study in the review (*d* = 1.68). This disparity signals an important point of discussion, not only for evaluation of counter‐narratives, but the for active ingredients incorporated into their design.

###### 6.1.2.1.1 Active ingredients

In reviews of this nature, such discrepancies in intervention effect can be traced back to two study components: the intervention, or the measurement. Both studies used validated measures with acceptable reliability (*α* = .81; *α* = .89) and plausible constructs for study comparison (“perceived similarity” and “social stigma”). Therefore, the discrepancies likely arise from differences in intervention design. When focus is shifted to the counter‐narrative itself, it is clear that while Riles et al. showed participants fictional clips of Muslims being helpful in the United States, Bilewicz and Jaworska, (2013) introduced participants to a member of the out‐group, who then presented the counter‐attitudinal content. In line with the “Contact Hypothesis” (Allport, 1954) increased exposure to an adversary has been found to decrease levels of hostility (see Pettigrew & Tropp, 2006); at least outwardly, this may explain why participants in Bilewicz et al. exemplar‐based intervention reported significantly increased similarity to the adversarial group, while Riles et al. did not.

In many ways, Bilewicz et al. delivered an *eclectic* intervention, informed by other theoretical frameworks other than those specific the counter‐narrative concept (i.e., counter‐stereotypical exemplars). In other words, the specific technique or “active ingredient” in the intervention may *not* have been the counter‐narrative one.

Lack of specificity, in terms of techniques, arises as a challenge several times in this review. Čehajić‐Clancy and Bilewicz, (2017) attempted to increase participants' awareness of the depth and variability of their adversarial group through the use of “moral exemplars”. Using a single group pre‐/posttest design, they measured participants' belief in reconciliation, intergroup anxiety, and levels of forgiveness, before and after the intervention. In both studies, the interventions were not effective (*d* = −0.15 to −0.23). However, the learning that can be taken from this research is diluted by a “kitchen sink approach” to intervention design, which included films, film trailers, case‐studies, contact, and group‐work over an 8–9 week period. As reviewers, a balance must be struck between isolating the individual components of an intervention, which may mean excluding studies with multifarious designs, and, simply, ending up with an empty review due to inflexible parameters. This is the reality of conducting systematic research in a developing area. However, rather than disregarding research which does not fit a mould, researchers must strive to better synthesise, and encourage more rigorous methodologies moving forward.

###### 6.1.2.1.2 Symbolic versus realistic

Although differing intervention components and theoretical frameworks certainly explain some of the heterogeneity above, there were nonetheless some observable, discrepant effects *within* the risk factors themselves. This was particularly the case between symbolic and realistic threat perceptions.

This is unsurprising as they refer to two, different concepts. In line with realistic group conflict theories (see Jackson, 1993 for extensive review) realistic threat perceptions arise due to genuine, or “realistic” threats to the safety, or existence of one's in‐group. In the subgroup analysis (Figure 4), outcomes subcategorised under realistic threat included measures of social distance (Riles et al., 2018), antigovernment attitudes (Banas & Richards 2017) and perceptions of the out‐group as violent (Bruneau et al., 2017). Compared to symbolic threat, the overall subgroup effect on all measures of realistic threat was significant, and negative (*d* = −.60; 95% CI, −1.05 to −0.15; *p* = .01), indicating that these psychological constructs (symbolic versus realistic threat) respond differently to counter‐narrative techniques. A case in point is Riles et al. (2018), whose intervention decreased realistic threat, displaying a very large effect size (*d* = −2.55), but whose effects on symbolic threat were adverse (*d* = 1.68). Even anecdotally, within realistic threat, Kendrick and Fullerton (2004) found that their depiction of the “happy lives” of Muslims living and working in the United States improved certain realistic threat outcomes, but not others.

These effects illustrate the complexity of perception, particularly in the context of threat. While the use of counter‐narrative interventions may decrease some risk factors, their effects on others are unpredictable. The evidence from this review, from a combination of randomised and nonrandomised studies, indicates that counter‐narratives can effectively target perceptions of realistic threat but, conversely, are likely to be ineffective at targeting symbolic threat, as measured by adverse stereotypes (Alhabash & Wise, 2015; Cernat, 2001), reconciliatory beliefs (Čehajić‐Clancy & Bilewicz, 2017) and, as mentioned, social stigma (Riles et al., 2018). The verdict as to which risk factor is more predictive of violent radicalisation is beyond the scope of this review. However, the authors propose that future counter‐narrative interventions reflect the complexity of their prospective outcomes, and consider that an ineffective counter‐narrative intervention, beyond having no effect, has the potential to have exacerbating effects.

###### 6.1.2.1.3 Persuasion

However, in the majority of cases, the studies in the review used specific, comparable techniques. On measures of realistic threat, two interventions incorporated counter‐arguments in their counter‐narrative design(s); the application of contradictory information to a message, with the intention of refuting it (Wheeler et al., 2007, p. 151). Informed by inoculation theory (McGuire, 1961a, 1961b), Banas and Richards (2017) warned participants that a persuasive appeal was impending (“explicit forewarning”), before offering some prospective counter‐arguments (“refutational pre‐emption”) against what was to be antigovernment, conspiratorial propaganda. Along a similar vein, Cohen et al. (2015) countered antidemonstration arguments by having participants read a fictional “debate” between discordant friends on the topic of on‐campus demonstrations. They also manipulated the “virtuousness” of the prodemonstration character, in an attempt to increase participants' agreement with her arguments through a process of “identification” (p. 4) through persuasion; the feeling of being absorbed into a story through the position of the character with whom one identifies (see Cohen, 2001). However, while Banas et al. demonstrated one of the largest overall effect sizes (*d* = −.0.57), Cohen et al. (2015) showed no effect and, in fact, showed a (very slight) increase in participants perception of threat (“It should be forbidden for [Arab students] to demonstrate in the heart of the campus”). This is not the only evidence that persuasive techniques may be ineffective components of counter‐narrative interventions.

In two nonrandomised studies, Alhabash and Wise (2012, 2015) used persuasive techniques in the form of a video game designed to encourage self‐persuasion through transportation (Green & Brock, 2002) and, again, processes of identification. Although this approach saw success in reducing symbolic threat through an understanding of Palestinian motives (e.g., agreement with statements such as, “[Palestinians] want peace” and “[Palestine] is not responsible for violence”) (*d* = −0.54), in terms of symbolic values (“Palestinians are cruel”), and measures of out‐group hostility, the intervention was ineffective, and worked in the wrong direction. These findings paint a complex picture on the use of persuasive techniques to reduce participants' risk of violent radicalisation. It may be the case that participants' awareness of the persuasive appeal influenced their response; it may also be the case that the tactic, itself, is discordant to the overall purpose of counter‐narratives. Regardless, the evidence from this review does not support the use of persuasive techniques in the design of counter‐narratives intended to reduce perceptions of threat, or out‐group hostility.

##### 6.1.2.2 In‐group favouritism/out‐group hostility

The perception that certain out‐groups are inferior to one's in‐group is an important component of a radical belief system (Doosje et al., 2013; Loza, 2007), and a defining characteristic of violent extremism, in general (Berger, 2017, 2018). In the meta‐analysis based on 7 effect sizes reporting the impact of counter‐narrative interventions on in‐group favouritism and out‐group hostility (across randomised studies), there was a small, significant effect (*d* = −0.39). However, the interventions were, again, comprised of different intervention components.

###### 6.1.2.2.1 Alternative accounts

On measures of out‐group hostility alone (Figure 6), all such studies used a “feeling thermometer”, which allowed for the “active ingredients” to be elucidated. The effectiveness of Bilewicz and Jaworska, (2013) contact intervention, which incorporated counter‐stereotypical exemplars, has been discussed in Section 6.1.2.1.1. The next most promising studies in this analysis were Bruneau et al. (2017), who were the only studies to challenge the dominant narrative by using what they termed an “alternative account of events”. Fallaciously perceiving a side, particularly in conflict, as violent has been said to compromise third‐party sympathy (Vandello et al., 2011); their counter‐narratives, therefore, attempted to restore favourability to the Palestinian “side” by providing an account with ran counter to that of the dominant narrative. This was done by showing participants a documentary film trailer depicting Palestinians engaging in nonviolent resistance. This method can be said to disrupt the false binaries of the dominant narrative; those that exclusively associate Palestine with violence). This use of alternative accounts has been posited as a promising avenue for counter‐narrative design (Braddock et al., 2016; United Nations, 2008), and the findings from this review support this approach. Offering a plausible alternative to popular discourse does appear to reduce out‐group hostility (as well as realistic threat perceptions) towards an out‐group.

###### 6.1.2.2.2 Fiction or nonfiction

However, although these studies used nonfictional content (through the use of documentary), it is not clear if this component is in any way integral to the efficacy of the intervention. For example, several other nonfictional approaches were not found to be effective. In the same analysis, Cernat (2001) had participants read exemplar‐based, historical accounts, but did *not* report significant effects on out‐group hostility (*d* = −0.23). Similarly, although this study was synthesised narratively, Ramasubramanian and Oliver (2007) found that participants who read, counter‐stereotypical newspaper articles (i.e., nonfictional content) did not report more positive ratings on the feeling thermometer to a level of significance compared to a control group (*d* = −0.15). On other risk factors, Saleem et al. (2015) used positive exemplar‐based news clips, and found no effects on measures of symbolic threat (*d* = −0.02). Riles et al. (2018), conversely, used fictional exemplars and found that the intervention was effective for realistic threat, but exacerbated levels of symbolic threat. Although these findings broadly support the use of nonfictional, rather than fictional content in counter‐narratives, the scarcity of interventions employing the same techniques with respective, fictional and nonfictional content means that the authors cannot confidently determine the effectiveness of one over the other.

##### 6.1.2.3 Summary

Therefore, in response to the second part of the review question, the authors have found some evidence that counter‐narratives can be effective at targeting certain, risk factors for violent radicalisation. These risk factors include realistic threat, in‐group favouritism, and out‐group hostility (explicit, rather than implicit). However, across different intervention components, the effects are somewhat mixed, and may change with the emergence of new evidence. The use of alternative accounts, and counter‐arguments showed promising effects on these risk factors. However, the use of persuasive techniques were not found to be effective, on any risk factors.

### 6.2 Overall completeness and applicability of evidence

Several international actors such as the ICCT, the Institute for Strategic Dialogue (ISD), and the Radicalisation Awareness Network (RAN) have commented on the need for evaluation in the design of counter‐narratives (Saltman, Dow, & Bjornsgaard, 2016), resulting in numerous counter‐narrative initiatives and strategies. While this review offers a comprehensive analysis of the effectiveness of certain, targeted counter‐narrative interventions on reducing propensity towards violent radicalisation, it must be acknowledged that the scope and span of counter‐narrative interventions likely extends beyond the 19 studies included in this review. This is the case for two reasons.

First, while many counter‐narrative strategies appeared in the initial searches (e.g., Frennett et al., 2015; Macnair & Frank, 2017), the majority did not meet the inclusion criterion for outcomes related to violent radicalisation. Instead, the evaluative components of many of these campaigns were more reflective of feasibility, rather than effectiveness. Metrics such as likes, comments, “bounce‐and‐exit rates”, or shares (see Denaux & Rollo, 2019) may tell us about the counter‐narrative campaign from a practical perspective, but they are not empirically supported risk factors for violent radicalisation, or components of a radical belief. As such, they cannot indicate if the target of the campaign has a reduced risk of transitioning into violent extremism. It is for these reasons that many published, potentially informative counter‐narrative campaigns could not be included in the synthesis, despite their relevance to the area at large.

Second, it may be the case that certain, counter‐narrative strategies were not identified through the search strategy to begin with. This is not necessarily a critique of the strategy itself. Instead, it refers to what Sageman terms the “stagnation” (2014, p. 565) of terrorism research, with regards to government‐funded projects not being made available to academics, creating an “unbridgeable gap between academic and the intelligence community” (p. 573).^14^ This is not to suggest that potentially relevant studies are being withheld; however, with the “counter‐narrative” becoming common currency in the world of countering violent extremism, it is unlikely that more attempts at designing, and evaluating them, have not been attempted at governmental levels.

### 6.3 Quality of the evidence

The 19 studies were assessed according to the GRADE approach for evaluating quality of evidence. Randomised control trials were first graded as “high”, and downgraded accordingly depending on the severity of the study limitations. Nonrandomised studies were first graded as “low” quality, and upgraded or downgraded accordingly, using the same criteria. Case series, interrupted‐time‐series or uncontrolled longitudinal designs were graded as “very low” quality, and ungraded if necessary.

The results of the quality analysis are provided in Table D2 (Appendix D). Just over half of the studies (58%) were rated as moderate (*n* = 6) to high (*n* = 5) quality, with one randomised study of low quality. Randomised studies downgraded from high to moderate were generally characterised by the following limitations: using outcome measures with poor reliability, small (or unequal) sample sizes, potentially uncredible control groups (e.g., a control group that did “nothing”, rather than an active control), and the potential for crossover effects. For example, the randomised study (Cernat, 2001) which was double‐downgraded to “low” shared the above limitations and, additionally, the intervention was poorly informed (i.e., not guided by a specific theoretical framework).

The remaining studies were all nonrandomised and categorised as “low” (*n* = 2) or “very low” (*n* = 5). Similarly, studies were generally downgraded for using outcome measures with poor reliability. The risk of crossover, or practice, effects for these studies was, also, logically higher. One nonrandomised study was double‐downgraded as, alongside the limitations outlined above, the intervention not informed by a specific theoretical framework, and it used single‐item measures. Studies were upgraded for design strengths such as the use of deception, reliable or validated outcome measures, and large effect sizes.

Violent radicalisation and, in particular, the evaluation of interventions to prevent it, is a challenging area to conduct high quality research. Quality standards such as those described above require that researchers deliver theoretically informed interventions and measure empirically supported outcomes using validated, reliable measures. However, the normal challenges encountered at various stages of the study design process are much thornier in this area. Although research into the process(es) of violent radicalisation is ever‐expanding, leading to the identification of specific risk factors, robust theories, and novel ways of testing them, the field is nonetheless in the early stages of *theory‐building*, mid‐“leap” between exploratory and explanatory phases (Silke, 2001, p. 2). Ultimately, this an area which does not, yet, have an explanatory understanding of its central problem (i.e., violent radicalisation as a process leading to the perpetration of violent extremism or terrorism), or how to measure it. For this reason, stringent quality appraisal, although insightful, may be premature. Nonetheless, efforts to reduce sampling bias (and unequal sample sizes), utilise measures with sufficient construct validity and, finally, introduce credible, comparable control conditions would help ameliorate a number of biases.

### 6.4 Limitations and potential biases in the review process

There are several limitations that could affect the results of the present review. First, the literature base was limited, as is to be expected with research of this nature. For this reason, the target populations in the studies had varying, dominant narratives, ranging from entrenched ideas about conflicts, to prejudicial leanings towards Muslims. This rendered it difficult to determine the effects of the intervention(s) on different, dominant narratives, as well as embeddedness of the dominant narrative(s) to begin with. The sparse literature base also leads to another limitation in the review; a lack of comparable, valid outcomes. Although the outcomes in this review all measured outcomes related to violent radicalisation, and followed explicit protocol in defining acceptable outcome measures, they were conceptually broad and, despite every effort to preserve each outcome's original construct, the process of conceptually mapping the outcomes on to risk factors for violent radicalisation was, nonetheless, subject to bias.

## 7 AUTHORS' CONCLUSIONS

### 7.1 Implications for practice and policy

The findings from this review have implications for those seeking to prevent violent radicalisation into terrorism by challenging dominant, violence‐promoting narratives. The findings from this review illustrate the complexity of violent‐radicalisation in terms of secondary outcomes or risk factors. While the use of counter‐narrative interventions may decrease some risk factors, their effects on others are unpredictable. The evidence from this review, from a combination of randomised and nonrandomised studies, indicates that counter‐narratives can effectively target perceptions of realistic threat but, conversely, are likely to be ineffective at targeting symbolic threat, as measured by adverse stereotypes, reconciliatory beliefs, or social stigma. The verdict as to which risk factor is more predictive of violent radicalisation is beyond the scope of this review. However, the authors propose that potential counter‐narrative interventions reflect the complexity of their prospective outcomes, and consider that an ineffective counter‐narrative, beyond having no effect, has the potential to have exacerbating effects.

The second policy implication relates to the use of specific techniques in counter‐narrative design. As mentioned, several counter‐narrative guidelines have been published by varying counter‐terrorism actors, and are freely available to the public.^15^ These guidelines advise on a range of techniques, from the use of counter‐arguments, to emotionally laden appeals. In 2014, the Quilliam Foundation published a practical guide to countering violent extremism online through, among other initiatives, counter‐messaging (Hussain & Saltman, 2014). They advised that governments, civil society, and the private sector work jointly to deliver effective counter‐messages that address the theological arguments put forth in violent extremist content. In particular, such efforts should “contextualise the scriptural references that are used by extremists” (p. 109), in attempts to undermine their credibility. Initiatives such as the “Ibaana” programme (a prison programme in which a trained chaplain challenges the theological arguments used by these prisoners to justify their extremist views; see HM Government, 2014) are an example of such strategies. The evidence from this review on the effectiveness of counter‐arguments is unclear. While one study saw success, the study was heavily informed by inoculation theory which posits that the creation of one's *own* counter‐arguments can increase resistance to persuasive influences. Evidence on the use of, for example, “theological arguments” to contradict violent extremist narratives is not sufficiently supported in this review.

Another technique which arises frequently in counter‐narrative guidelines is the use of persuasion. In 2013, the RAN, in collaboration with the ISD, published a detailed report on counter‐narratives, with recommendations for designing successful counter‐narrative campaigns using variety of techniques, including emotions, professional‐looking productions, and satire. Emotions, they report, are more important than evidence as facts and statistics can be dismissed while emotional appeals have “greater power” (p. 6); satire, they report, has historically played an effective role in undermining extremists such as the Ku Klux Klan while high quality productions “critical to legitimacy and appeal” (p. 6). The results from this review do not support this approach.

In this review, interventions which employed persuasive techniques, such as identification and transportation, demonstrated no effect at targeting perceived realistic threat (*d* = 0.08) or out‐group hostility (*d* = 0.00–0.18). In general, persuasive communication is a precarious methodology for manipulating attitudes or behaviour on contentious outcomes, such as threat or hostility. Although such techniques are well established in creating new attitudes (“response‐making”; see Berlo, 1960), as it has been suggested that counter‐narratives should be aimed at individuals “further along the path to radicalisation” (Briggs et al., 2013), there are several difficulties which may arise from the use of persuasive techniques. If an individual wants to maintain psychological consistency with their baseline attitudes (Wegener, Petty, Smoak, & Fabrigar, 2004), is not motivated to cognitively restructure (Festinger, 1957), or simply does not wish to engage with the appeal (Briñol, Rucker, Tormala, & Petty, 2004) such attempts will likely result in a “boomerang effect” (attitude change in the unintended direction; see Byrne & Hart, 2009), or no change at all.

It is well evidenced that persuasive techniques are used to entice vulnerable individuals into supporting, or perpetrating, acts of violence, rendering them fundamental components of terrorist communication (Braddock et al., 2016; Jowett et al., 2018). While persuasive communication certainly encompasses a spectrum of techniques (many of which do not function as a product of manipulation but, rather, of cognition, and how human beings process complex information), the logic of *relying* upon these methods in attempts to counter their effectiveness is counter‐intuitive. If the counter‐narrative is to become an evidence‐based tool for countering violent extremism, it should not need to employ the same techniques of those whom it intends to discredit.

### 7.2 Implications for Research

The reviewers make a number of recommendations for future research on counter‐narratives, specifically for violent radicalisation. These are broadly discussed under two central themes.

#### 7.2.1 Theory and techniques

At the beginning of this review, the counter‐narrative was introduced as an intervention informed by several theoretical frameworks while, at the same time, none at all. Theories such as the stereotype content model (Fiske et al., 2002), dual process models of persuasion (Green et al., 2002; Petty et al., 1986, 1999), models of narrative identity (Hammack, 2008) and inoculation theory (McGuire et al., 1962) have since emerged as the most common frameworks informing the studies in this review. However, this list is by no means exhaustive and the authors recommend the thoughtful consideration (and testing) of other theoretical frameworks also.

This leads to another recommendation in terms of the theory/theories informing the design of counter‐narratives. A lack of specificity, particularly in terms of techniques, surfaces as a challenge several times in this review. While the majority of studies were informed by a single theoretical perspective, some incorporated several frameworks, rendering it difficult to isolate the “active ingredients” in the intervention. Therefore, the authors recommend that researchers clearly specify the techniques they have used in their counter‐narrative, and avoid “mixing” different techniques (e.g., contact *and* counter‐stereotypical exemplars, see Section 6.1.2.1.1) in their interventions.

#### 7.2.2 Outcomes

As mentioned, many counter‐narrative strategies were excluded from this review as they measured outcomes related to intervention‐feasibility, rather than overall effectiveness at targeting violent radicalisation. As risk factors for violent radicalisation become more heavily supported by evidence, the authors recommend future research use validated measures of these constructs. Furthermore, it is suggested that using *single*, theoretically informed outcomes may provide more clarity, in terms of cause(s) and effect(s). While the authors of this review acknowledge that it can be difficult for such initiatives to show a “theory of change or impact” in this way (as suggested by Saltman et al., 2016, p. 25), this does not exempt those working in the field of counter‐terrorism, who intend to indicate “effectiveness”, from the standards applied to those working in other areas of behaviour change research.

In response to the first part of the review question, the authors have found little evidence that counter‐narratives are effective at targeting primary outcomes related to violent radicalisation. However, the scarcity of high‐quality studies measuring these outcomes means that this evaluation cannot, yet, be regarded as conclusive, and more research is needed.

## LEAD REVIEW AUTHOR

The lead author is the person who develops and co‐ordinates the review team, discusses and assigns roles for individual members of the review team, liaises with the editorial base and takes responsibility for the on‐going updates of the review.

## ROLES AND RESPONSIBILITIES

Information retrieval and coding: S. L. C., C. B. D., and K. C.

Risk of Bias assessment: S. L. C. and K. C.

To advise in statistical methods and contents: K. S. and D. O.

Statistical analysis and report writing: S. L. C. and K. M. S.

## SOURCES OF SUPPORT

For the original search and draft of this review, funding was provided by the Irish Research Council (IRC) Government of Ireland Postgraduate Scholarship. For the updated search, and completion of this review, funding was provided by the Department of Homeland Security (DHS).

## DECLARATIONS OF INTEREST

None of the researchers involved in the team present any conflict(s) of interest.

## PLANS FOR UPDATING THE REVIEW

Sarah L. Carthy will be responsible for updating the review every five years.

## AUTHORS' RESPONSIBILITIES

By completing this form, you accept responsibility for maintaining the review in light of new evidence, comments and criticisms, and other developments, and updating the review at least once every five years, or, if requested, transferring responsibility for maintaining the review to others as agreed with the Coordinating Group. If an update is not submitted according to agreed plans, or if we are unable to contact you for an extended period, the relevant Coordinating Group has the right to propose the update to alternative authors.

## PUBLICATION IN THE CAMPBELL LIBRARY

The Campbell Collaboration places no restrictions on publication of the findings of a Campbell systematic review in a more abbreviated form as a journal article either before or after the publication of the monograph version in *Campbell Systematic Reviews*. Some journals, however, have restrictions that preclude publication of findings that have been, or will be, reported elsewhere, and authors considering publication in such a journal should be aware of possible conflict with publication of the monograph version in *Campbell Systematic Reviews*. Publication in a journal after publication or in press status in *Campbell Systematic Reviews* should acknowledge the Campbell version and include a citation to it. Note that systematic reviews published in *Campbell Systematic Reviews* and co‐registered with the Cochrane Collaboration may have additional requirements or restrictions for co‐publication. Review authors accept responsibility for meeting any co‐publication requirements.

**I understand the commitment required to update a Campbell review, and agree to publish in the Campbell Library. Signed on behalf of the authors**:

**Table:** 

**Form completed by:** Sarah Carthy	**Date:** 6th January 2020

## Supporting information

Supporting information

Supporting information

Supporting information

## Footnotes

1 Single‐issue politics refer to movements which campaign on only one issue. These include issue such as animal rights, cannabis legalisation and reform of copyright and patent law (“pirate parties”).

2 The Global Terrorism Database (GTD) estimates that between 2007 and 2018, the average number of annual deaths caused by terrorist attacks was 21,000 (from its lowest of 7,827 in 2010, to a record high of 44,490 in 2014). This data was gathered by the National Consortium for the Study of Terrorism and Responses to Terrorism (START) at the University of Maryland.

3 Due to its variable nature, as well an overall low rate of offending, risk assessment for violent extremism or terrorism (ERG22 and VERA version 2) is not considered best‐practice. These tools were therefore employed cautiously, and in tandem with a suitable evidence base.

4 Although the secondary outcomes are summarised as part of the methods section, the process of identifying risk factors occurred *after* the identification of studies to be included in the review.

5 These papers, although published within the parameters of the first search, were likely uploaded within the parameters of the second.

6 Although the protocol specifically outlined that interrupted time series (ITS) designs would not be included in the review, two studies using these designs were nonetheless included (Frischlich et al., 2018). Their inclusion was justified as they offered insight into psychological measurement for violent radicalisation. The risk of bias for these studies was high and, therefore, moderator analyses were conducted to determine the impact of potential bias(es).

7 The dominant narrative among the Caucasian‐American, university sample was determined to be that African‐Americans are hostile, criminal, lazy, drug users, and aggressive. These attributes (among others) were identified through two pilot studies with comparable samples. As this data had been provided, the “stereotypical African‐American” condition was not viewed as an exacerbating dominant narrative, as was the case with Saleem et al. (2015).

8 Narratives of civil restrictions are purported in justification of settler violence in the West Bank (Ginges, Atran, Sachdeva, & Medin, 2011); see Friedman (1986) and Luz (2002) for descriptions of typical arguments purported by violent extremist groups such as the Jewish Underground Movement and Terror Against Terror.

9 Alhabash and Wise (2012, 2015) conducted two randomised control trials, with a within‐subjects factor. As explained in section 5.1.3 (“dominant narratives”), only data pertaining to the Palestinian president condition were included in this review and, as such, the study is presented, and assessed, as a nonrandomised study.

10 The mean age of participants was calculated from the following studies: Bilewicz & Jaworska, (2013, *M*_age_ = 16.84), Cohen et al. (2015, *M*_age_ = 25.24), Banas and Richards (2017, *M*_age_ = 19.71), Riles et al. (2017, *M*_age_ = 19.47), Alhabash and Wise (2012, *M*_age_ = 20; 2015, *M*_age_ = 20.80), Bruneau et al. (2017, *M*_age_ = 33.65; 2017b, *M*_age_ = 34.95; 2017c, *M*_age_ = 34.3) and Čehajić‐Clancy and Bilewicz, (2017, *M*_age_ = 20.09; 2017b, *M*_age_ = 20). The standard deviation was only provided by five studies so could not be confidently determined.

11 Calculated from the following studies: Gonsalkorale et al. (2010, 78% female), Cernat (2001, 33% female), Alhabash and Wise (2012, 74% female), Bilewicz & Jaworska, (2013, 69.1% female), Cohen et al. (2015, 75% female), Saleem et al. (2015, 49% female), Banas and Richards (2017, 43% female), Bruneau et al. (2017, 47% female; 2017b, 50.15% female; 2017c, 43.35% female), Riles et al. (2018, 60% female), and Čehajić‐Clancy and Bilewicz, (2017, 61% female).

12 1. (Alhabash and Wise, 2012); 2. (Alhabash and Wise, 2015); 3. (Banas and Richards 2017); 4. (Bilewicz & Jaworska, 2013); 5–8. (Bruneau et al., 2017); 9. (Čehajić‐Clancy & Bilewicz, 2017); 10. (Cernat, 2001); 11. (Cohen et al., 2015); 12. (Gonakorale et al., 2010); 13. (Riles et al., 2018); and 14. (Saleem et al., 2015).

13 Note: all studies conducted by Bruneau et al. (2017) used independent samples. This sensitivity analysis is therefore not to be interpreted as a treatment of multiple or “duplicate” publication bias (as described by Gøtzsche, 1989).

14 Note. The UK Home Office and Public Safety Canada were contacted, requesting potentially relevant studies for inclusion in this review. However, the reviewers suggest that, given the timing of the review (in tandem with Brexit negotiations, changes in UK leadership as well as parliamentary elections), the full breath of the counter‐narrative activities in the Commonwealth may not have been exhausted.

15 In 2016, Tuck and Silverman published “The Counter‐Narrative Handbook”; in 2017, the RAN published guidelines for effective alternative and counter‐narrative campaigns (GAMMMA+).
