# Supplementary material for: Counter‐narratives for the prevention of violent radicalisation: A systematic review of targeted interventions
Source: Campbell Syst Rev. 2020 Aug 12;16(3):e1106. doi: 10.1002/cl2.1106 (PMC8356325; doi:10.1002/cl2.1106)
Supplement: Supplementary file 1 — Supporting information [file CL2-16-e1106-s003.docx]

# Appendix A. Additional Tables

## Table A1. Data extraction table

Table A1 is included as a separate, supplementary file.

## Table A2. Search terms

| Table A2. Search Terms | | |
| --- | --- | --- |
| **Search domain** | **Topical domain** | **Key Words** |
| Title or Abstract | Intervention *(‘counter-narrative’)* | (Alter-messaging OR Alternative framing OR Anti-messaging OR anti-radicalism message OR anti-radicalization message OR Anti-terrorist campaign OR Anti-violence campaign OR Argument scrutiny OR attitude-change OR Citizen messenger OR Common narrative OR Contesting narratives OR Countering ideological support for extremism OR Counter analogy OR Counter-argument OR Counter-attitudinal OR Counter-campaign OR Counter example OR Counter-ideological OR Counter-message OR Counter-messaging campaign OR Counter-messaging intervention OR Counter-messaging interventions OR Counter-messaging strategy OR Counter-messenger OR Counter-narrative OR Counter-narrative campaign OR Counter-narrative message OR counter-radicalisation OR counter-recruitment OR counter-speech OR counter-strategy OR countering campaign OR countering materials OR ideological counterpoint OR strategic narrative OR narrative intervention OR narrative transportation OR public diplomacy OR rhetorical education OR Security narrative OR strategic communication OR Ideological response) |
| Title or Abstract AND Full Text | Research area (*‘counter-terrorism’*) | (Anti-colonialism OR Anti-imperialist OR Anti-terror OR Anti-terrorism OR Battle of Ideas OR Conflict OR Conflict resolution OR Counter-terrorism OR counter-radicalization OR Countering Violent Extremism OR CVE OR Deradicalisation OR Deradicalization OR De-radicalisation OR De-radicalization OR Disengagement OR Extremism OR Ideological distortion OR ideological distortions OR Ideological battle OR Ideological War OR Indoctrination OR intellectual activist OR Islamic terrorism OR Islamist terrorism OR militant activist OR Online radicalisation OR Recruit OR Radical OR Radical group OR Radical movement OR Radicalisation OR Radicalism OR Security OR Terrorism OR Terrorist action OR violence OR Violent Extremism OR War OR War of ideas OR Terrorist threat OR Terrorist incident OR Terrorist ideology OR Terrorist sympathiser OR Violent extremism online OR Violent extremist ideology OR Violent extremist message) |
| Full Text | Problem *(‘terrorist narrative’)* | (Alternative historical accounts OR Extremist propaganda OR Extremist sympathiser OR Audio-visual production OR Branding OR Collective memory OR Digital communication OR Extremism online OR Extremist argument OR Extremist content online OR Extremist ideology OR Extremist message OR Extremist narrative OR Anti-American OR anti American OR Anti-American rhetoric OR Global narrative OR Ideology, Ideological influence OR ideological legitimization OR Ideological message OR Ideological support OR Indoctrination OR Islamist extremist narrative OR Islamist ideology OR Jihadi ideologues OR Justifications for violence OR Legitimacy of terrorism OR Local narrative OR Master Narrative OR Media communication OR Message OR Message manipulation OR Meta-narrative OR Misinformation OR Misinformation online OR Narrative OR narrative criminology OR Narrative transformation OR Observational Argument OR Online extremism OR opinion change OR Persuasion OR Persuasive communication OR Personal narrative OR Persuasive strategies OR Political idea OR Political strategy OR Propaganda campaign OR radicalism OR radical ideology OR radical narrative OR radical perspective OR radical perspectives OR radical theorist OR radical worldview OR recruitment narrative OR Religio-ideological OR Religious justification OR Rhetoric OR Rhetorical tactics OR Rhetorical terrorism OR Rhetorical vision OR Social-influence OR Statements OR Terror OR recruitment strategies) |

## Table A3. Databases

| **Table A3.** Search Methods used in Databases | | | |
| --- | --- | --- | --- |
| **Database** | | **Area** | **Search method** |
| **Web of Science** | | *Science* | 1 “Intervention” TITLE and “Research Area” TOPIC and “Problem” TOPIC |
|  |  |  | 2 “Intervention” TITLE and “Research Area” TITLE and  “Research Area” TOPIC and “Problem” TOPIC |
|  |  |  | 3 “Intervention” TITLE and “Research Area” TOPIC and  “Problem” TITLE |
|  |  |  | 4 “Intervention” TITLE and “Research Area” TITLE and “Problem” TITLE |
|  | | | 5 “narrative” TITLE and “experiment” or “randomised control trial” or “randomized control trial” or “RCT” TOPIC and “violence” OR “extremism” OR violent extremism |
| **PsycInfo** | | *Psychology* | “Intervention” TITLE or ABSTRACT and“Research Area” ALL FIELDS and “Problem” ALL FIELDS |
|  |  |  | “Intervention” TITLE or ABSTRACT and “Research Area” TITLE or ABSTRACT and “Research Area” ALL FIELDS and “Problem” ALL FIELDS |
|  |  |  | “Intervention” TITLE or ABSTRACT and “Research Area” ALL FIELDS and “Problem” TITLE or ABSTRACT |
|  |  |  | *Shortened* “Intervention” TITLE or ABSTRACT and *Shortened* “Research Area” TITLE or ABSTRACT and *Shortened* “Problem” TITLE or ABSTRACT |
| **Scopus** | | *Science* | *Shortened* “Intervention” ALL and *Shortened* “Research Area” ALL and *Shortened* “Problem” ALL |
|  |  |  | “Intervention” TITLE-ABS-KEY and *Shortened “*Research Area” ALL and *Shortened* “Problem” ALL |
|  |  |  | “Intervention” TITLE-ABS-KEY and *“*Research Area” ALL and *Shortened* “Problem” ALL |
|  |  |  | “Intervention” ALL and *“*Research Area” ALL and *Shortened* “Problem” ALL |
|  |  |  | “Intervention” ALL and *“*Research Area” ALL and “Problem” ALL |
| **Zetoc** | *Reports* | | (keywords) “terrorism” and “narrative” |
| **Worldwide Political Science Abstracts** | Political Science | | “Intervention” ABSTRACT and “Research Area” ABSTRACT and “Problem” ABSTRACT and (excl. book reviews and news)  *Shortened* “Intervention” DOCUMENT TITLE |
|  |  |  | *Shortened* “Intervention” ABSTRACT and “Research Area” ABSTRACT and “Problem” ABSTRACT |
| **Columbia International Affairs Online** | Politics | | (keywords) “terrorism” and “narrative” and “prevent” |
| **Applied Social Sciences Index & Abstracts (ASSIA)** | Social Science | | *Shortened* “Intervention” DOCUMENT TITLE or “Intervention” ABSTRACT or “Research Area” DOCUMENT TITLE and “Problem” ABSTRACT |
|  |  |  | *Shortened* “Intervention” DOCUMENT TITLE and “Research Area” DOCUMENT TITLE and “Problem” ABSTRACT |
|  |  |  | (keyword) “terrorism” ABSTRACT and *Shortened* “Intervention” ABSTRACT |
|  |  |  | *Shortened “*Research Area” ABSTRACT and *Shortened* “Intervention” ABSTRACT |
|  |  |  | “Intervention” DOCUMENT TITLE and  “Research Area” ABSTRACT and  “Problem” ALL  16 (2015-2020) |
| **EThOS** | | Unpublished (Doctoral Theses) | (keywords) ‘terrorism’ or ‘radicalisation’ or ‘radicalization’ and ‘narrative’ |
| **NCJRS Abstracts Database** | | Criminal | (keywords) ‘terrorism’ or ‘radicalisation’ or ‘radicalization’ and ‘narrative’  “COUNTERING VIOLENT EXTREMISM” |
| **Directory of Open Access Journals (DOAJ)** | | Science | (keywords) “violent extremism” or “violent extremist” and “radicalization” or “radicalisation”  “counter-narrative” OR “counternarrative” |
| **Hedayah (Canadian Network for Research on Terrorism, Security and Society)** | | Security | (keywords) “violent extremism” or “violent extremist” and “radicalization” or “radicalisation” |
| **SAGE** | | Science | [[Title counternarrative] OR [Title counter-narrative]] AND [All experiment] |

## Table A4. Professional agencies

| **Table A4.** Hand searches of Research and Professional Agencies’ Outputs and Publications | |
| --- | --- |
| **Agency** | **Location** |
| **Department of Homeland Security (DHS)** | *Washington, USA* |
| **European Consortium for Political Research (ECPR)** | *Colchester, United Kingdom* |
| **European Expert Network on Terrorism Issues (EENet)** | *German Federal Criminal Police Office* |
| **Institute for Strategic Dialogue (ISD)** | *London, United Kingdom*  *operational responses to the rising challenges of violent extremism and inter-communal conflict* |
| **International Centre for Counter-Terrorism (ICCT)** | *The Hague, Netherlands* |
| **International Centre for the Study of Radicalisation (ICSR)** | *London, United Kingdom* |
| **International Police Association (IPA)** | *International* |
| **Public Safety Canada** | *Ottawa, Canada* |
| **UK Home Office** | *London, United Kingdom* |

## Table A5. List of experts

| **Table A5.** List of Experts. | |
| --- | --- |
| **Name** | **Affiliation** |
| Ajmal Aziz | Department of Homeland Security |
| Neil Aggarwal | New York State Psychiatric Institute |
| JM Berger | International Centre for Counter Terrorism |
| Kurt Braddock | Pennsylvania State University |
| Marco de Swart | International Centre for Counter Terrorism (ICCT) |
| Paul Gill | University College London |
| John Horgan | Georgia State University |
| Niall O Dochartaigh | National University of Ireland, Galway |
| Alex Schmid | International Centre for Counter Terrorism (ICCT) |
| Anne Speckhard | International Center for the Study of Violent Extremism (ICSVE) |
| Judith Tinnes | Perspectives on Terrorism |

## Table A6 Conceptual papers and books

| **Table A6.** Conceptual Papers and Books. |
| --- |
| **Reference** |
| Briggs, R., & Reves, S. (2013). Review of programs to counter narratives of violent extremism: What works and what are the implications for government? : *Institute for Strategic Dialogue.* |
| Goodall Jr, H. (2010). *Counter-narrative: How progressive academics can challenge extremists and promote social justice*. New York: Routledge. |
| Ferguson, K. (2016). Countering violent extremism through media and communication strategies: *The Partnership for Conflict, Crime and Security Research.* |
| Halverson, J. R., Corman, S.R., & Goodall Jr, H. L. (2011). *Master Narratives of Islamist Extremism*: Palgrave Macmillan US. |
| Tinnes, J. (2016). Bibliography: Terrorism and the Media (including the Internet) (Part 3). |
| Tinnes, J. (2014). Bibliography: Terrorism and the media (including the internet) (part 2). *Perspectives on Terrorism, 8*(6). |
| Tinnes, J. (2014). Bibliography: Terrorism and the Media (including the Internet) (Part 2). *Perspectives on Terrorism, 8*(6). |
| Tinnes, J. (2013). Literature on Terrorism and the Media (including the Internet): an Extensive Bibliography. *Perspectives on Terrorism, 7*(1). |

## Table A7. Study outcomes and associated risk factors

| **Table A7.** Study outcomes and their associated risk factors. | | | | | | | |
| --- | --- | --- | --- | --- | --- | --- | --- |
| **Study** | | **Outcome** | | **Sample item(s)** | | **Risk Factor** | |
|  |  |  |  |  |  | **Sub-category** | **Overall** |
| 1 | Cernat (2001) | Stereotype dimensions | | *Hungarians are:* aggressive*,* extreme etc. | | Symbolic threat^1^ | Perceived group threat |
|  |  | Evaluations | | *Feelings towards Hungarians:*  admiration, respect, attraction, joy, disgust etc. | | Explicit bias | Out-group hostility |
| 2 | Kendrick et al. (2004) | *Rate your feelings towards:* The US government. | | | | Realistic threat^3^ | Perceived group threat |
|  |  | *Rate your feelings towards:* How Muslims are treated in the US | | | |  |  |
|  |  | *Rate your feelings towards:* The US people. | | | | Explicit bias | Out-group hostility |
| 3 | Ramasubramanian et al. (2007) | Hostile feelings | | *[Feelings towards African-Americans]:* fear, anger, hostility etc. | | Realistic threat^3^ | Perceived group threat |
|  |  | Feeling Thermometer | | *[Feelings towards African-Americans]:* from 0◦ (very negative) to 100◦ (very positive) | | Explicit bias | Out-group hostility |
| 4 | Gonsalkorale et al.(2010) *Study 2* | Implicit Association Task (IAT) | | *Participants used two keys to categorize 12 target images (six Black faces, six White faces) and 16 evaluative words (8 pleasant, 8 unpleasant).* | | In-group favouritism/out-group hostility | |
| 5 | Alhabash et al. (2012) | National attitudes | | *[Palestinians]:* “want peace”, “are responsible for violence”, “are democratic” etc. | | Symbolic threat^1^ | Perceived group threat |
|  |  | Affective Misattribution Procedure (AMP) | | *Participants were exposed to an image of a Palestinian, followed by a neutral (usually Chinese) image which they then rated for “valence” (i.e. negative – positive).* | | Implicit bias | Out-group hostility |
| 6 | Bilewicz et al. (2013) | Feeling Thermometer (Alwin, 1997) | | *[Feelings towards Polish people]:* from 0◦ (very negative) to 100◦ (very positive) | | Explicit bias | Out-group hostility |
|  |  | Perceived similarity to the self (reversed) | | *[shared with Polish people]: “*common interests” “common experiences” | | Symbolic threat^2^ | Perceived group threat |
| 7 | Garagosov (2013) | Implicit association task (IAT) | | *Participants used two keys to categorize images of the former Armenian president (Serzh Sargsyan) and evaluative words* | | Implicit bias | Out-group hostility |
| 8 | Alhabash et al.(2015) | Explicit stereotypes. | | *[Palestinians are]:*  dirty, loyal, cruel etc. | | Symbolic threat^1^ | Perceived group threat |
|  |  | Affective Misattribution Procedure (AMP) | | *Participants were exposed to an image of a Palestinian, followed by a neutral (usually Chinese) image which they then rated for “valence” (i.e. negative – positive).* | | Implicit bias | Out-group hostility |
| 9 | Cohen, Tal-Or et al. (2015) *Study 1* | Post-exposure attitudes | | *[Arab students]* “It should be forbidden for [Arab students] to demonstrate in the heart of the campus”; “each and every citizen has the right to express his or her opinions everywhere, including on campus” etc. | | Realistic threat^4^ | Perceived group threat |
| 10 | Saleem et al. (2015) *Experiment 3* | Perceptions of Muslims as aggressive | | *[Muslims are]:* dangerous, violent etc. | | Realistic threat^3^ | Perceived group threat |
|  |  | Support for civil restrictions for Muslim Americans | | *[Muslims Americans]:* should not be allowed to vote, should have to do annual security clearance checks with government agencies etc. | |  |  |
|  |  | Support for military action in Muslim countries | | E.g. “I would support the use of U.S. military to reduce the influence of Islam on other countries” | | Radical belief system | |
| 11 | Banas et al. (2017) | Conspiratorial attitudes | | *[The position advocated in the anti-government message^1^ was]* foolish / wise, bad / good, unfavourable / favourable, unacceptable / acceptable etc. | | Realistic threat^3^ | Perceived group threat |
| 12 | Bruneau et al. (2017) | *Study 1* | Palestinian violence | | E.g. “*[Palestinians]* are much more violent than other groups” | Realistic threat^3^ | Perceived group threat |
|  |  |  | Feeling thermometer (Haddock, Zanna, & Esses, 1993) | | *[Feelings towards Palestinians]:* from 0◦ (very negative) to 100◦ (very positive) | Explicit bias | Out-group hostility |
| 13 |  | *Study 2* | Palestinian violence | | *See Study 1.* | Realistic threat^3^ | Perceived group threat |
|  |  |  | Feeling thermometer (Haddock et al., 1993) | | *See Study 1.* | Explicit bias | Out-group hostility |
| 14 |  | *Study 3* | Palestinian violence | | *See Study 1.* | Realistic threat^3^ | Perceived group threat |
|  |  |  | Feeling thermometer (Haddock et al., 1993) | | *See Study 1.* | Explicit bias | Out-group hostility |
| 15 | Čehajić-Clancy et al.(2017) | *Study 1* | Belief in reconciliation | | *I believe/don’t believe that [Towards Bosniaks, Serbs, or Croats]:* “will ever be able to live together in peace” , “can cooperate together” “can build a country together” etc. | Symbolic threat^1^ | Perceived group threat |
|  |  |  | Forgiveness | | “I am ready to forgive *[Bosniaks, Serbs, or Croats]* for things that they have done during the war”, “I could never forgive the committed crimes” etc. | Explicit bias | Out-group hostility |
| 16 |  | *Study 2* | Belief in reconciliation | | *See Study 1.* | Symbolic threat^1^ | Perceived group threat |
|  |  |  | Intergroup anxiety | | *[Feelings towards Bosniaks, Serbs, or Croats]:* trust, comfort, confidence etc. | Out-group hostility | |
| 17 | Frischlich et al. (2018) | *Study 1* | Extremist attitudes | | Agreement with*: [RWEX and ISEX propaganda]* | Radical belief system | |
| 18 |  | *Study 2* | Extremist attitudes | | *See Study 1.* |  |  |
| 19 | Riles et al. (2018) | Social Stigma (Link et al., 1997, p. 181; Smith, 2012) | | “Most people would think less of a person who is a *[Muslim]*” , “Most employers will hire a *[Muslim person]* if he or she is qualified for the job”, "Most people believe that *[Muslims]* cannot be trusted” | | Symbolic threat^2^ | Perceived group threat |
|  |  | Social Distance | | “Could you see yourself renting a room in your home to *[a Muslim person]*?”, “Could you see yourself spending an evening socializing with *[a Muslim person]*?” etc. | | Realistic threat^4^ |  |
| ^1^Perceived differences in values and/or motives ^2^Perceived group differences ^3^To one’s physical safety ^4^To one’s existence | | | | | | | |

## Table A8. Sensitivity analysis summary table

| **Table A8a.** Sensitivity analysis testing individual study-changes for analysis 1 | | | | | | | |
| --- | --- | --- | --- | --- | --- | --- | --- |
| Analysis | Study | Decision | | SMD | 95% CI | p | Heterogeneity |
| *Original analysis* | Cernat (2001) | Overall average across outcomes | Stereotype dimensions (aggression + extremism / 2) + negative evaluations (Hungarians) / 2 | -.39 | -0.55 to -0.24 | .000 | (χ2 = 19.38 [p <.02], I 2 = 54%, τ2 = 0.03) |
| *Sensitivity analysis* | Cernat (2001) | Most relevant outcome (no reliability analysis provided) | Stereotype dimensions (aggression + extremism / 2) | -.39 | -0.55to 0.24 | .000 | (χ2 = 19.41 [p <.02], I 2 = 54%, τ2 = 0.03) |
| *Result* | No change | | | | | | |
| *Original analysis* | Bilewicz et al. (2013) | Overall average across outcomes | Perceived similarity to the self + feeling thermometer / 2 | -.39 | -0.55 to -0.24 | .000 | (χ2 = 19.38 [p <.02], I 2 = 54%, τ2 = 0.03) |
| *Sensitivity analysis* | Bilewicz et al. (2013) | Most valid (α = .92) | Perceived similarity to the self | -.39 | -0.54 to -0.24 | .000 | (χ2 = 18.73 [p <.03], I 2 = 52%, τ2 = 0.03) |
| *Result* | No change | | | | | | |
| *Original analysis* | Saleem et al. (2015) | Overall average across outcomes | (Violent perceptions + civil restrictions / 2) + military action / 2 | -.39 | -0.55 to -0.24 | .000 | (χ2 = 19.38 [p <.02], I 2 = 54%, τ2 = 0.03) |
| *Sensitivity analysis* | Saleem et al. (2015) | Most valid outcome (α = .92) | Civil restrictions | -.39 | 0.55 to -0.22 | .000 | (χ2 = 22.81 [p <.01], I 2 = 61%, τ2 = 0.04) |
| *Result* | No change | | | | | | |
| *Original analysis* | Bruneau et al. (2017a) | Overall average across outcomes | Palestinian violence + feeling thermometer /2 | -.39 | -0.55 to -0.24 | .000 | (χ2 = 19.38 [p <.02], I 2 = 54%, τ2 = 0.03) |
| *Sensitivity analysis* | Bruneau et al. (2017a) | Most valid outcome | Palestinian violence | -.40 | -0.56 to -0.25 | .000 | (χ2 = 19.86 [p <.02], I 2 = 55%, τ2 = 0.03) |
| *Result* | Slight increase in effect size | | | | | | |
| *Original analysis* | Bruneau et al. (2017b) | Overall average across outcomes | Palestinian violence + feeling thermometer /2 | -.39 | -0.55 to -0.24 | .000 | (χ2 = 19.38 [p <.02], I 2 = 54%, τ2 = 0.03) |
| *Sensitivity analysis* | Bruneau et al. (2017b) | Most valid outcome (α = .87) | Palestinian violence | -.39 | -0.55 to -0.24 | .000 | (χ2 = 19.36 [p <.02], I 2 = 54%, τ2 = 0.03) |
| *Result* | No change | | | | | | |
| *Original analysis* | Bruneau et al. (2017c) | Overall average across outcomes | Palestinian violence + feeling thermometer /2 | -.39 | -0.55 to -0.24 | .000 | (χ2 = 19.38 [p <.02], I 2 = 54%, τ2 = 0.03) |
| *Sensitivity analysis* | Bruneau et al. (2017c) | Most valid outcome (α = .93) | Palestinian violence | -.40 | -0.56 to -0.25 | .000 | (χ2 = 18.80 [p <.03], I 2 = 52%, τ2 = 0.03) |
| *Result* | Slight increase in effect size | | | | | | |
| *Original analysis* | Riles et al. (2018) | Overall average across outcomes | Social stigma + social distance / 2 | -.39 | -0.55 to -0.24 | .000 | (χ2 = 19.38 [p = .02], I 2 = 54%, τ2 = 0.03) |
| *Sensitivity analysis* | Riles et al. (2018) | Most valid outcome (α = .92) | Social distance | -.58 | -0.94 to -0.22 | .001 | (χ2 = 104.60 [p < .000], I 2 = 91%, τ2 = 0.29) |
| *Result* | Moderate increase in effect size | | | | | | |

# Appendix B. Figures

## Figure B1. Review Timeline.

| **Figure B1.** Review Timeline. | | | |
| --- | --- | --- | --- |
| **2016** | **May** |  | Development of search terms  Title document  Protocol document (draft) |
|  | **June** |  |  |
|  | **July** |  |  |
|  | **August** | Title Registration submitted | 1) Targeted keyword search  2) Hand searches |
|  | **September** |  |  |
|  | **October** |  | 3) Reference lists and; 4) Contacting experts |
|  | **November** | Reviewer comments received;  Title Registration resubmitted | 1) Title screening  2) Abstract screening  3) Full title screening |
|  | **December** |  |  |
| **2017** | **January** |  |  |
|  | **February** |  |  |
|  | **March** |  | Second screening (CD) |
|  | **April** | Reviewer comments received;  Title Registration resubmitted | Data extraction  Write-up  First draft completed |
|  | **May** |  |  |
|  | **June** |  |  |
|  | **July** |  |  |
|  | **August** |  |  |
|  | **September** | Title Registered |  |
|  | **October** |  | Review revised |
|  | **November** | Protocol submitted |  |
|  | **December** |  |  |
| **2018** | **January** | Reviewer comments received  Protocol resubmitted | Final draft completed |
|  | **February** | Protocol resubmitted |  |
|  | **March**  **April**  **May**  **June**  **July**  **August** |  |  |
|  |  |  |  |
|  |  |  |  |
|  | **September** | Protocol published |  |
|  | **October**  **November**  **December**  **January**  **February**  **March** |  |  |
|  |  |  |  |
| **2019** |  |  | Decision made to update review |
|  | **April** |  | 1) Targeted keyword search |
|  | **May** |  |  |
|  | **June** |  | 1) Title screening  2) Abstract screening  3) Full title screening |
|  | **July** |  |  |
|  | **August** |  | Second coder (search) (KC)  Data extraction (second coder KC) |
|  | **September** |  | Write-up |
|  | **October** |  | Final draft completed |

# Appendix C: Coding Categories

## Appendix C.1 Exclusion Criteria

#### Appendix C.1 Exclusion criteria

a) Exclusion criteria for title and abstract screening

1. Exclude Duplicate

a) Publications which match another title in exactly (i.e. title, author and year) should be excluded.

1. Exclude Language
2. Publications not published in English should be excluded.
3. Exclude Publication Type
4. Publications that do not report first-hand empirical findings (e.g. government reports, newspaper articles and minutes of meetings) should be excluded.
5. Exclude non-Intervention
6. Publications which are not exposing participants to an intervention should be excluded.
7. Publications which are not adopting an experimental design (e.g. RCT or factorial design) should be excluded.
8. Exclude Narrative vs. Counter-Narrative
9. Publications which do not challenge an existing narrative should be excluded.
10. Exclude Unrelated to Violent Radicalisation
11. Publications which do not measure outcomes related to violent radicalisation or target risk factors for violent radicalisation should be excluded.

## Appendix C.2 Inclusion Criteria

#### Appendix C.2 Inclusion criteria

b) Inclusion criteria for title and abstract screening

1. Counter-Narrative intervention
2. Publications exposing participants to an intervention which can be operationally defined as a counter-narrative should be included.
3. The counter-narrative intervention may be therapeutic (delivered following exposure to an existing narrative).
4. The counter-narrative intervention may be preventative (delivered before exposure to a narrative ‘to-be-countered’)
5. Evidence of existing narrative
6. Publications which show evidence of an existing narrative or a narrative ‘to-be-countered’ should be included.
7. The narrative may be determined by baseline/pre-test scores.
8. The narrative may be experimentally introduced.
9. The narrative among the population may be empirically evident in a different study.
10. The narrative may be evident in the sample due considerable evidence to be weighed by the initial and second coder.
11. Outcomes
12. Publications which measure outcomes related to violent radicalisation should be included.
13. Outcomes may be primary outcomes related to violent radicalisation (e.g. engagement in violent extremism or providing support to violent extremist groups).
14. Outcomes may be secondary outcomes related to violent radicalisation (e.g. adversarial stereotypes, outgroup feelings or attitudes towards violence).

- Secondary outcomes, in particular, must have justification for inclusion as outlined in the outcomes justifications table (Table X).

1. Design
2. Publications adopting an experimental design where at least one of the independent variables involves comparing a counter-narrative to a control or comparison exposure should be included. These may include:
3. Randomised Control Trials (RCT) whereby participants are randomly assigned to experimental or control conditions (e.g. two-group between-subjects design).
4. Factorial designs, with more than one independent variable (e.g. pre-post as a within-subjects variable, and exposure (e.g. present/absent) as a between-subjects variable.
5. Single group pre- and post-test studies which collect longitudinal data at baseline and end-line

## Appendix C.3 Coding Schemes

| a) Descriptive coding scheme | | | | |
| --- | --- | --- | --- | --- |
| **Study and year** | | | Alhabash and Wise (2012) | |
| **Participants** | *N*  *Gender*  *Ethnicity/nationality* | | Pro-Israeli participants (*N* = 68; 74% female; mean age = 20) recruited from an introductory undergraduate advertising course at an American university. | |
| **Dominant-narrative (DN)** | | | Anti-Palestinian narrative. | |
| **Counter-narrative (CN)** | | | Role play from the Palestinian perspective to initiate self-persuasion | |
| **Counter-narrative techniques** | | | Single group pre/post-test design | |
|  | *Theory* | | The study exposed participants to a video game which was designed according to procedural rhetoric (Bogost, 2006; 2007) whereby message autonomy (the players capacity to exhibit agency over decisions in the game), integration (the embeddedness of the object of persuasion) and goal overlap (the level of overlap between the learning goal and the tactical goals of the game) are used to encourage self-persuasion. | |
|  | *Intervention* | | Participants were instructed to play the role of the Palestinian president with the ultimate tactical goal of achieving peace. | |
|  | *Risk factors* | | The counter-narrative targeted negative attitudes towards Palestinians as a risk factor for ethnic extremism. | |
|  | *Logic* | | The intervention followed the logic that through integrating persuasive elements into a video game, participants with previous negative attitudes towards Palestinians would report lower negative implicit attitudes, reducing their risk of engaging in ethnic extremism against Palestinians | |
|  |  | |  |  |
| **Outcomes (operationalised)** | | | Explicit attitudes towards the adversary (Palestinians). | |
| **Outcomes (measured)** | | | Explicit attitudes were measured with a validated national attitudes scale which: measured 7 statements about each national group regarding (1) favourability; (2) sympathy; (3) belief about the national group’s intention for peace; (4) intentionally targeting civilians from the other side; (5) being democratic; (6) being responsible for the violence and, (7) having the right to sole control over the city of Jerusalem. | |
| b) Coding scheme representing effects of the intervention | | | | |
| **Study and year** | | Alhabash and Wise (2012) | | |
| **Outcome** | | Explicit attitudes towards an adversary measured using a validated national attitudes scale (α = .71). Higher scores = higher favourability towards the adversary (Palestinians). | | |
| **Results** | | *Control condition*  Pre-test | | *Experimental condition*  Post-test |
|  | | *N =* 35 | | *N =* 35 |
|  |  | *M =* 3.77 | | *M =* 4.03 |
|  |  | *SD =* 0.47 | | *SD =* 0.49 |

# Appendix D. Risk of bias

## Appendix D.1 Risk of bias assessment

| **Table D1.** Risk of bias assessment. | | | | | | | | | | | | |
| --- | --- | --- | --- | --- | --- | --- | --- | --- | --- | --- | --- | --- |
|  | **Cernat(2001)** | **Ramasubramanian & Oliver (2007)** | **Gonsalkorale, Allen, Sherman & Klauer (2010)** | **Bilewicz &**  **Jaworska (2013)** | **Garagozov (2013)** | **Cohen, Tal-Or & Mazor- Tregerman (2015)**  ***Study 1*** | **Saleem, Prot, Anderson & Lemieux (2015)**  ***Study 3*** | **Banas & Richards (2017)** | **Bruneau, Lane & Saleem (2017a)**  ***Study 1*** | **Bruneau, Lane & Saleem (2017b)**  ***Study 2*** | **Bruneau, Lane & Saleem (2017c)**  ***Study 3*** | **Riles, Funk & David (2018)** |
| **1. Was the allocation sequence adequately generated?** | Low risk. | Low risk. | Low risk. | Low risk. | Unclear. | Low risk. | Low risk. | Low risk. | Low risk. | Low risk. | Low risk. | Low risk. |
| **2. Was the allocation adequately concealed?** | Low risk. | Unclear. | Unclear. | Low risk. | Unclear. | Unclear. | Unclear. | Unclear. | Low risk. | Low risk. | Low risk. | Low risk. |
| **3. Were baseline outcome measurements similar?** | Unclear. | Low risk. | Unclear. | Unclear. | High risk. | Unclear. | Unclear. | Unclear. | Unclear**^1^**. | Low risk. | Low risk. | Unclear. |
| **4. Were baseline characteristics similar?** | Low risk. | Low risk. | Low risk. | Low risk. | High risk**^2^** | Low risk. | Low risk. | Low risk. | Low risk. | Low risk. | Low risk. | Low risk |
| **5. Were incomplete outcome data adequately addressed?** | Low risk. | Unclear. | Low risk. | Low risk. | Low risk. | Low risk. | Low risk. | Low risk. | Low risk. | Low risk. | Low risk. | Low risk |
| **6. Was knowledge of the allocated interventions adequately prevented during the study?** | High risk. | Low risk. | Low risk. | Unclear. | Low risk. | Unclear. | Low risk. | Low risk. | Low risk. | Low risk. | Low risk. | Low risk. |
| **7. Was the study adequately protected against contamination?** | Low risk. | Low risk. | Low risk. | Low risk. | Low risk. | Low risk. | Low risk. | Low risk. | Low risk. | Low risk. | Low risk. | Low risk. |
| **8. Was the study free from selective outcome reporting?** | Low risk. | Unclear. | Low risk. | Low risk. | Low risk. | Low risk. | Low risk. | Low risk. | Low risk. | Low risk. | Low risk. | Low risk. |
| **9. Was the study free from other risks of bias?** | Low risk. | High risk. | Low risk. | Low risk. | Low risk. | Low risk. | Low risk. | Low risk. | Low risk. | Low risk. | Low risk. | Low risk. |
| **^1^**No baseline measure.  **^2^**Varied ages and IDP status. | | | | | | | | | | | | |

## Appendix D.2 Grade quality asessment

Table D2 is included as a separate, supplementary file.

## Appendix D.3 Sample risk of bias assessment

| **Appendix D.3.1** Sample risk of bias assessment (primary coder) | |
| --- | --- |
| **Riles, Funk & David (2018)** | |
| **Assessment: Unclear risk of sampling bias, otherwise, low risk of bias** | |
| **Coder: SARAH CARTHY** | |
| Study design. | **Randomised control trial.** *Participants were randomly assigned to the prosocial or control condition.* |
| **1. Was the allocation sequence adequately generated?**  Score “Yes” if a random component in the sequence generation process is described (e.g. Referring to a random number table). Score “No” when a non-random method is used (e.g. performed by date of admission). CCTs and CBAs should be scored “No”. Score “unclear” if not specified in the paper. | **Yes.**  *“After consent was obtained, Qualtrics randomly sorted each participant into a video condition” (p. 9)* |
| **2. Was the allocation adequately concealed?**  Score “Yes” if the unit of allocation was by institution, team or professional and allocation was performed on all units at the start of the study; or if the unit of allocation was by patient or episode of care and there was some form of centralised randomisation scheme, an on-site computer system or sealed opaque envelopes were used. CBAs should be scored “No”. Score “unclear” if not specified in the paper. | **Yes.**  *The randomisation was concealed as Qualtrics randomly sorted each participant into a video condition (p. 9).* |
| **3. Were baseline outcome measurements similar?***  Score “Yes” if performance or patient outcomes were measured prior to the intervention, and no important differences were present across study groups. In RCTs, score “Yes” if imbalanced but appropriate adjusted analysis was performed (e.g. Analysis of covariance). Score “No” if important differences were present and not adjusted for in analysis.** If RCTs have no baseline measure of outcome, score “Unclear”.**  ** If some primary outcomes were imbalanced at baseline, assessed blindly or affected by missing data and others were not, each primary outcome can be scored separately.*  ***If “UNCLEAR” or “No”, but there is sufficient data in the paper to do an adjusted analysis (e.g. Baseline adjustment analysis or Intention to treat analysis) the criteria should be re scored to “Yes”.* | **Unclear.**  *This is an RCT with no baseline measure of outcomes.* |
| **4. Were baseline characteristics similar?**  Score “Yes” if baseline characteristics of the study and control providers are reported and similar. Score “Unclear” if it is not clear in the paper (e.g. characteristics are mentioned in text but no data were presented). Score “No” if there is no report of characteristics in text or tables or if there are differences between control and intervention providers. Note that in some cases imbalance in patient characteristics may be due to recruitment bias whereby the provider was responsible for recruiting patients into the trial. | **Yes.**  *See email below:*  Riles, Julius [rilesj@missouri.edu](mailto:rilesj@missouri.edu)  Fri 16/08/2019 19:33  Yes, the test for equality of variances was satisfactory.  There was no deception, per se, except that participants were told that they would be evaluating media messages and providing social perceptions about a randomly assigned social group (which was Muslims).  Best, Julius |
| **5. Were incomplete outcome data adequately addressed?***  Score “Yes” if missing outcome measures were unlikely to bias the results (e.g. the proportion of missing data was similar in the intervention and control groups or the proportion of missing data was less than the effect size i.e. unlikely to overturn the study result). Score “No” if missing outcome data was likely to bias the results. Score “Unclear” if not specified in the paper (Do not assume 100% follow up unless stated explicitly).  ** If some primary outcomes were imbalanced at baseline, assessed blindly or affected by missing data and others were not, each primary outcome can be scored separately.* | **Yes.**  *No mention of incomplete data.* |
| **6. Was knowledge of the allocated interventions adequately prevented during the study?***  Score “Yes” if the authors state explicitly that the primary outcome variables were assessed blindly, or the outcomes are objective, e.g. length of hospital stay. Primary outcomes are those variables that correspond to the primary hypothesis or question as defined by the authors. Score “No” if the outcomes were not assessed blindly. Score “unclear” if not specified in the paper. | **Yes.**  *Participants completed validated measures, with good internal reliability and the researchers were not involved.* |
| **7. Was the study adequately protected against contamination?**  Score “Yes” if allocation was by community, institution or practice and it is unlikely that the control group received the intervention. Score “No” if it is likely that the control group received the intervention (e.g. if patients rather than professionals were randomised). Score “unclear” if professionals were allocated within a clinic or practice and it is possible that communication between intervention and control professionals could have occurred (e.g. physicians within practices were allocated to intervention or control). | **Yes.** |
| **8. Was the study free from selective outcome reporting?**  Score “Yes” if there is no evidence that outcomes were selectively reported (e.g. all relevant outcomes in the methods section are reported in the results section). Score “No” if some important outcomes are subsequently omitted from the results. Score “unclear” if not specified in the paper. | **Yes.**  *All outcomes were reported.* |
| **9. Was the study free from other risks of bias?**  Score “Yes” if there is no evidence of other risk of biases e.g. selection bias, performance bias, detection bias, attrition bias, reporting bias etc. | **Yes.** |

| **Appendix D.3.2** Sample risk of bias assessment (second coder) | |
| --- | --- |
| **Riles, Funk & David (2018)** | |
| **Assessment: 70% (low risk of bias)** | |
| **Coder: Katie Cox** | |
| Study design. | **RCT**  *Participants were randomly assigned to the prosocial or control condition* |
| **Was the allocation sequence adequately generated?**  Score “Yes” if a random component in the sequence generation process is described (e.g. Referring to a random number table). Score “No” when a non-random method is used (e.g. performed by date of admission). CCTs and CBAs should be scored “No”. Score “unclear” if not specified in the paper. | **Yes.**  *“After consent was obtained, Qualtrics randomly sorted each participant into a video condition”.* |
| **Was the allocation adequately concealed?**  Score “Yes” if the unit of allocation was by institution, team or professional and allocation was performed on all units at the start of the study; or if the unit of allocation was by patient or episode of care and there was some form of centralised randomisation scheme, an on-site computer system or sealed opaque envelopes were used. CBAs should be scored “No”. Score “unclear” if not specified in the paper. | **Yes.**  *Qualtrics randomly sorted each participant into a video condition.* |
| **Were baseline outcome measurements similar?***  Score “Yes” if performance or patient outcomes were measured prior to the intervention, and no important differences were present across study groups. In RCTs, score “Yes” if imbalanced but appropriate adjusted analysis was performed (e.g. Analysis of covariance). Score “No” if important differences were present and not adjusted for in analysis.** If RCTs have no baseline measure of outcome, score “Unclear”.**  ** If some primary outcomes were imbalanced at baseline, assessed blindly or affected by missing data and others were not, each primary outcome can be scored separately.*  ***If “UNCLEAR” or “No”, but there is sufficient data in the paper to do an adjusted analysis (e.g. Baseline adjustment analysis or Intention to treat analysis) the criteria should be re scored to “Yes”.* | **Unclear**  *No baseline outcomes were reported* |
| **Were baseline characteristics similar?**  Score “Yes” if baseline characteristics of the study and control providers are reported and similar. Score “Unclear” if it is not clear in the paper (e.g. characteristics are mentioned in text but no data were presented). Score “No” if there is no report of characteristics in text or tables or if there are differences between control and intervention providers. Note that in some cases imbalance in patient characteristics may be due to recruitment bias whereby the provider was responsible for recruiting patients into the trial. | **Unclear.**  *Not specified.* |
| **Were incomplete outcome data adequately addressed?***  Score “Yes” if missing outcome measures were unlikely to bias the results (e.g. the proportion of missing data was similar in the intervention and control groups or the proportion of missing data was less than the effect size i.e. unlikely to overturn the study result). Score “No” if missing outcome data was likely to bias the results. Score “Unclear” if not specified in the paper (Do not assume 100% follow up unless stated explicitly).  ** If some primary outcomes were imbalanced at baseline, assessed blindly or affected by missing data and others were not, each primary outcome can be scored separately.* | **Yes.**  *No reported missing data* |
| **Was knowledge of the allocated interventions adequately prevented during the study?***  Score “Yes” if the authors state explicitly that the primary outcome variables were assessed blindly, or the outcomes are objective, e.g. length of hospital stay. Primary outcomes are those variables that correspond to the primary hypothesis or question as defined by the authors. Score “No” if the outcomes were not assessed blindly. Score “unclear” if not specified in the paper. | **Unclear.**  *Not specified in the paper.* |
| **Was the study adequately protected against contamination?**  Score “Yes” if allocation was by community, institution or practice and it is unlikely that the control group received the intervention. Score “No” if it is likely that the control group received the intervention (e.g. if patients rather than professionals were randomised). Score “unclear” if professionals were allocated within a clinic or practice and it is possible that communication between intervention and control professionals could have occurred (e.g. physicians within practices were allocated to intervention or control). | **Yes.** |
| **Was the study free from selective outcome reporting?**  Score “Yes” if there is no evidence that outcomes were selectively reported (e.g. all relevant outcomes in the methods section are reported in the results section). Score “No” if some important outcomes are subsequently omitted from the results. Score “unclear” if not specified in the paper. | **Yes.** |
| **Was the study free from other risks of bias?**  Score “Yes” if there is no evidence of other risk of biases e.g. selection bias, performance bias, detection bias, attrition bias, reporting bias etc. | **Yes** |
