# Supplementary material for: Counter‐narratives for the prevention of violent radicalisation: A systematic review of targeted interventions
Source: Campbell Syst Rev. 2020 Aug 12;16(3):e1106. doi: 10.1002/cl2.1106 (PMC8356325; doi:10.1002/cl2.1106)
Supplement: Supplementary file 2 — Supporting information [file CL2-16-e1106-s002.pdf]

Table A1. *Data Extraction Table.*

Study	Sample	Dominant-narrative (DN)	Method of determining the DN	Counter-narrative (CN)	Technique(s) used	Study design	Outcome(s)	Descriptive Statistics	Tests	Effective?	Quality (GRADE)
Cernat (2001)	*N* = 35. Eighty-eight Romanian students (M_{age} = *not provided*, *SD* = *not provided*, 33% female) were recruited from Electromures Highschool of Târgu-Mures in Romania. It is not clear if participants received an incentive for participating.<br>Four participants were excluded from the study due to miscomprehension of the instructions, leaving eighty-four participants in total.<br>Only data pertaining to those in the positive-Hungarian (counter-narrative, *N* = 19) and control conditions (dominant narrative, *N* = 16) are included in this review (*N* = 35).	The dominant narrative amongst the Romanian high school sample was determined to be **anti-Hungarian**. A content analysis of newspapers (Bodó, Cosmeanu, Mátéffy, & Mărginean, 1995) as well as previous research on Romanian attitudes (Mungiu-Pippidi, 1999) revealed "biased presentation [of] historical information" in Romanian society. Furthermore, within the control group, participants' ratings on 1. Stereotype dimensions and; 2. Positive attitudes towards Hungarians were consistently lower than towards Romanians, providing evidence of a dominant, Anti-Hungarian rhetoric amongst the sample.	**- Previous research** (content analysis).<br><br>**- Control group scores.**	Participants completed a **history test** about events in their nation's history; certain sentences were modified to depict Hungarians (amongst other ethnic groups) in more positive ways ('positive Hungarian' condition).	The theoretical framework was not explicitly stated. However, it was guided by previous research suggesting that stereotypes and attitudes may be associated with historical beliefs (Dasgupta & Greenwald, 2001). They seem to employ the use of counter-stereotypical exemplars.	**RCT.** Participants were randomly assigned to one of five conditions: a) positive Hungarian (counter-narrative); b) positive non-Hungarian; c) negative Hungarian; d) negative non-Hungarian and; e) geography test (control). The negative Hungarian condition was determined to be an exacerbated dominant narrative. The control condition was, therefore, selected as the control and results are only reported for the 'Hungarian Positive' (CN) and 'control' (No CN) conditions. Following exposure, participants completed the outcome measures.	**1. Stereotypes dimensions.**<br>*Un-validated.*<br>Using the "percentage of shared attribute method" (range unknown), participants indicated the target groups' level of sociability, efficiency, aggression, extremism. No reliability analysis was provided. Higher numbers indicated higher association with the target group and the stereotype.<br>**2. Evaluations (Hungarians)**<br>*Un-validated.*<br>Participants provided evaluations of the out-group (only data for Hungarians is included in this review) for the following traits on a 7-point Likert scale: admiration, anger, pleasure, respect, rejection, appreciation, attraction, hate, joy, disgust, despise, and annoyance. This measure was un-validated, and no reliability analysis was provided. Higher scores indicated more positive attitudes for the positive evaluations subscale, and vice versa for the negative evaluations subscale (p. 15).<br><br>*Other outcomes not central to the review research question.*<br>**Rosenberg's Self-Esteem scale** (Robinson, Shaver, & Wrightsman, 1991, pp. 127-131).<br>**Sociability (Hungarians)** (stereotype dimensions subscale)<br>**Efficiency (Hungarians)** (stereotype dimensions subscale)<br>**Stereotype dimensions (Romanians)**<br>**Evaluations (Romanians)**<br>**Positive evaluations (Hungarians)**<br>**Affectometer 2.**	**1. Stereotype dimensions**<br>**Aggression**<br>*Geography Test (No CN)*<br>*M* = 246.88<br>*SD* = 98.20<br>N = 16<br>*Positive Hungarian (CN)*<br>*M* = 220.58<br>*SD* = 104.75<br>N = 19<br><br>**Extremism**<br>*Geography Test (No CN)*<br>*M* = 160.25<br>*SD* = 68.16<br>N = 16<br>*Positive Hungarian (CN)*<br>*M* = 157.89<br>*SD* = 69.71<br>N = 19<br><br>**2.Evaluations (Hungarians)**<br>**Negative evaluations**<br>*Geography Test (No CN)*<br>*M* = 25.56<br>*SD* = 10.44<br>N = 16<br>*Positive Hungarian (CN)*<br>*M* = 23.37<br>*SD* = 7.86<br>N = 19	*The analysis below was conducted by review authors*[6]<br><br>**1.** There were no significant differences in aggression $t(33) = 0.76$, $p < .45$, or extremism, $t(33) = 0.10$, $p < .92$, between the 'Hungarian Positive' and control conditions.<br><br>**2.** There were no significant differences between the 'Hungarian Positive' and control conditions in either positive evaluations, $t(33) = -0.74$, $p < .47$ or negative evaluations of Hungarians $t(33) = 0.71$, $p < .48$.<br><br>When all the outcomes were pooled, the mean reduction was SMD = -0.17 (95% CI -0.84 to -0.50), p = 0.62.	**No.**<br>Romanian participants with an anti-Hungarian narrative who were exposed to positive depictions of Hungarians did not report significantly lower stereotyping, negative evaluations or higher overall favourability compared to a control group.	**Low**
Kendrick & Fullerton (2004)	*N* = 105. International students (M_{age} = 22, *SD* = *not provided*, 53.8% female) were recruited from Regent's College in London, England. Participants received £5 for participating. The sample was ethnically and religiously diverse.	In the context of the United States treatment of foreigners, the dominant narrative amongst the sample of international students was determined to be that **Muslims are not free to live and practice their faith in the United States**. In the wake of 9/11, a 2002 poll conducted in 35 countries (cited by Charlotte Beers) provided evidence, internationally, of a dominant set of beliefs in regard to the United States. At the crux of these beliefs, a rhetoric espoused that the United States does not share the same values as predominantly Muslims countries. These values included modesty, obedience, duty, perseverance, freedom, faith, family and learning. This marked difference was said to hinder Muslims from freely living, and practicing their faith, in the United States.	**- Previous research** (poll).	Participants watched "slice-of-life" (i.e. testimonial-style) **television commercials** depicting the positive treatment of "happy and prosperous" Muslim-Americans in the United States. The advertisements aimed to show the 'shared values' of faith, family and education among both Americans and Muslims.	The theoretical framework was not explicitly stated.	**Single group pre/post-test.** Participants completed baseline outcome measures before watching the television commercials and completing the measures again.	**1.** Participants indicated on a 4-point Likert scale their attitudes towards:<br>- The US government.<br>- The US people.<br>- How Muslims are treated in the US<br>This measure was un-validated, and no reliability analysis was provided. Higher numbers indicated more unfavourable attitudes towards the US government, US people etc. (p. 304)<br><br>*Non-quantitative measure(s).*<br>**Advertising copy test** (a qualitative measure which gauges first impressions of the exposure, main messages etc.).	**US government**<br>*Pre (No CN)*<br>*M* = 1.86<br>*SD* = -2.12975<br>N = 104<br>*Post (CN)*<br>*M* = 2.05<br>*SD* = -2.12975<br>N = 104<br><br>**US people.**<br>*Descriptive statistics not available*[4]<br><br>**Treatment**<br>*Pre (No CN)*<br>*M* = 2.82<br>*SD* = -6.137109781<br>N = 104<br>*Post (CN)*<br>*M* = 3.14<br>*SD* = -6.137109781<br>N = 104	**1.** Attitudes towards the US government were significantly more positive after the videos were seen ($p = .013$, $t = 2.54$).<br>**2.** The difference between pre- and post-attitudes towards the US people was not statistically significant.<br>**3.** There was a significant difference between pre-and post-exposure attitudes towards treatment of Muslims ($p < .001$, $t = 3.76$).	**Yes and no.**<br>The Shared Values Initiative was effective at improving participants' attitudes towards the US government and its perceived treatment of Muslims. However, it did not significantly improve attitudes towards the US people.	**Very low**
Ramasubramanian & Oliver (2007)	*N* = approx. 98. One hundred and ninety-six Caucasian-American students (M_{age} = *not provided*, *SD* = *not provided*, "males and females had almost equal representation") were recruited from communications courses in the United States. .<br>It is not clear if participants received an incentive for participating. In this review, only data pertaining to participants in conditions 1, 2, 5 and 6 are reported (i.e. approximately half the sample; see 'Study design' section).	The dominant narrative amongst the Caucasian-American, university sample was determined to be that **African-Americans are hostile, criminal, lazy, drug users, and aggressive.** These attributes (amongst others) were identified with fifty participants through a pilot, free association task. In a second pilot, forty-seven participants indicated the extent to which they agreed with these attributes. The results revealed that attributes used for African-Americans and Asian-Indians that were significantly different from those for Caucasian-Americans were stereotypical. Furthermore, post-test, a one-way repeated measures ANOVA indicated that all participants in the study (irrespective of condition) reported higher levels of hostility towards African-Americans (*M*= 2.29, *SE* = 0.09), compared to Caucasian-Americans (*M*= 1.98, *SE* = 0.08) or Asian-Indians (*M*= 1.94, *SE* = 0.08), Wilks'Λ = 0.90, $F(2, 193) = 10.90$, $p < 0.001$, η2 = 0.10.2, providing evidence of an existing, anti-African-American dominant narrative amongst the sample.	**- Pilot**<br>**- Post-test scores** (all participants)	Participants read a version of a **newspaper article** in which, African-Americans were depicted as "gentle" and associated with entrepreneurial success ('counter-stereotypical' condition(s)).	Through introducing additional information in the form of **counter-stereotypical exemplars**, participants would not have to rely on stereotypical information before making their judgements, thus, reducing their hostile prejudice towards African-Americans	**RCT.** This study was a 2 (+/- media literacy video) x 2 (news story; stereotypical/non-stereotypical) x 2 (race depicted; African-American or Asian-Indian) x 3 (target race; African-American, Asian-Indian or Caucasian-American) factorial design, creating 8 conditions. In conditions 1 & 2, participants watched stereotypical news stories. Participants assigned to these conditions were considered control(s) as they were exposed to	**1. Hostile feelings**<br>Participants provided affective reactions of hostility (e.g. fear, anger, discomfort) or benevolence (e.g. pity, sadness, guilt) towards the target group on a 7-point Likert scale (Eckes, 2002; S. Fiske, Cuddy, Glick, & Xu, 2002; S. T. Fiske, Xu, Cuddy, & Glick, 1999). Cronbach's α = .90 (hostility); α = .82 (benevolence). Higher numbers indicate higher hostility and benevolence, respectively (p. 636).<br><br>**2. 'Feeling thermometer'.** Participants rated their overall feelings of favourability toward African-Americans, on a nine-point thermometer scale ranging from 0° (unfavourable) to 100° (very favourable). The scale was not validated but replicated that of Hugenberg and Bodenhausen (2003)<br><br>*Other outcomes not central to the review research question.*<br>**Benevolent feelings.**	**1. Hostile feelings**<br>*Descriptive statistics not available*[4]<br><br>**2. 'Feeling thermometer'**<br>*Stereotypical (No CN)*<br>*M* = 2.06<br>*SD* = 0.13<br>N = 40<br>*Counter-stereotypical (CN)*<br>*M* = 2.08<br>*SD* = 0.13<br>N = 38	*T*-tests revealed no significant difference(s) in feelings of benevolence/hostility or overall favourability towards African-Americans across stereotypical and counter-stereotypical conditions.	**No.**	**Moderate**

							material which had been provided in pilots testing (see 'Dominant narrative' section). This review, therefore, analysed results from conditions 1, 2, 5 and 6 (stereotypical/counter-stereotypical African-American)					
Gonsalkorale, Allen, Sherman & Klauer (2010)	*Study 2*	*N* = 49. Undergraduate students (M_{age} = *not provided*, *SD* = *not provided*, 78% female) were recruited from a University in the United States. Participants received course credit in exchange for their participation. Approximately 78% of participants self-identified as Caucasian.	The dominant narrative amongst the Caucasian, American University sample was determined to be **racially biased towards white people**. In Study 1, when testing both black and white participants in the IAT, white participants showed the typical pro-White bias, (*M* = 0.40, *SD* = 0.38) and had significantly higher IAT scores than Black participants (*M* = –0.02, *SD* = 0.43), t(18,686) = 47.79, *p* < .001.	**- Study 1 scores** (comparable sample)	Participants completed a **manipulated version of the IAT** ('positive<br>Black and negative White exemplars' condition) which contained images of five popular Black men (e.g., Martin Luther King, Michael Jordan) and five disliked White men (e.g., Adolph Hitler, Charles Manson). Each face appeared with the individual's name and a brief biographical description (e.g., "Martin Luther King: Leader of the Black Civil Rights Movement in the 1960s").	Through the use of **positive exemplars**, novel connections (i.e. associating pleasantness with Black people) would be created and implicit bias (i.e. "black unpleasant") would be reduced	**RCT.**<br>Participants were randomly assigned to the experimental (manipulated IAT) or control (typical IAT; i.e. faces of unknown Black and White individuals) condition.	**1. Implicit Association Task**<br>In this task participants use two computer keys to categorize 12 target images (six Black faces, six White faces) and 16 evaluative words (8 pleasant, 8 unpleasant). The task becomes increasing more complex, with various trials, with responses determining levels of implicit bias towards a target group (e.g. anti-Black, anti-woman etc.) (Greenwald, Nosek, & Banaji, 2003). Reliability could not be determined. Higher IAT scores indicate stronger implicit pro-White preference (p. 161)	**1. Implicit Association Task**<br>*Typical IAT (no CN)*<br>*M = 0.70*<br>*SD =0.34*<br>*N =27*<br>*Manipulated IAT (CN)*<br>*M = 0.36*<br>*SD = 0.41*<br>*N =22*<br>*d* = 0.91	*T*-tests revealed a significant difference in racial bias between participants who completed the manipulated, compared to the typical, IAT, *t*(47) = 3.16, *p* < .003, SMD = -0.90 (95% CI -1.49 to -0.30)	**Yes.**<br>Participants with a racially biased dominant narrative, when exposed to positive exemplars of the target group, showed significantly less racial bias compared to a control group.	**High**
Alhabash & Wise (2012)		*N* = 68. Sixty-eight undergraduate students M_{age}= 20, *SD* = *not provided*, 74% female) were recruited from an introductory Undergraduate advertising course at a large Midwestern university. They received course credit in exchange for their participation.<br>On the implicit attitude measures (AMP) some *(unclear how many)* scores were discarded due to computer malfunction. Additional participants' responses *(unclear how many)* were evaluated as outliers and were discarded, thus resulting in a sample size of 63 for those analyses[3].	In the context of the Israeli-Palestinian conflict, the dominant narrative amongst the American, undergraduate student sample was determined to be **anti-Palestinian / pro- Israeli**. Pre-test attitude measures indicated a significant difference between attitudes towards Palestinians (*M* = 3.67, *SD* = 0.59) compared to Israelis (*M* = 4.15, *SD* = 0.78).	**- Pre-test scores** (compared to attitudes towards another ethnic group)	Participants played the **video game** 'PeaceMaker' (a game which simulates the Israeli–Palestinian conflict with the ultimate tactical goal of achieving peace) from the perspective of the Palestinian president ('Palestinian president' condition).	Through playing the game from the Palestinian perspective, the role play would initiate **self-persuasion**.	**Single group pre/post-test.** The study was a 2 (nationality assignment) x 2 (pre- and post-test attitudes) mixed factorial design. Participants were randomly assigned to play the 'PeaceMaker' video game from the perspective of the Palestinian President (CN) or the Israeli Prime Minister. The latter condition was determined to be an exacerbated dominant narrative and, therefore, in this review, comparisons are made between participants baseline outcome scores and scores after playing 'Peacemaker' in the 'Palestinian president' condition.	**1. National attitudes (towards Palestinians)**<br>*Un-validated.*<br>On a 7-point Likert scale, participants rated 7 statements about each national group regarding (1) favourability; (2) sympathy; (3) belief about the national group's intention for peace; (4) intentionally targeting civilians from the other side; (5) being democratic; (6) being responsible for the violence and, (7) having the right to sole control over the city of Jerusalem (α = .71). Higher numbers indicated more positive attitudes towards Palestinians (p. 366).<br><br>**2. Affective Misattribution Procedure (AMP)**<br>Participants were exposed to portrait pictures of Israelis (12 pictures) and Palestinians (12 pictures) during the Affective Misattribution Procedure task (AMPs) and rated them on valence and arousal (Payne, Cheng, Govorun, & Stewart, 2005). Reliability could not be determined. Higher numbers indicated more positive ratings of the outgroup (p. 366-367).	**1. National attitudes (towards Palestinians)**<br>*Pre-test (no CN)*<br>*M = 3.77*<br>*SD = 0.47*<br>*N = 35*<br>*Post-test (CN)*<br>*M = 4.03*<br>*SD =0.49*<br>*N = 35*<br><br>**2. Affective Misattribution Procedure (AMP)**<br>*Pre-test (no CN)*<br>*M = 0.65*<br>*SD = 0.22*<br>*N = 31*<br>*Post-test (CN)*<br>*M = 0.65*<br>*SD =0.21*<br>*N = 31*	**1.** After playing the role of the Palestinian president, there was a significant increase in participants' favourability toward Palestinians, *t*(34) = -2.47, *p* < .05) between the pre-test and the post-test[8]. SMD = -0.54 (95% CI 0.06 to 1.01).<br><br>**2.** Participants' (implicit) positive ratings toward Palestinians after playing the role of the Palestinian president were the same as their pre-test scores.<br><br>When all the outcomes were pooled, the mean reduction was not significant, SMD = -0.37 (95% CI -0.87 to 0.14).	**Yes and no.**<br>For participants with an anti-Palestinian dominant narrative, playing the role of the Palestinian president in 'PeaceMaker' resulted in higher favourability towards Palestinians. However, there was no change in implicit bias.	**High**
Bilewicz & Jaworska (2013)		*N* = 122. Two hundred and fifty-nine Israeli and Polish high school students were recruited for the study. It is not clear how participants were recruited, or whether they received an incentive for participating. In this review, only data pertaining to Israeli participants (see 'dominant narrative' section) are reported (N = 122, M_{age} = *16.84*, *SD* = *0.49*, 69.1% female).	In the context of deeds perpetrated against their ancestors' during the Holocaust, the dominant narrative amongst the Israeli, high school sample was determined to be **anti-Polish.** Pre-test measures revealed that, before the intervention, Polish participants expressed higher perceived similarity towards out-group members than Israelis, *t*(152) = 4.02, *p* < .001, Cohen's *d* = .65. Poles reported feeling more empowered in relations with Israelis, *t*(151) = 2.57, *p* = .011, Cohen's d = .42, and more accepted in relations with Israelis, t(148) = 3.02, p = .003, Cohen's d = .50, than the Israelis felt in their relations with Poles. Finally, Poles also expressed significantly more positive attitudes toward Israelis than did Israelis toward Poles, *t*(142) = 4.56, *p* < .001, Cohen's *d* = .76.	**- Pre-test scores** (compared to Polish students' responses)	Participants **read short descriptions of 'Heroic Helpers'** (i.e. amongst others, Polish people who had rescued Jews during WWII). Later, the facilitators introduced a specific Polish heroic helper to the group. Students were invited to ask the heroic helper questions and discuss with her the history of the Holocaust.<br><br>*Other intervention components:*<br>**Contact.** After reading the stories, participants also worked in small and mixed Polish−Israeli groups of ten. They discussed common characteristics of rescuers during the Holocaust.	Through the use of **counter-stereotypical exemplars** participants would generally feel more positive attitudes towards what was previously an out-group.	**RCT** *(with wait list control).*<br>Participants were randomly assigned to complete outcome measures before (no CN/control) or after (CN) completing the workshop.	**1. Perceived similarity to the self.**<br>*Un-validated.*<br>Participants indicated their perceived similarity to the out-group(s) (only Polish are used in this review; see dominant narrative section) using 3-items on a 7-point Likert scale (α = .81). Higher numbers indicated higher perceived similarity (p. 169)<br><br>**2. Feeling Thermometer.**<br>Participants indicated their feelings towards the outgroup on a graphical thermometer with a scale ranging from 0° (very negative) to 100° (very positive) (Alwin, 1997) No reliability analysis were provided.<br><br>*Other outcomes not central to the review research question.*<br>**Needs in reconciliation.**<br>*Un-validated.*<br>Participants indicated on 7-point scales to what extent they felt empowered and accepted in their relations with the out-group (i.e. Poles) on two items.	**1. Perceived similarity to [Polish people]**[9]<br>*Wait-list control (no CN)*<br>*M = 4.48*<br>*SD =1.24*<br>*N =78*<br>*Posttreatment (CN)*<br>*M = 5.08*<br>*SD = 1.05*<br>*N = 44*<br><br>**2. Feeling Thermometer**[9]<br>*Wait-list control (no CN)*<br>*M = 70.87*<br>*SD =21.15*<br>*N =78*<br>*Posttreatment (CN)*<br>*M = 82.41*<br>*SD = 16.09*<br>*N = 44*	**1.** Israeli participants from the posttreatment group perceived Polish people as more similar to themselves, *t*(119) = 2.57, *p* = .01, SMD = -0.51 (95% CI = -0.88 to -0.13).<br><br>**2.** Participants in the posttreatment group had more positive attitudes toward Polish people on the 'feeling thermometer', *t*(110) = 3.33, *p* = .001, SMD = -0.59 (95% CI = -0.97 to -0.21) compared to participants in the wait-list control condition.<br><br>When all the outcomes were pooled, the mean reduction was negative and significant, SMD = -0.58 (95% CI -0.96 to -0.21).	**Yes.**<br>The use of 'heroic helpers' in story format, (alongside contact) resulted in more positive feelings towards the perpetrator group (i.e. Polish people) amongst the victim group (i.e. Israelis). The intervention also increased the latter's perceived similarity to their perpetrator group.	**Moderate**

Garagozov (2013)		N = 264. Local Azerbaijanis were recruited from Baku, the capital city of Azerbaijan. It is not clear how participants were recruited, or whether they received an incentive for participating. The sample included internally displaced (M_{age} = 35.2, SD = 13.01, *gender breakdown unclear*, N = 141) and non-internally displaced (M_{age} = 34.3, SD = 13.1, *gender breakdown unclear*, N = 123) participants (IDP / non-IDP). No distinction between the groups is made in this review.	In the context of the Armeno-Azerbaijani Nagorno-Karabakh conflict, the dominant narrative amongst the Azerbaijani sample was determined to be **anti-Armenian.** Supporting the first hypothesis, when measuring all participants implicit attitudes (i.e. errors associating the Armenian President's picture with positive words), there were more errors in reaction to positive words than in reaction to negative words, indicating negative implicit attitudes towards President Sargsyan (M = -.25, SD – 1.10) prior to narrative exposure (i.e. the "priming measure", p. 104). Furthermore, the authors cite nationwide opinion polls which imply hostile relations between Azerbaijan and Armenia. Amongst other findings, the study cites a study which found that 91% of Azerbaijanis saw Armenia as the biggest threat to their country (Caucasus Barometer, 2012). These provide further evidence of the presence of a threatening, dominant narrative of Armenians amongst Azerbaijanis.	**- Pre-test scores** (on Implicit task)<br>**- Previous research** (opinion polls and studies).	Participants read **one of four different stories about the Karabakh conflict presented alongside images** (i.e. four counter-narrative conditions):<br><br>**1. 'Common suffering'**<br>*"…Armeno-Azerbaijani Nagorno-Karabakh conflict created enormous hardships for both peoples. Tens of thousands of people from both sides lost their lives, or became disabled…"*<br>**2. 'Common cultural traits'**<br>*"…there are a lot of common things between Armenians and Azerbaijanis, including traditions, cuisine, music, family relation…"*<br>**3. 'Blame the Russians'**<br>"*Many people believe that the Armeno-Azerbaijani Nagorno-Karabakh conflict started…because Russia had interests in seeing conflict in the Caucasus"*<br>**4. 'Apology'**<br>*"…since the inception of the conflict, Azerbaijani people have experienced many hardships and injustices. On behalf of my people I apologize for all these…"*	Through the development of a "common narrative", participants would create a shared internally consistent vision of the past, present and future (**progressive narrative transformation**).	**RCT.**<br>Participants were randomly assigned to one of four experimental conditions (see 'counter-narrative' section) or a control (no CN) condition where they were not exposed to anything. Following this, they completed the outcome measures.	**Superlab.**<br>*It is not clear if this measure is validated.*<br>A photo of the (then) current Armenian president (Serzh Sargsyan) was presented for 500 milliseconds at the same time as positive (sympathy, love, friendship, kindness) or negative (corrupt, hostile, terrible, hate) words appeared on the participant's screen. Participants were instructed to ignore the picture, and to simply indicate the valence of the word by pressing one of two keys on the keyboard, marked "good" or "bad" and to respond as quickly as they could within a specified "response window" (500 milliseconds). The extent to which Sargsyan's image elicited more errors on positive compared to negative words, provided evidence of pre-potent, negative associations with Sargsyan. No reliability analysis was provided. Higher numbers indicated more negative attitudes towards President Sargsyan (p. 105).<br><br>*Other outcomes:*<br>Participants in the counter-narrative conditions responded to an additional outcome: 'agreement with narrative type'. Control participants were likely not asked this question and, therefore, this outcome was excluded from the review.	**Superlab.**<br>*Descriptive statistics not available*[4]<br><br>In case they're needed.<br>No CN/control (N = 50)<br>1 "Common suffering" (N = 65), 2 "Common cultural traits" (N = 50), 3 "Blame the Russians" (N = 49) or; 4 "Apology" (N = 50);	A 5 (condition) x 2 (IDP vs non-IDP) ANOVA revealed no significant differences on the Superlab measure. The condition which resulted in less implicit bias was the "Apology" condition. Participants who read the "Blame the Russians" narrative had the most negative implicit attitudes toward President Sargsyan,	**Unclear.**<br>There are no comparisons provided between the four experimental and control conditions, so it is unclear if the counter-narratives were more effective than the control.	**Moderate**
Alhabash & Wise (2015)		N = 172. One hundred and seventy-two students (M_{age} = 20.80, SD = 2.89, 60.5% female) were recruited from introductory (69.8% freshmen or sophomore) courses at a large Midwestern university in the United States. They received course credit in exchange for their participation. The majority of the sample were White/Caucasian (75.6%). The sample for the AMP was 161[3] due to computer malfunction. N = 85 participants completed the explicit stereotypes measure and N = 78 completed the AMP in the Palestinian president condition;	In the context of the Israeli-Palestinian conflict, the dominant narrative amongst the American, undergraduate student sample was determined to be **anti-Palestinian / pro- Israeli.** Supporting the first and second hypotheses, pre-test results indicated that participants expressed significantly more positive stereotypes of Israelis (M = 6.01, SD = 1.18) than Palestinians (M = 5.47, SD = 1.31), $t(171)$ = 4.75, p < .001, d = .43. Finally, on the AMP (implicit measure), participants associated more pleasantness with Israeli faces (M = 64.34%, SD = 18.68%) compared to Palestinian faces (M = 58.76%, SD = 20.80%), $t(160)$ = 3.30, p < .01, d = .28.	**- Pre-test scores** (compared to attitudes towards another ethnic group)	*See Alhabash & Wise (2012).*	*See Alhabash & Wise (2012).*	*See Alhabash & Wise (2012)*	**1. Explicit stereotypes.**<br>*Un-validated.*<br>Participants were asked to rate 14 different semantic differential items for each national group on a 7-point scale. These items include clean/dirty, good/bad, smart/ stupid, beautiful/ugly, industrious/lazy, strong/weak, sociable/unsociable, loyal/treacherous, educated/ignorant, hospitable/inhospitable, brave/coward, trustworthy/untrustworthy, tempered/violent, and merciful/cruel. No reliability analysis provided. Higher numbers indicated more positive stereotypes<br><br>**2. Affective Misattribution Procedure (AMP)**<br>Each trial consisted of a white screen (1000 ms), target picture of a famous Palestinian or Israeli (75 ms), a Chinese pictograph (200 ms), and a noise image. The percentage of pictures evaluated as pleasant was calculated for each national group (Payne et al., 2005). Higher scores indicated higher more positive feelings towards Palestinians. *Reliability could not be determined.*	**1. Explicit stereotypes (towards Palestinians)**<br>*Pre-test (no CN)*<br>*M = 5.41*<br>*SD = 1.28*<br>*N = 85*<br>*Post-test (CN)*<br>*M = 5.28*<br>*SD =1.18*<br>*N =85*<br><br>**2. AMP (percentage of pleasantness with Palestinian faces)**[3]<br>*Pre-test (no CN)*<br>*M = 0.58*<br>*SD = 0.21*<br>*N = 78*<br>*Post-test (CN)*<br>*M = 0.54*<br>*SD =0.23*<br>*N =78*	1. Participants who played the role of the Palestinian President did not observe a significant change in their explicit stereotypes of Palestinians between the pre-test and the post-test $t(168)$ = -0.69, p = 0.49, SMD = 0.18 (95% CI = -0.14 to -0.50).<br><br>2. The same was found[6] for score on the AMP, $t(154)$ = -1.13, p = .26, SMD = 0.18 (95% CI = -0.13 to -0.50) .<br><br>When all the outcomes were pooled, there was no reduction; the effect was positive, SMD = 0.11 (95% CI -0.21 to 0.42).	**No.**<br>For participants with an anti-Palestinian dominant narrative, playing the role of the Palestinian president in 'PeaceMaker' did not result in higher favourability towards Palestinians, or any change in implicit bias.	**High**
Cohen, Tal-Or & Mazor-Tregerman (2015)	*Study 1*	N = 70. One hundred and thirty-two students (M_{age} = 25.24, SD = 5.89, 75% female) were recruited from a large Israeli university. Some (N = 36), but not all, received a financial incentive (30 shekels) for participating. Based on pre-exposure attitudes towards demonstrations (35% were pro-demonstration, 54% were anti-demonstration and 11% held no opinion), those with no opinion (N = 16) were removed from the study analysis. In this review, only data pertaining to participants with baseline, anti-demonstration attitudes are reported in this review. See 'dominant narrative' section.	In the context of the Israeli-Palestinian conflict, the dominant narrative amongst the Israeli, university sample was determined to be **against on-campus demonstrations.** Before exposure, pre-test scores indicated a clear bimodal distribution of attitudes on the above topic (35% were pro-demonstrations, 54% anti, and 11%, who were removed from the study, did not have an opinion). In this way, the study provided evidence of two, dominant narratives in the sample: pro-demonstration and anti-demonstration. Due to its proximity to violent extremism-related outcomes, the latter (anti-demonstration) was chosen as the dominant narrative. See Friedman (1986) and Luz (2002) for descriptions of typical arguments purported by violent extremist groups such as the Jewish Underground Movement and Terror Against Terror; similar narratives are purported in justification of settler violence in the West Bank (Ginges, Atran, Sachdeva, & Medin, 2011); this condition was deemed more proximal to the outcomes of interest in this review.	**- Pre-test scores** (compared to fellow Israeli, university students)	Participants **read a story in which two friends face a dilemma over their countering views on on-campus demonstrations** (approx. 2000 words). Each represented a side, making either pro- or anti-demonstrations statements throughout. The virtuousness of each character was manipulated and the version of the narrative in which the pro-demonstration character is rendered virtuous is selected as the 'counter- narrative' ('identification with pro-demonstration' condition).	Alongside offering **arguments that directly countered those in the dominant narrative** (anti-demonstration), the exposure attempted initiate persuasion by eliciting participants' **identification with the protagonists** (Cohen, 2001) as well as **transportation** into the narrative (Green & Brock, 2000).	**RCT.** Although this study was designed as 2 (identification with pro-demonstration or anti-demonstration character) × 2 (baseline attitudes regarding in-campus demonstrations: pro-demonstrations or anti-demonstrations) experimental design, only data pertaining to anti-demonstration participants were of interest to our review question (see 'dominant narrative' section). Comparisons are made between 'virtuous anti-demonstration' (no CN/control) and 'virtuous pro-demonstration' (CN) conditions[1]. Following assignment to one of these conditions, participants	**Post-exposure attitudes**<br>*Un-validated.*<br>Participants indicated their agreement with six pro-demonstration statements (e.g., "each and every citizen has the right to express his or her opinions everywhere, including on campus") and six anti-demonstration statements that were reverse coded (e.g., "It should be forbidden for Arab students to demonstrate in the heart of the campus, a campus built and funded by the State of Israel, against the state, against Zionism or in support of the violent Palestinian struggle against Israel") on a 7-point Likert scale (α = 0.83). Higher numbers indicated higher pro-demonstration attitudes (p. 9).<br><br>*Other outcomes not central to the review research question.*<br>**The Identification Scale** (Cohen, 2001)<br>**The Transportation Scale** (Green & Brock, 2000)<br>**Character virtue.** *Un-validated.*	**Post-exposure attitudes (towards demonstrations)**[9]<br>Virtuous anti-demonstration (no CN/control)<br>M = 4.05<br>SD =1.48<br>N = 36<br><br>Virtuous pro-demonstration (CN)<br>M = 3.93<br>SD = 1.51<br>N = 34	Participants in the virtuous pro-demonstration condition (CN) reported lower agreement with the pro-demonstration statements then those in the control (virtuous anti-demonstration) condition.<br>When all the outcomes were pooled, the mean reduction was SMD = -0.08 (95% CI -0.55 to -0.39).This difference[6] was not significant, $t(68)$ = -0.34, p = .74.	**No.**<br>For participants with a dominant, anti-demonstration narrative, rendering a pro-demonstration character more virtuous in a two-sided narrative did not increase their agreement with pro-demonstration statements. Conversely, their control counterparts reported more agreement.	**Moderate**

							completed the outcome measures.					
Saleem, Prot, Anderson & Lemieux (2015)	*Experiment 3*	*N* = 194. Four hundred participants (*M*age = *not provided or accessible*, *SD = not provided or accessible*, 49% female) were recruited from two pools: a large, mid-western University in the United States and an (MTurk) sample. For this review, only data pertaining to those in the 'neutral' (no CN) (*N* = 94) and 'counter-stereotypical' (CN) (*N* = 100) video conditions are reported. See 'dominant narrative' section.	In the context of American foreign policy, the dominant narrative amongst the American, student sample was determined to be **anti-Muslim.**<br>A 'no video' condition was included in the study to observe attitudes at baseline, as well as to assess the extent to which the stereotypic, neutral, and counter-stereotypic videos affected baseline attitudes. This condition was the second highest of all the conditions (including the neutral condition), providing evidence of an existing, anti-Muslim narrative in the sample irrespective of experimental condition.	**- Control group scores** (compared to stereotypic and neutral conditions)	Participants watched an 'ABC' **news-clip featuring Muslims volunteering in the local area during Christmas.** It included a brief statement from the local Imam who emphasized the importance of the Muslim community coming together to help their fellow citizens during Christmas.	Through creating associations between Muslims and positive behaviours (i.e. **counter-stereotypical exemplars**), participants would create positive schematic associations of Muslims.	**RCT.** Participants were randomly assigned to a 'stereotypic', 'counter-stereotypic', or 'neutral' condition. The stereotypic condition was determined to be an exacerbated dominant narrative. Therefore, only data pertaining to participants assigned to the counter-stereotypic (CN) and neutral/no CN (a news-clip about a football rescheduling due to Ramadan in a local high school, aired on 'ABC') conditions are included in this review. Following exposure, participants completed outcome measures indicating their attitudes towards Muslims.	*All un-validated.*<br>**1. Perceptions of Muslims as aggressive (VATT)**<br>Participants rated their agreement with 5 statements (e.g. "Muslims are dangerous") on a 5-point Likert scale. (α = .90 for Study 2). Higher numbers indicated higher perceptions of Muslims as aggressive (p. 856).<br><br>**2. Support for civil restrictions for Muslim Americans (MORECIVIL)**<br>Participants rated their agreement with 7 statements (e.g. Muslim Americans should not be allowed to vote) on a 6-point Likert scale. No reliability analysis provided for Study 3 (α = .92 for Study 2). Higher numbers indicated higher support for civil restrictions (p. 856).<br><br>**3. Support for military action in Muslim countries (MILTOT)**<br>Participants rated their agreement with 9 statements (e.g. I would support the use of U.S. military to reduce<br>the influence of Islam on other countries) on a 5-point Likert scale (Henry, Sidanius, Levin, & Pratto, 2005). No reliability analysis provided for Study 3 (α = .86 for Study 1). Higher numbers indicated higher support for military action in Muslim countries (p. 856).	[5]**1. Perceptions of Muslims as aggressive (VATT)**<br>*Neutral video (No CN)*<br>*M* = 2.09<br>*SD* = 0.92<br>*N* = 94<br><br>*Counter-stereotypical (CN)*<br>*M* = 2.07<br>*SD* = 0.91<br>*N* = 100<br><br>**2. Support for civil restrictions for Muslim Americans (MORECIVIL)**<br>*Neutral video (No CN)*<br>*M* = 1.79<br>*SD* = 0.77<br>*N* = 94<br><br>*Counter-stereotypical (CN)*<br>*M* = 1.84<br>*SD* = 0.78<br>*N* = 100<br><br>**3. Support for military action in Muslim countries (MILTOT)**<br>*Neutral video (No CN)*<br>*M* = 2.71<br>*SD* = 0.86<br>N = 94<br><br>*Counter-stereotypical (CN)*<br>*M* = 2.65<br>*SD* = 0.85<br>N = 99	There were no significant differences[6] between the counter-stereotypic (CN) and neutral (no CN) condition on any of the outcome variables.<br><br>When all the outcomes were pooled, the mean reduction was not significant, SMD = -0.02 (95% CI -0.30 to -0.26).	**No.**<br>Participants with a dominant, anti-Muslim narrative who were exposed to counter-stereotypical exemplars did not report fewer negative attitudes towards Muslims compared to a control group.	**Moderate**
Banas & Richards (2017)		*N* = 187. Two hundred and twenty-five students (*M*age = 19.71, *SD* = 1.40, 43% female) were recruited from communications courses in a southern University in the United States. Participants received course credit for participation**.** Approximately 78% of participants self-identified as Caucasian. Participants who held "conspiratorial attitudes about 9/11" (*N* = 38) were excluded from the analysis.	The dominant narrative was experimentally introduced after exposure to the counter-narrative. Participants were exposed to a 40-minute film clip of 'Loose change: Final cut' (Avery, 2007), an **anti-government, conspiracy-theory** film detailing the supposed role of the United States government in 9/11.	**- Experimentally introduced pre-exposure.**	Participants **read a message containing counter-arguments**. The message provided the arguments made in the appeal, as well as refutations to dispel them.<br><br>*Other intervention components:*<br>Participants read a message which alerted them that they were going to be exposed to a "persuasive appeal".	Through the use of counter-arguments (**refutational pre-emption*),** as well as **explicit forewarning*** of the persuasive appeal, the message aimed to trigger counter-arguing and perceived threat.<br><br>*****Both are components of inoculation (Banas & Rains, 2010; Compton & Ivanov, 2012)	**RCT.**<br>Participants were randomly assigned to the experimental (inoculation) or control (irrelevant message about the history of sushi) condition before they completed the outcome measures.	**Attitudes towards the conspiracy.**<br>Participants rated their agreement with the following item "the position advocated in the message was:" on 6 parameters: negative/positive, bad / good, unfavourable / favorable, unacceptable / acceptable, wrong / right, and foolish / wise. Scoring was on a 7-point Likert scale (Burgoon, Cohen, Miller, & Montgomery, 1978) (α = .96). Higher scores indicated higher agreement with the conspiratorial statements ("positive", "good", "favourable", etc.) (p. 171-172).<br><br>*Other outcomes not central to the review research question:*<br>**Traditional threat** (Burgoon et al., 1978)<br>**Motivational threat**<br>**Fear**	**Attitudes towards the conspiracy**<br>*Control/sushi video (no CN)*<br>*M* = 3.89<br>*SD* = 1.30<br>N = 85[5]<br><br>*Inoculation (CN)*<br>*M* = 3.06<br>*SD* = 1.55<br>N = 102[5]<br><br>*d* = 0.58	There was a significant difference[6] in participants' attitudes towards the conspiracy between the control and experimental conditions, $t(270) = 4.29$, $p < .001$. The mean reduction conspiratorial attitudes SMD = -0.57 (95% CI -0.87 to -0.28).	**Yes.**<br>For participants who were exposed to a dominant, conspiratorial narrative, those who received a counter-narrative in the form of refutational pre-emption and counter-arguments reported lower adoption of conspiratorial attitudes.	**Moderate**
Bruneau, Lane & Saleem (2017)	Study 1	*N* = 223. Three hundred and thirty-one participants were recruited from Amazon's Mechanical Turk (MTurk). The ethnicity of the sample was not provided. It is not clear if participants received an incentive for participation. For this review, only data pertaining to participants assigned to the control (*N* = 130 *M*age = 33.7, *SD* = 12.7; 58.5% male) and counter-narrative (*N* = 93, *M*age = 33.6, *SD* = 12.1; 48.4% male) conditions are reported. See 'dominant narrative' section.	In the context of the Israeli-Palestinian conflict, the dominant narrative amongst the (presumably international) sample was observed to be that the **Palestinian resistance is a violent one, justifying violent retaliation by Israel.** Post-experimental scores on the 'Feeling Thermometer' showed no significant difference between participants who viewed the 'Palestinian violent' video ("Omar"; *M* = 50. 62, *SD* = *not provided*) and those who viewed the control video ("Chasing Ice"; *M* = 50. 75, *SD* = *not provided*). Conversely, the latter was actually slightly higher. In other words, participants in the control condition showed similar levels of prejudice towards Palestinians to those in the violence-promoting condition. This provides evidence of the dominant narrative in the sample.	**- Control group scores** (compared to 'violent' and control conditions)	Participants **watched a trailer for the film "Budrus"**; a documentary-style film that follows the non-violent, Palestinian campaign in the Israeli occupation of Palestine.	An **alternative account** of events was offered in an attempt to restore favourability towards the out-group, and challenge an "entrenched" dominant narrative,	**RCT.** Participants were randomly assigned to a counter-narrative, violence promoting or control condition. In this review, only data pertaining to participants assigned to the 'counter-narrative' (CN) and 'control'/no CN (global warming documentary) conditions are reported; the violence promoting condition ("Omar", a film depicting the violent Palestinian resistance) was determined to be an exacerbated	**1. Palestinian violence.**<br>*Adapted.*<br>Participants rated their agreement with 5 statements (e.g. "Palestinians are much more violent than other groups") adapted from Pratto, Sidanius, Stallworth, and Malle (1994) on a 0-100 scale. No reliability analysis provided for Study 1 (α = .87 for Study 2). Higher scores indicated higher agreement with the statements.<br>**2. 'Feeling Thermometer'.** Participants rated their feelings towards Palestinians (and others ethnicities) on a thermometer scale ranging from 0 (unfavourable) to 100 (very favourable) (Haddock, Zanna, & Esses, 1993). Reliability could not be determined. Responses were reverse-scored, so higher scores indicated stronger prejudice (p. 748).<br><br>*Other outcomes not central to the review research question.*<br>**Pro-social behaviour.**<br>*Un-validated.*<br>Participants were given the option to donate up to $1 (the full amount of their "bonus" for completing the survey) to ''the UN Relief and Works Agency (UNRWA) http://www.unrwa.org/donate; an organisation "working to improve conditions for the children in Gaza". *Reliability could not be determined.*	**1. Palestinian violence**<br>*Chasing Ice' (no CN)*<br>*M = 42.08*<br>*SD = 23.59*[10]<br>*N = 130*<br>*'Budrus' (CN)*<br>*M = 30.19*<br>*SD = 23.62*[10]<br>*N = 93*<br><br>**2. 'Feeling Thermometer'**<br>*'Chasing Ice' (no CN)*<br>*M* = 50.75<br>*SD* = 23.76[10]<br>N = 130<br>*'Budrus' (CN)*<br>*M* = 41.46<br>*SD = 23.79*[10]<br>N = 93	1. Significant differences in 'Palestinian violence' were found between the counter-narrative and control conditions: $t(221) = 3.76$, $p < .001$, d = .51. The mean reduction perceptions of Palestinians as violent was SMD = -0.51 (95% CI 0.24 to 0.78), *v* = 0.02.<br><br>2. Significant difference were also found on 'Feeling Thermometer' scores between the counter-narrative and control condition $t(221) = 2.87$, $p = .004$, $d = .39$. The mean reduction in negative feelings was, SMD = -0.39 (95% CI 0.12 to 0.66), *v* = 0.02.<br><br>When all the outcomes were pooled, the mean reduction was significant, SMD = -0.45 (95% CI -0.71 to -0.18).	**Yes.**<br>The counter-narrative was effective in reducing negative perceptions of Palestinians and prejudice. The effect size was	**High**

							dominant narrative.					
	Study 2	N = 417. Six hundred and ninety-two participants were recruited from Amazon's Mechanical Turk (MTurk). It is not clear if participants received an incentive for participation. For this review, only data pertaining to participants assigned to the control (N = 217 M_{age} = 34.7, SD = 11.6; 51.2% male) and counter-narrative (N = 200, M_{age} = 35.2, SD = 11.4; 48.5% male) conditions are reported. See 'dominant narrative' section.	*See Study 1.*<br>Study 2 provided further evidence in support of an existing, anti-Palestinian narrative in the sample. For the bivalent, pre-test evaluations of the Israeli–Palestinian conflict (scaled Palestinian -10 to Israeli +10), Israelis were rated to be personally more favoured than Palestinians, M = 1.82, SD = 5.8; t(551) = 7.4, p < .001. Relative to the other conflict groups, Palestinians were also viewed to be one of the most "inherently violent".	**- Pre-test scores** (compared to attitudes towards Israelis)	*See Study 1.*	*See Study 1.*	*See Study 1.*	**1. Palestinian violence.** *See Study 1* (α = 0.87).<br>**2. 'Feeling Thermometer'.** *See Study 1.*<br><br>*Other outcomes not central to the review research question, or conceptually comparable to other outcome(s) in the study.*<br>**4. Trust toward Palestinians / Israelis.**<br>*Un-validated.*<br>Participants indicated from 0 (completely disagree) to 100 (completely agree) the extent to which they agreed with the following statement(s): "I trust that Palestinians / Israelis are committed to finding a peaceful and lasting solution to the conflict in the Middle East". *No reliability analysis is provided.*<br>**5. Narrative engagement** (Busselle & Bilandzic, 2009). (α = 0.79 - 0.85). Higher numbers indicate higher engagement.	**1. Palestinian violence**<br>*'Chasing Ice' (no CN)*<br>*M = 44.11*<br>*SD = 21.46*[10]<br>N = 217<br>*'Budrus' (CN)*<br>*M = 35.19*<br>*SD = 21.50*[10]<br>N = 200<br><br>**2. 'Feeling Thermometer'**<br>*'Chasing Ice' (no CN)*<br>*M* = 56.62<br>*SD = 26.57*[10]<br>N = 217<br>*'Budrus' (CN)*<br>*M* = 45.42<br>*SD = 26.48*[10]<br>N = 200	**1.** Significant differences between CN and control on Palestinian violence: $t(415) = 4.17$, $p < .001$, $d = .41$. The mean reduction perceptions of Palestinians as violent was SMD = -0.41 (95% CI -0.21 to -0.60), v = 0.01.<br><br>**2.** Significant difference between CN and control on Palestinian prejudice: $t(414) = 4.23$, $p < .001$, $d = .42$.<br><br>When all the outcomes were pooled, the mean reduction was significant, SMD = -0.42 (95% CI -0.61 to -0.22).	**Yes.**<br>The counter-narrative was effective in reducing negative perceptions of Palestinians and prejudice.	**High**
	Study 3	N = 227. Three hundred and thirty-five participants were recruited from Amazon's Mechanical Turk (MTurk). It is not clear if participants received an incentive for participation. For this review, only data pertaining to participants assigned to the control (N = 115 M_{age} = 33.0, SD = 12.4; 49.6% male) and counter-narrative (N = 112, M_{age} = 35.6, SD = 12.4; 41.1% male) conditions are reported. See 'dominant narrative' section.	*See Studies 1 and 2.*<br>Study 3 provided further evidence in support of an existing anti-Palestinian narrative in the sample as measured by the fourth dependent measure. Participants in the control condition (M = 48.68, SD = *not provided*) had a stronger anti-Palestinian ideology compared to the violence-promoting condition (M = 46.10, SD = *not provided*).	**- Control group scores** (compared to the control condition)	*See Study 1.*	*See Study 1.*	*See Study 1.*	**1. 'Feeling Thermometer'.** *See Study 1.*<br>**2. Palestinian violence.** Same as Study 1 plus additional (reverse coded) item: "Palestinians commit violence against Israelis largely because they are provoked" (α = 0.93).<br><br>*Other outcomes not central to the review research question, or conceptually comparable to other outcome(s) in the study.*<br>**1. Perceived Palestinian hatred of Jews**.<br>*Un-validated.*<br>Participants rated the statement "How strongly do you think Palestinians hate Jews?" from 0 (no hatred) to 100 (a lot of hatred).<br>**2. Trust (toward Palestinians)** *See Study 1.*<br>**3. Anti-Palestinian ideology**.<br>*Un-validated.*<br>Participants rated the ''rationality'' of five common anti-Palestinian narratives (adapted from Bruneau & Saxe, 2010) (e.g. "Palestinians could be living next to Israel as a modern country, but instead they have chosen violence and terrorism") on a 0-100 scale. (α = .89).<br>**4. Petition**<br>*Un-validated.*<br>Participants were given the opportunity to sign a petition (using an ID) either in support of or opposition to Palestinian admittance to the ICC. Adding a signature to the supportive petition = 1; adding a signature to the oppositional petition = -1 and no signature = 0. *No reliability analysis was provided.*	**1. 'Feeling Thermometer'**<br>*'Chasing Ice' (no CN)*<br>*M* = 51.68<br>*SD = 26.84*<br>*N* = 115<br>*'Budrus' (CN)*<br>*M* = 45.69<br>*SD = 26.84*<br>*N* = 112<br><br>**2. Palestinian violence**<br>*'Chasing Ice' (no CN)*<br>*M* = 44.13<br>*SD = 22.92*[10]<br>*N* = 115<br>*'Budrus' (CN)*<br>*M* = 35.61<br>*SD = 22.92*[10]<br>*N* = 112	**1.** No significant difference between CN and control on the 'Feeling Thermometer': *no data provided.*<br><br>**2.** Significant difference between CN and control on Palestinian violence: *no data provided.*<br><br>When all the outcomes were pooled, the mean reduction was significant, SMD = -0.29 (95% CI -0.55 to -0.03).	**Yes and no.**<br>***Yes.***<br>The counter-narrative was effective in reducing negative perceptions of Palestinians.<br><br>***However.***<br>In contrast with studies 1 and 2, there was no significant difference between CN and control conditions on the 'Feeling Thermometer'.	**High**
Cehajic-Clancy & Bulewicz (2017)	Study 1	N = 75. Young people (M_{age} = 20.09, SD = 3.74, 61% female) were recruited from eight cities and towns around Bosnia and Herzegovina. It is not clear how participants were recruited, or whether they received any incentive for participation.	The dominant narrative amongst the sample of young Bosniaks, Serbs, & Croats was determined to be that their respective **out-group was immoral**. Cited research conducted in Bosnia and Herzegovina (BIH) has shown low levels of intergroup contact, forgiveness, trust and belief in reconciliation among its citizens (Cehajic, Brown, & Castano, 2008). This sample was taken from cities and towns in BIH and included participants from various sides of the conflict (Bosniaks, Serbs, & Croats). Although there is no experimental evidence from this specific sample, the earlier research cited in the study lends credence to the presence of a dominant narrative of an "immoral" outgroup.	**- Previous research** (cross-sectional).	*Sequential order of both the counter-narrative* **(CN)** *and non-counter-narrative* **(CN)** *components of the intervention which was delivered in the form of an 8-week workshop.*<br><br>1. **(CN) Film trailer.**<br>Participant watched a trailer from an educational series about stories of rescuers before being introduced to the workshop.<br><br>2. **(CN)** *Ice-breakers*<br><br>3. **(CN) Bosnian rescuer case-study**.<br>Participants worked under the guidance of a facilitator to identify terms such as "conflict, victim, perpetrator, active and passive bystander" in the story of a Bosnian rescuer.<br><br>4. **(CN)** *Group-work/contact Divided into mixed groups to discuss their own case studies.*<br><br>5. **(CN) Film.**<br>Individual stories of "heroic helpers" from all sides of the Bosnian War (1992-1995) were shown in the film 'Ordinary Heroes'.	Through exposing participants to **moral exemplars** of the out-group, the counter-narratives attempted to increase their awareness of the "historical, *moral* variability of the out-group".	**Single group pre/post-test.** All participants completed measures of 'belief in reconciliation' and 'forgiveness' before and after the intervention.	**1. Belief in reconciliation**<br>*Un-validated.*<br>Participants responded on a 5-point Likert scale (5 = strongly agree) to the following items:<br>- I doubt that we will ever be able to live together in peace<br>- I believe that we can cooperate together<br>- I believe that we can build a country together<br>Higher scores indicated stronger belief (p. 291). Pre: α = .66; post: α = .72.<br><br>**2. Forgiveness**<br>Adapted from Cehajic et al. (2008), participants responded on a 5-point Likert scale (5 = strongly agree) to the following items:<br>- I am ready to forgive other groups things that they have done during the war.<br>- I could never forgive the committed crimes.<br>- My group should never forgive other groups their misdeeds.<br>Higher scores indicated higher "forgiveness" (p. 291). (Pre: α = .78; post: α = .83).	**1. Belief in reconciliation**<br>*Pre-test (no CN)*<br>*M* = 3.60<br>*SD* = 0.96<br>*N* = 75<br>*Post-test' (CN)*<br>*M* = 3.79<br>*SD* = 0.94<br>*N* =75<br><br>**1. Forgiveness**<br>*Pre-test (no CN)*<br>*M* = 3.85<br>*SD* = 0.96<br>*N* = 75<br>*Post-test' (CN)*<br>*M* = 4.07<br>*SD* = 0.82<br>*N* =75	**1.** There was no difference[6] in reconciliatory beliefs from pre- to post-test, $t(148) = 1.23$, p = .22.<br><br>**2**. Similarly, when comparing the pre- and post-test forgiveness scores, the difference[6] was not significant, $t(148) = 1.51$, p = .13.<br><br>When all the outcomes were pooled, the mean reduction was, SMD = -0.23 (95% CI -0.55 to -0.09).	**No.**<br>Exposing participants to a moral-exemplar-based intervention did not significantly improve their belief in reconciliation, nor forgiveness towards their perceived outgroup.	**Low**

					6. **(CN)** *Discussion. Participants discussed the film, and what they had learned during the workshop.*							
	Study 2	N = 85. Young people (M_{age} = 20, SD = 7.22, *unknown gender division*) were recruited from eight cities and towns around Bosnia and Herzegovina. It is not clear how participants were recruited, or whether they received any incentive for participation.	*See Study 1.*	*See Study 1.*	*See Study 1.* The intervention used in Study 2 was one week longer (i.e. 9 weeks).	*See Study 1.*	*See Study 1.*	1. **Belief in reconciliation**<br>*See Study 1.* (Pre: α = .76; post: α = .73).<br>2. **Intergroup anxiety**<br>- Participants indicated their trust, confidence, discomfort etc. towards the out-group. Adapted from Stephan and Stephan (1985).<br>Higher scores indicated higher anxiety (Lolliot et al., 2015, p. 666). Pre: α = .54; post: α = .61.<br><br>*The other outcomes were not asked specifically about a target group but, rather, out-groups in general:*<br>**Contact intentions**<br>- I would like to have better relationship with other groups<br>- I would like to have more friends from other groups<br>(adapted from Čehajić-Clancy et al., 2008). Higher numbers indicated higher contact intentions. Pre: α = .92; post: α = .84).<br>**Belief in humanity**<br>*Un-validated*<br>- In principal human beings are good<br>- Despite all, the good in people always prevails<br>- There will always be more good than bad in this world<br>Higher scores indicated higher 'belief' (p. 292). Pre: α = .74; post: α = .69.<br>.	**1. Belief in reconciliation**<br>*Pre-test (no CN)*<br>M = 3.73<br>SD = 1.03<br>N = 85<br>*Post-test' (CN)*<br>M = 3.75<br>SD = 0.95<br>N =85<br><br>**2. Intergroup anxiety**<br>*Pre-test (no CN)*<br>M = 2.45<br>SD = 1.13<br>N = 85<br>*Post-test' (CN)*<br>M = 2.17<br>SD = 0.96<br>N =85<br>d = 0.26	*The analysis below was conducted by review authors*[6]<br><br>**1.** There was no difference in reconciliatory beliefs from pre- to post-test, $t(168) = 0.13$, p = .90, SMD = 0.02 (95% CI -0.28 to 0.32).<br><br>**2.** The difference in intergroup anxiety from pre- (M = 2.45, SD = 1.13) to post-test (M = 2.17, SD = .69), was not significant. The mean reduction SMD = -0.27 (95% CI -0.57 to 0.04), p = .08.<br><br>When all the outcomes were pooled, the mean reduction was not significant, SMD = -0.15 (95% CI -0.45 to 0.15).	**No.**<br>The intervention did not create any significant change in participants' belief in reconciliation or intergroup anxiety.	**Low**
Frischlich, Rieger, Morten, & Bente (2018)	Study 1	N = 338. Participants (M_{age} = 23, SD = 4.31, 48% female) were recruited from cities in Western Germany and included both students (67%) and apprentices. Participants received €15 for participation. Most participants self-identified as Christian (50%), Muslim (28 %), or Atheist and 52% had a first- or second-generation migration background.	Two dominant narratives were experimentally introduced.<br><br>Non-Muslim participants were exposed to **two right-wing extremism (RWEX) narratives**. The first shows a young man critiquing "the system", calling on Germans to fulfil their duty against injustice. The second is more dramatic, showing historical images and foreshadowing. Participants indicated their 'extremist attitudes' post-exposure<br><br>Muslim participants were experimentally exposed to **two Islamic extremism (ISEX) narratives**. The first showed an ISEX activist describing the persecution of Muslims around the world, demanding fellow Muslims to fight. The second was more dramatic, showing attacks on Iraq and how the nation of Islam needs an organisation to protect it ("the caliphate").	**- Experimentally introduced pre-exposure.**	Two counter-narratives were introduced:<br><br>Non-Muslims participants **watched two videos**: the first showed a former RWEX describing the process of challenging the tenets of the organisation, before eventually leaving. The second was a government-sponsored cartoon encouraging participants to think critically about racism.<br><br>Muslims participants **watched two videos**: the first showed a former ISEX describing the emotional process of disillusionment with violent jihad, before eventually leaving. The second was a 'behind-the-scenes' of ISEX propaganda, showing their persuasive appeal, and highlighting disillusionment.	Both counter-narratives were designed to **persuade** the audience. The RWEX counter-narratives used **counter-arguments**, **perspective-taking** and **more information** whilst the ISEX counter-narratives used **emotional appeals** and education / **more information**. Both attempted to increase the 'narrativity' of the exposure, driving persuasion.	**Interrupted time series.** Levels of extremism were measured at baseline, post-EX narrative and, finally, post counter-narrative. Participants were allocated to the ISEX or RWEX conditions based on religion (Muslim / non-Muslim) but no comparison were made between groups. Therefore, this study is treated as a controlled before-and-after study.	**Extremist attitudes.**<br>*Un-validated.*<br>Participants indicated their agreement with ten extremist statements (both RWEX and ISEX) on a 7-point scale (-3 = "very strong disagreement", 3 = "very strong agreement"). The scale was used for both groups at three time points: RWEX at baseline (α = .65), post-propaganda (α = .73) and, post-CVE (α = .70) and; ISEX at baseline (α = .59), post-propaganda (α = .71) and, post-CVE (α = .69). All participants, irrespective of exposure, completed both RWEX and ISEX outcome measures.<br><br>*Non-quantitative measure(s).*<br>**Counter-arguments**<br>Participants read the following instruction: "Please list the arguments and justifications you would use to convince someone holding [ISEX/RWEX] attitudes that he or she is wrong. Name at least three arguments that you spontaneously think off".<br><br>*Other outcomes not central to the review research question*[7]<br>**Amplification intentions.**<br>Participants indicated their agreement with two statements *(scale unclear):* ("I would post the video" and "I would watch more videos like this"). The measure was taken from Frischlich et al. (2017). No reliability analysis is provided. Higher numbers indicated higher intentions (p. 6)<br><br>**Attraction to counter-activists / extremists.**<br>*Un-validated.*<br>Participants indicated their agreement with 8 items (e.g. "I can imagine myself supporting the group") on a 7-point Likert scale ranging from 0 ("totally disagree") to 6 ("totally agree"). The same scale was used for both groups post-propaganda (α = .87), and post-CVE (α = .90).	**Extremist attitudes**<br>**1. RWEX**<br>*Post-propaganda (no CN)*<br>M = 1.46<br>SD = 1.20<br>N = 307<br>*Post-CVE (CN)*<br>M = 1.50<br>SD = 1.20<br>N = 311<br><br>**2. ISEX**<br>*Post-propaganda (no CN)*<br>M = 1.59<br>SD = 1.20<br>N = 308<br>*Post-CVE (CN)*<br>M = 1.56<br>SD = 1.15<br>N = 310	*The analysis below was conducted by review authors*[6]<br><br>**1.** Participants reported significantly lower agreement with the RWEX statements post-propaganda compared to baseline (M = 1.69901, SD = 1.15756, N = 322), $t(627) = -2.58$, p = .01. Conversely, post-CVE, participants' agreement increased slightly (M = 1.496, SD = 1.202, N = 311). This difference was not significant.<br><br>**2.** Participants reported the highest agreement with the ISEX statements at baseline (M = 1.622, SD = 1.10, N = 322); the difference between scores at baseline and post-propaganda was not significant, $t(628) = -0.23$, p = .82. Scores between participants post-propaganda and post-CVE were also not significant, $t(616) = 0.24$, p = .81.	**No.**<br>The CVE videos did not effectively reduce participants' agreement with the extremist statements.	**Very low**
	Study 2	N = 157. Participants (M_{age} = 28, SD = 9.63, 44% female) were recruited from cities in Western Germany and included both students (65%) and apprentices. Participants received €15 for participation. Most participants self-identified as Christian (50%), Muslim (28 %), or Atheist and 52% had a first- or second-generation migration background.	*See Study 1.*	*See Study 1.*	*See Study 1.*	*See Study 1.*	*See Study 1.*	**Extremist attitudes.**<br>*Un-validated.*<br>*See Study 1.* The scale was used for both groups at three time points: RWEX at baseline (α = .65), post-propaganda (α = .73) and, post-CVE (α = .70) and; ISEX at baseline (α = .59), post-propaganda (α = .71) and, post-CVE (α = .69).<br><br>*Non-quantitative measure(s).*<br>**Counter-arguments**<br>Participants read the following instruction: "Please list the arguments and justifications you would use to convince someone holding [ISEX/RWEX] attitudes that he or she is wrong. Name at least three arguments that you spontaneously think off".<br><br>*Other outcomes not central to the review research question*[7]<br>**Amplification Intentions.** *See Study 1.*<br>**Attraction to counter-activists / extremists.**<br>*Un-validated.*<br>*See Study 1;* post-propaganda (α = .87), and post-CVE (α = .90).	**Extremist attitudes**<br>**1. RWEX**<br>*Post-propaganda (no CN)*<br>M = 2.63<br>SD = 1.08<br>N =155<br>*Post-CVE (CN)*<br>M = 2.59<br>SD = 1.13<br>N = 155<br><br>**2. ISEX**<br>*Post-CVE (CN)*<br>M = 3.44<br>SD = 1.72<br>N = 155<br>*Post-CVE (CN)*<br>M = 3.42<br>SD = 1.74<br>N = 155	***RWEX.*** Participants reported similar agreement with the RWEX statements post-propaganda compared to baseline (M = 2.77, SD = 1.13, N = 155). Post-CVE, participants' agreement decreased slightly but this difference was not significant, $t(308) = -0.32$, p = .75.<br><br>***ISEX.*** Participants reported the lowest agreement with the ISEX statements at baseline (M = 3.40, SD = 1.13, N = 155); the difference between scores at baseline and post-propaganda were similar. Scores between participants post-propaganda and post-CVE were also similar, $t(308) = -0.10$, p = .91.	**No.**<br>There were no differences in agreement with the extremist statements between the propaganda (no CN) and CVE (CN) conditions.	**Very low**
Riles, Funk & David (2018)		N = 187. Students (M_{age} = 19.47, SD = 1.45, 60% female) were recruited from entry-level undergraduate courses in the United States. *It is not clear if participants received an incentive for*	The dominant narrative amongst the American, undergraduate sample was determined to be that, **in the context of American society and safety, Muslims are dangerous.** Pilot tests conducted pre-experimentally revealed that the most common beliefs about Muslims in the United States group were that many are extremists, that Islam is a religion that celebrates peace, that Muslims are	**- Pilot study**<br>**- Previous research** (cross-sectional)	**Participants watched a clip from the (2007) movie 'The Visitor'** (in which a Syrian-Muslim man is depicted<br>helping an older White man, who is going through a mid-life crisis, learn the djembe drum**) and an episode of Bones (2012) entitled**	Through the use of **pro-social counter-stereotypes**, the study attempted to counter the dominant rhetoric, and show Muslim	**RCT.** Participants were randomly assigned to the experimental ('messages depicting helpful Muslim characters') or	**1. Social Stigma**<br>Participants indicated their agreement with 11 items on a 5-point Likert scale ranging from "strong disagree" to "strongly agree" with stigmatising statements adapted from Smith (2012, p. 530) (α = .89). Higher numbers indicated higher stigma (p. 9).<br><br>**2. Social Distance**	**1. Social Stigma**<br>*Nature video (no CN)*<br>M = 3.54<br>SD = 0.16<br>N = 37[5]<br>*Prosocial (CN)*<br>M = 3.71	*The analysis below was conducted by review authors*[6]<br><br>**1.** Participants reported higher social stigma perceptions in the prosocial condition compared to the control condition. This	**Yes and no.**<br>The use of prosocial videos to counter the perception of Muslims as dangerous was not effective in	**High**

		participating. Those who identified as Muslim were omitted from the analyses (see 'dominant narrative' section).	very devout, that Muslims usually wear some type of head covering, and that Muslims are often terrorists. Unsurprisingly, the internal consistency for this measure was weak (α = .65). However, it does suggest the presence of a dominant, fearful, stigmatising narrative of Muslims amongst the sample, despite some positive items. Furthermore, cited evidence in the study suggests Arab Muslims that they are the most feared socio-cultural group in America by both adults (Bushman & Bonacci, 2004) and children (Brown, Ali, Stone, & Jewell, 2017). They are a group who are routinely viewed as violent perpetrators of terrorism (Dixon & Williams, 2015), and a threat to American society.		**"The Patriot in Purgatory"** (in which a male Iranian-Muslim forensic laboratory assistant helps solve a cold-case involving a United States Army veteran) were shown to participants. In both clips, it was clear that the Muslim protagonists were current, and devout, followers of Islam. Clips ranged from 9 -12 mins and were watched at individual computers, six at a time.	characters aiding non-Muslim characters in "tasks and life-enriching activities" (p. 10)	control/no CN ('nature video') condition.	Participants indicated their agreement with statements such as: "Could you see yourself renting a room in your home to a Muslim person?" and "Could you see yourself spending an evening socializing with a Muslim person?" on a 7-point Likert scale ranging from one ("definitely not") to seven ("definitely yes") (α = .92). Higher values indicated a preference for more distance (p. 11). Four items were adapted from existing scales (see Link et al., 1987; 1999).<br><br>*Other outcomes not central to the review research question.*<br>**Expectancy Value Attitudes Scale**<br>*Un-validated.*<br>Participants indicated their agreement with the most widely-held beliefs about Muslims (determined through the pilot study) on a 7-point Likert scale ranging from −3 to 3 (α = .65). We chose not to include this outcome as the measure included positive widely-held beliefs, and the reliability was low.<br><br>*Non-quantitative measure(s).*<br><br>**Manipulation check**.<br>*Un-validated.*<br>Participants were asked to provide "five thoughts that came to mind when they thought of Muslims"; the key variable researchers were observing was that "Muslims enhance the well-being of those around them". This variable was associated with acceptable Krippendorff's inter-coder reliability (α = .74).	*SD* = 0.08<br>*N* = 150[5]<br><br>**2. Social Distance**<br>*Nature video (no CN)*<br>*M* = 2.74<br>*SD* = 0.21<br>*N* = 37<br>*Prosocial (CN)*<br>*M* = 2.41<br>*SD* = 0.10<br>*N* = 150	difference was significant, $t(185) = -9.20$, $p = <.00$, SMD = 1.69 (95% CI 1.29 to 2.08; v = .04).<br><br>2. Participants reported lower desire for distance in the prosocial condition. This difference was significant, $t(185) = 13.93$, $p = <.00$, SMD = 2.56 (95% CI 2.11 to 3.00; v = .05).<br><br>Due to within-study heterogeneity, these items were not pooled to create a single SMD for this study.	decreasing social stigma. However, it did decrease social distance.	

1 Although the concordant condition is technically 'reinforcing' the dominant narrative (i.e. rendering the character with which participants already agree 'more virtuous'), the authors conclude that, given the evidence of a clear, anti-demonstration narrative in this portion of the sample, the manipulation is likely to be miniscule. Informed by dual process models of cognition, participants' baseline attitudes will make positive information about the concordant character more readily available (i.e. through the availability heuristic). For example, Wilson and Gronke (2000) found that if participants are more "favourably predisposed" towards a political candidate, they are more likely to remember details about that candidate. Through these heuristics, "evidence" (real or imagined) is judged as superior as it is easier to bring to mind (Haddock et al., 1999; Tormala, Petty, & Brinol, 2002). In this case, participants are likely to view the concordant character as virtuous regardless of the manipulation and any effects of the manipulation reflect those which would occur in a non-experimental setting. This is further supported by affirmation of the second hypothesis which found that, irrespective of character virtue, the mean identification level for concordant characters was higher (*M* = 5.11, *SD* = 1.42) than for discordant characters. Therefore, the authors of this review deem it acceptable to use this condition as a comparator.

2 It is presumed that participants in the control condition were not asked this question.

3 Dr Alhabash provided the final sample sizes directly. For the 2012 study, the sample for the implicit (*N*= 63) and explicit (*N* = 68) measures differed from what appeared in the publication. For the 2015 study, the sample for the implicit (*N*= 161) and explicit (*N*= 172) also differed slightly to what was reported in the publication. Dr Alhabash also provided for AMP data in the 2015 study.

4 Author(s) were contacted.

5 Data provided directly by author(s).

6 In some cases, the studies did not conduct means difference test(s). In these cases, the necessary tests were conducted by us, the review authors.

7 These outcomes were not considered proximal to violent radicalisation as, rather than indicating the change in support for the extremist protagonist, they asked participants if they supported the non-extremist "more". These measures are, therefore, more concerned with the persuasiveness of the counter-narrative/CVE videos rather than their effectiveness at reducing susceptibility to violent extremist content.

8 The analysis was conducted by the authors of this review and the results differed slightly, $t(68) = 2.27$, $p = 0.03$.

9 These outcomes were reverse scored for the purpose of the meta-analysis, with higher scores indicating higher negativity of outcomes. To adjust the direction(s) of the necessary outcomes each group's score (in all cases, a Likert-scale average) was subtracted from the maximum possible Likert scale score (in most cases, this was seven to nine).

10 Calculated using the following formula: SD = N x (upper limit – lower limit) / 3.92
