# Supplementary material for: Counter‐narratives for the prevention of violent radicalisation: A systematic review of targeted interventions
Source: Campbell Syst Rev. 2020 Aug 12;16(3):e1106. doi: 10.1002/cl2.1106 (PMC8356325; doi:10.1002/cl2.1106)
Supplement: Supplementary file 3 — Supporting information [file CL2-16-e1106-s001.pdf]

Table D2. *GRADE Quality Assessment*

	**Cernat (2001)**	**Kendrick & Fullerton (2004)**	**Ramasubramanian & Oliver (2007)**	**Gonsalkorale, Allen, Sherman & Klauer (2010)**	**Alhabash & Wise (2012)**	**Alhabash & Wise (2015)**	**Bilewicz & Jaworska (2013)**	**Garagozov (2013)**	**Banas & Richards (2017)**	**Cohen, Tal-Or & Mazor-Tregerman (2015) *Study 1***	**Saleem, Prot, Anderson & Lemieux (2015) *Study 3***	**Bruneau, Lane & Saleem (2017) *Study 1***	**Bruneau, Lane & Saleem (2017) *Study 2***	**Bruneau, Lane & Saleem (2017) *Study 3***	**Cehajic-Clancy & Bilewicz (2017) *Study 1***	**Cehajic-Clancy & Bilewicz (2017) *Study 2***	**Frischlich,. Rieger, Morten, & Bente (2018) *Studiy 1***	**Frischlich,. Rieger, Morten, & Bente (2018) *Study 2***	**Riles, Funk & David (2018)**
**GRADE[1]**	Low[2]	Very low	Moderate	High	Low	Low	Moderate	Moderate	Moderate	Moderate	Moderate	High	High	High	Very low	Very low	Very Low	Very Low	High
**Study Design**	RCT	BA	QRCT	RCT	RCT (interpreted as BA)	RCT (interpreted as BA)	RCT *(with wait-list control)*	RCT	RCT	RCT	RCT	RCT	RCT	RCT	BA	BA	ITS/case series	ITS/case series	RCT
**1. Limitations in the design and implementation of available studies suggesting high likelihood of bias.**	- Small sample size ✗<br>- No baseline measure of outcomes or characteristics ✗<br>- Poor theoretical framework ✗	- No randomisation to conditions ✗<br>- Poor theoretical framework ✗<br>- Without the use of deception, there were likely crossover effects due to the (pre-post) design ✗	- Quasi-random allocation to conditions ✗<br>- Small sample size (*n* = 196) for amount of conditions (eight) ✗	Small sample size (N = 49) for an RCT ✗	- No randomisation to conditions ✗<br><br>- no use of deception so risk of social desirability ✗	- No randomisation to conditions ✗<br><br>- no use of deception so risk of social desirability ✗	- No practice effects due to wait-list control design ✓	- Control participants should have been exposed to a comparator rather than nothing at all ✗	- No baseline measure of outcomes ✗	- No baseline measure of outcomes ✗	- No baseline measure of outcomes ✗<br><br>- Unsupervised viewing of video (i.e. no checks to ensure participants were paying attention) (see Bruneau et al. 2017, p. 748) ✗<br><br>- Participants in the counter-stereotypic video condition rated the video more positively (M = 4.65) than participants in the neutral (M = 3.86) which could explain the difference more-so than the active ingredient(s) in the counter-narrative ✗<br><br>- The study included a no-video condition which allowed us to determine how much of the effectiveness of the intervention was due to the presence or absence of a video. ✓	- No baseline measure of outcomes or characteristics ✗	- Large sample size ✓	- No baseline measure of outcomes or characteristics ✗	- No randomisation to conditions ✗<br><br>- Chance of carryover effects with pre- and post-test design (same measures) ✗<br><br>- Poor reliability for outcomes ✗	- No randomisation to conditions ✗<br><br>- Carryover effects *(see Study 1)* ✗<br><br>- Poor reliability for outcomes ✗	- No randomisation to conditions ✗<br><br>- Social desirability and practice effects. It is highly likely that participants became aware of the true nature of the study through the use of repeated measures. This may have led to social desirability bias (Fisher, 1993) and even boomerang effects (Brehm, 1966) . Furthermore, the use of these measures over 2-3 time points may have led to carryover effects ✗	*See Study 1.*	- No baseline measure of outcomes ✗<br><br>- Unequal sample sizes ✗
**2. Indirectness of evidence (indirect population, intervention, control, outcomes).**	- Ambiguous intervention (lack of descriptions of 'Positive Hungarian' condition, for example) ✗<br>- No reliability analysis ✗	- No control group ✗<br>- Varied population (ethnicity, religious affiliation etc.) ✗<br>- Single item outcome measures. ✗<br>- No reliability analysis ✗	- No baseline measure of prejudice in sample itself ✗	Fine.	- No validation for some outcome measures ✗<br>- similarity of faces in AMP (Palestinian and Israeli ethnicities) ✗	- No validation for some outcome measures ✗<br>- similarity of faces in AMP (Palestinian and Israeli ethnicities) ✗	- There was a contact element to the intervention (Jewish and Polish worked together post-intervention) which makes it difficult to determine the active components of the intervention (i.e. counter-narrative or contact) ✗	- the baseline characteristics of the sample were too varied (e.g. age) ✗	- Crossover effects and indirectness of intervention. See ROB report ✗	- The comparator condition could be seen as an exacerbated dominant narrative (reinforcing, rather than representing, the dominant narrative). Justification for its use is provided under 'dominant narrative' in the data extraction table ✗	- Deception was used and the deception videos (before experimental videos) were counter-balanced (p. 855) ✓	Fine.	Fine.	Fine.	- Intervention was comprised of several components (moral exemplars, critical thinking tasks and contact), making it difficult to determine the "active ingredients" of the intervention ✗	Indirectness of intervention *(see Study 1)* ✗	- accounted for education-level by including both students and apprentices ✓<br><br>- The study could not demonstrate the effectiveness of the propaganda videos compared to baseline and, for this reason, the design did not allow for the counter-narrative to be effectively tested ✗<br><br>- The 'agreement with extremist statements' measure included both RWEX and ISEX statements irrespective of condition. The inclusion of, for example, far right statements in an Islamic extremism condition may have confused participants. Furthermore, these groups tend to denunciate the other (REFS). Therefore, any increase in agreement with one may lead to a decrease in agreement with the other ✗<br><br>- There were validated alternatives to some measures. For example, based on the hypotheses, there are different identification scales which could have been used instead of "attraction" (see Cohen, 2001) ✗<br><br>- It is likely that priming effects occurred following the measurement of counter-arguments before the introduction of the counter-narrative. Therefore, it	*See Study 1.*	Fine.

																	is unclear if the effects of the counter-narrative can be attributed to the intervention, or to the counter-argument measure immediately before ✗<br><br>- Although participants were divided into groups (based on ethnicity), no comparisons were made between groups even though these groups received very different interventions. In other words, scores on the outcome variables were analysed as if participants all received the same propaganda video, and CVE video ✗		
**3. Unexplained heterogeneity or inconsistency of results (including problems with subgroup analyses).**	Fine.	Not reported.	Fine.	Fine (only one outcome)	Heterogeneous findings between implicit bias and attitudes measure ✗	Fine.	Fine.	Fine (only one outcome)	Fine.	Fine.	Fine.	Fine.	Fine.	Slight heterogeneity; different findings on Palestinian violence compared to the other measures ✗	Fine.	Fine.	Fine.	Fine.	Significant heterogeneity on social stigma compared to social distance outcomes ✗
**4. Imprecision of results (wide confidence intervals).**	Fine.	Not reported	Not reported	CI 95% -1.49 to -0.32 ✗	Fine	Fine	Fine.	Not reported	Fine.	Fine.	Fine.	Fine.	Fine.	Fine.	CI 95% -0.55 to -0.09 (pooled outcomes) ✗	CI 95% -0.45 to -0.15 pooled outcomes) ✗	- CI are only provided for moderator analysis (see hypotheses) ✗	- CI are only provided for moderator analysis (see hypotheses) ✗	CI 95% -1.05 to -0.32 ✗
**5. High probability of publication bias.**	Fine. Some non-significant findings reported.	No	No	No	No	No	No	No	No	No.	No.	No.	No.	No.	No.	No,	No.	No.	No.
**1. Large magnitude of effect.**	No effect, *d* = 0.17.	Not reported ✗	Not reported; couldn't calculate effect size ✗	Large, *d* = 0.90 ✓	Small, *d* = -0.37 (pooled outcomes) .	No effect, *d* = 0.11.	Medium, *d* = -0.58 (pooled outcomes) ✓	Not provided for comparisons of interest ✗	Medium, *d* = -0.57 (pooled outcomes) ✓	No effect, *d* = -0.08.	No effect, *d* = -0.02.	Medium, *d* = -0.45 (pooled outcomes) ✓	Medium, *d* = -0.42 (pooled outcomes) ✓	Small, *d* = -0.29 (pooled outcomes).	Small, *d* = -0.23 (pooled outcomes).	Small, *d* = -0.15 (pooled outcomes).	No significant differences found, and not provided.	No significant differences found, and not provided.	Medium, *d* = -0.68 ✓
**2. All plausible confounding would reduce a demonstrated effect or suggest a spurious effect when results show no effect.**	No	No.	No.	No.	No.	No.	No.	No.	No	No.	No.	No.	No.	No.	No.	No.	No.	No.	No.
**3. Dose-response gradient.**	No.	No.	No.	No.	No.	No.	No.	No.	No	No.	No.	No.	No.	No.	No.	No.	No.	No.	No.
**GRADE assessment of study limitations**	Double downgraded RCT.	Double downgraded observational.	Downgraded RCT.	No serious limitations, do not downgrade.	No serious limitations, do not downgrade.	No serious limitations, do not downgrade.	No serious limitations, do not downgrade.	Downgraded RCT.	Downgraded RCT.	Downgraded RCT.	Downgraded RCT.	No serious limitations, do not downgrade.	No serious limitations, do not downgrade.	No serious limitations, do not downgrade.	Downgraded observational.	Downgraded observational.	Case series.	Case series.	No serious limitations, do not downgrade.

1 **High** (RCT); **Moderate** (downgraded RCT / upgraded observational); **Low** (double downgraded RCT / observational); **Very low** (triple downgraded RCT / downgraded observational study / case-series)

2Study was downgraded from 'moderate' to 'low' after consultation with second coder (KC).
